# Boswellianols A–I, Structurally Diverse Diterpenoids from the Oleo-Gum Resin of *Boswellia carterii* and Their TGF-*β* Inhibition Activity

**DOI:** 10.3390/plants13081074

**Published:** 2024-04-11

**Authors:** Zhi-Rong Lin, Meng-Yu Bao, Hao-Ming Xiong, Dai Cao, Li-Ping Bai, Wei Zhang, Cheng-Yu Chen, Zhi-Hong Jiang, Guo-Yuan Zhu

**Affiliations:** 1 State Key Laboratory of Quality Research in Chinese Medicines, Guangdong-Hong Kong-Macao Joint Laboratory of Respiratory Infectious Disease, Macau Institute for Applied Research in Medicine and Health, Macau University of Science and Technology, Macau 999078, China; thomas05@sina.cn (Z.-R.L.); 3220004965@student.must.edu.mo (M.-Y.B.); 3230006692@student.must.edu.mo (H.-M.X.); caodai8989@163.com (D.C.); lpbai@must.edu.mo (L.-P.B.); wzhang@must.edu.mo (W.Z.); 2 Jiaheng Pharmaceutical Technology Co., Ltd., Zhuhai 519000, China; yanfachenchengyu@fusenpharma.com

**Keywords:** *Boswellia carterii*, diterpenoids, boswellianols A–I, TGF-*β*

## Abstract

Olibanum, a golden oleo-gum resin from species in the *Boswellia* genus (*Burseraceae* family), is a famous traditional herbal medicine widely used around the world. Previous phytochemical studies mainly focused on the non-polar fractions of olibanum. In this study, nine novel diterpenoids, boswellianols A–I (**1**–**9**), and three known compounds were isolated from the polar methanolic fraction of the oleo-gum resin of *Boswellia carterii*. Their structures were determined through comprehensive spectroscopic analysis as well as experimental and calculated electronic circular dichroism (ECD) data comparison. Compound **1** is a novel diterpenoid possessing an undescribed prenylmaaliane-type skeleton with a 6/6/3 tricyclic system. Compounds **2**–**4** were unusual prenylaromadendrane-type diterpenoids, and compounds **5**–**9** were new highly oxidized cembrane-type diterpenoids. Compounds **1** and **5** showed significant transforming growth factor *β* (TGF-*β*) inhibitory activity via inhibiting the TGF-*β*-induced phosphorylation of Smad3 and the expression of fibronectin and N-cadherin (the biomarker of the epithelial–mesenchymal transition) in a dose-dependent manner in LX-2 human hepatic stellate cells, indicating that compounds **1** and **5** should be potential anti-fibrosis agents. These findings give a new insight into the chemical constituents of the polar fraction of olibanum and their inhibitory activities on the TGF-*β*/Smad signaling pathway.

## 1. Introduction

Olibanum is the shining and golden oleo-gum resin collected from the trunk incisions of species from the *Boswellia* genus (*Burseraceae* family), which comprises 24 accepted species and is distributed in the dry and tropical regions of Asia and Africa [1]. *Boswellia carterii* and *Boswellia hhaw-dajiana*, native to Somalia and Ethiopia along the Red Sea [2], are officially regarded as the medicinal material olibanum according to Pharmacopoeia of the People’s Republic of China. Olibanum has been used worldwide as a traditional herbal medicine to treat swelling and pain from injuries, rheumatoid arthritis, and dysmenorrhea for thousands of years [3,4,5,6]. Olibanum extract has shown its hepatoprotective activity by inhibiting liver fibrosis and downregulating the expression of cytokines including transforming growth factor *β* (TGF-*β*), cyclooxygenase-2 (COX-2), and tumor necrosis factor-*α* (TNF-*α*) [7]. Olibanum combined with other herbs has also been reported to treat pulmonary and interstitial fibrosis by inhibiting the TGF-*β*/Smad pathway [8,9]. In addition, *β*-boswellic acid, a main triterpenoid from olibanum, showed anti-inflammation, anti-tumor, and anti-interstitial fibrosis properties, partly involving the Klotho/TGF-*β*/Smad signaling pathway [10,11,12,13].

Previous phytochemical studies have reported hundreds of diterpenes and triterpenes from olibanum [14,15]. In the last decade, a substantial number of separation studies has been performed on olibanum examining the non-polar fractions of ethanol extract that enrich triterpenoids, macrocyclic cembrane-type diterpenoids, and 5/7/3 tricyclic prenylaromadendrane-type diterpenoids [16,17,18]. Cemrbane-type diterpenoids, characterized by a 1,12-oxolane ring, have displayed numerous activities including hepatoprotection, neuroprotection, anti-tumor, and anti-inflammatory activities [19,20,21]. Prenylaromadendrane-type diterpenoids showed their hepatoprotective and anti-inflammatory activity as well [16,22]. However, the study on chemical constituents from the polar fraction of olibanum is rare [23], and the effects of the diterpenoids of olibanum on the TGF-*β*/Smad signaling pathway have not yet been reported.

In this study, the more polar methanolic fraction was subjected to chromatographic separation, which resulted in the isolation and identification of one new diterpenoid with a novel prenylmaaliane-type skeleton (**1**), three new prenylaromadendrane-type diterpenoids (**2**–**4**), four new cembrane-type diterpenoids (**5**–**9**), and three known compounds (**10**–**12**). As one part of our ongoing screening of lead compounds for inhibiting TGF-*β* from natural products [24,25,26], the TGF-*β* inhibitory activities of isolated compounds were evaluated in LX-2 human hepatic stellate cells, and the results showed that compounds **1** and **5** exhibited TGF-*β*-inhibitory activities.

## 2. Results and Discussion

### 2.1. Chemical Constituents from Olibanum of Boswellia carterii

**Figure 1:**
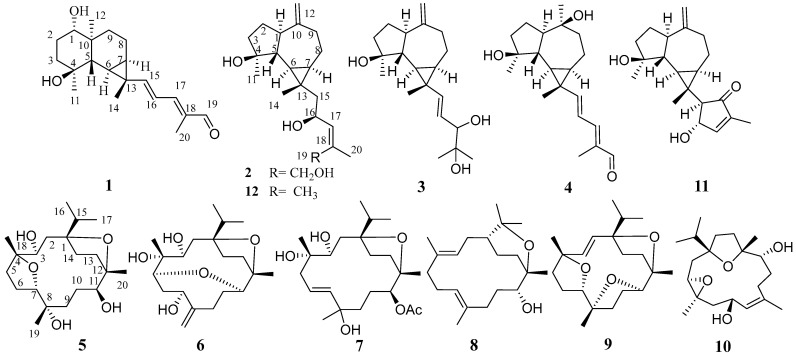
Chemical structures of compounds **1**–**12**.

Olibanum was sequentially extracted with petroleum ether and methanol to provide non-polar and polar fractions. The polar methanolic fraction was then separated by silica gel column chromatography and further purified by MPLC and semi-preparative HPLC to obtain 12 diterpenoids (Figure 1), including nine undescribed diterpenoids, named boswellianols A–I (**1**–**9**). Three known compounds were identified as boscartin D (**10**) [27], boscartol O (**11**) [22], and cneorubin A (**12**) [28] by comparing their NMR data with those of compounds reported in the literature.

**Figure 2:**
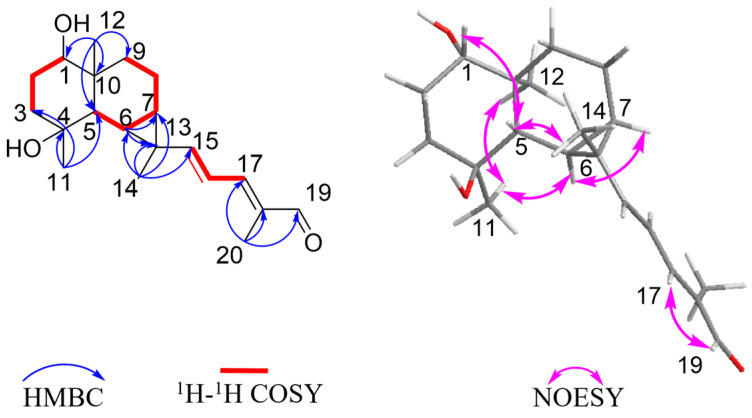
Key ^1^H–^1^H COSY, HMBC, and NOESY correlations of compound **1**.

Boswellianol A (**1**) was isolated as a yellow oil with a molecular formula C_20_H_30_O_3_ determined by HR-ESI-MS ion at *m*/*z* 319.2259 [M + H]^+^ (calculated to be 319.2268), yielding six degrees of unsaturation. The ^1^H spectrum of **1** (Table 1) showed characteristic resonances for the following: four methyl groups at *δ*_H_ 0.91 (3H, s, Me-12), 1.19 (3H, s, Me-14), 1.30 (3H, s, Me-11), and 1.84 (3H, s, Me-20); three olefinic protons at *δ*_H_ 5.80 (1H, d, *J* = 15.0 Hz, H-15), 6.42 (1H, dd, *J* = 15.0, 11.6 Hz, H-16), and 6.79 (1H, d, *J* = 11.6 Hz, H-17); and an aldehyde proton at *δ*_H_ 9.40 (1H, s, H-19). The ^13^C and DEPT 135 spectra showed 20 carbon signals (Table 2) corresponding to four methyls, four methylenes, eight methines [including three alkenyl sp^2^ carbons (*δ*_C_ 156.5, 120.3, and 150.0), one aldehyde carbon (*δ*_C_ 195.0), and one oxygenated sp3 carbon (*δ*_C_ 78.3)], and four quaternary carbons [including an alkenyl sp^2^ carbon (*δ*_C_ 138.4) and an oxygenated sp^3^ carbon (*δ*_C_ 72.1)]. The above spectroscopic data indicated that **1** is a diterpenoid with three rings (one aldehyde and two double bonds occupying three of the six degrees of unsaturation). The planar structure of **1** was elucidated by the analysis of HSQC, HMBC, and ^1^H–^1^H COSY spectra (Figure 2). Two spin-coupling systems were first established by ^1^H–^1^H COSY correlations of H-1/H_2_-2/H_2_-3 and H-5/H-6/H-7/H_2_-8/H_2_-9, shown as bold lines in Figure 2. HMBC correlations from Me-11 to C-3/C-4 /C-5 revealed the connectivity of C3–C4–C5. A 6/6 ring system was then assigned by HMBC correlations from Me-12 to C-1/C-5/C-9/C-10. A cyclopropyl moiety was indicated by the characteristic relatively shielded signals of C-6, C-7, and C-13 [*δ*_C_ 22.4 (C-6), 24.0 (C-7), 25.7 (C-7); *δ*_H_ 1.20 (m, H-6), 1.07 (m, H-7)] and the HMBC correlations from Me-14 to C-6/C-7/C-13 and H-6 to C-7/C-13, which further formed a 1,4-dihydroxy-maaliane fragment with a 6/6/3 ring system [29]. In addition, a prenylaldehyde moiety was suggested by the HMBC correlations from Me-20 to C-19 (*δ*_C_ 195.0)/C-18 (*δ*_C_ 134.8)/C-17 (*δ*_C_ 150.0) and ^1^H–^1^H COSY resonances of H-17/H-16 (Figure 2). The NMR data on the prenylaldehyde moiety in **1** were consistent with those of boscartol F [16], further confirming the presence of the *α*, *β*, *γ*, *δ*-unsaturated aldehyde in **1**. The 1,4-dihydroxy-maaliane unit and prenylaldehyde moiety were connected by C-15 (*δ*_C_ 156.5) and C-16 (*δ*_C_ 120.3), corresponding to the ^1^H–^1^H COSY correlation of olefinic protons H-15/H-16 (Figure 2). The aforementioned data analysis elucidated that compound **1** is a new diterpenoid with an undescribed prenylmaaliane skeleton.

**Figure 3:**
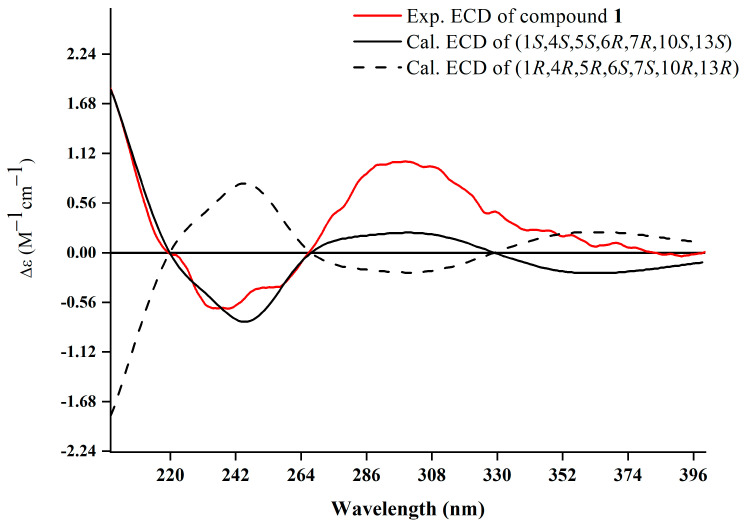
Experimental and calculated ECD spectra of **1**.

The relative configuration was further analyzed by the NOESY spectrum. The NOESY cross peaks of Me-11/Me-12, Me-11/H-6, and H-6/H-7 indicated that H-6, H-7, Me-11, and Me-12 are arranged in an *α*-orientation, based on the same H-6*α* and H-7*α* of the cyclopropane unit in prenylaromadendrane-type diterpenoids [22,30] isolated from the *Boswellia* genus. NOESY interactions between H-1/H-5 and H-5/Me-14 suggested the *β*-orientation for H-1, H-5, and Me-14. The 15*E* and 17*E* were assigned by the large coupling constant (*J*_H-15, H-16_ = 15.0 Hz) and the correlation between H-17 and H-19 in the NOESY spectrum, respectively. The absolute configuration of **1** was deduced as 1*S*,4*S*,5*S*,6*R*,7*R*,10*S*,13*S* by comparing experimental electronic circular dichroism (ECD) recorded in MeOH with the calculated ECD spectra of the two possible stereoisomers by TD-DFT (Figure 3). Thus, the structure of **1** was established as shown.

**Figure 4:**
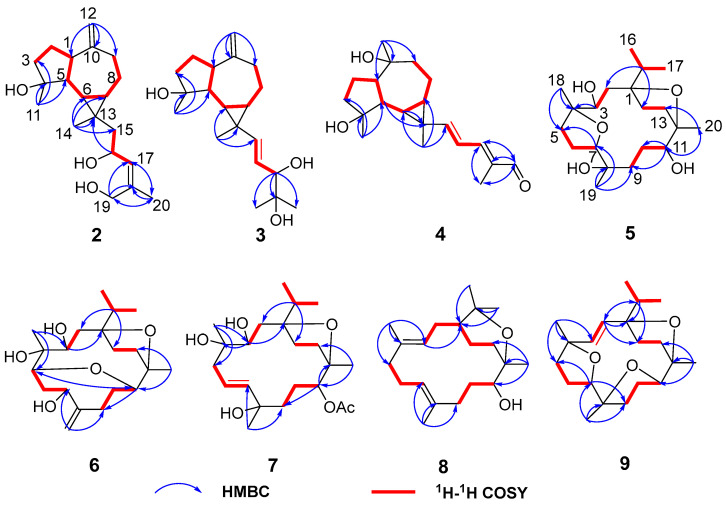
Key ^1^H–^1^H COSY and HMBC correlations of compounds **2**–**9**.

**Figure 5:**
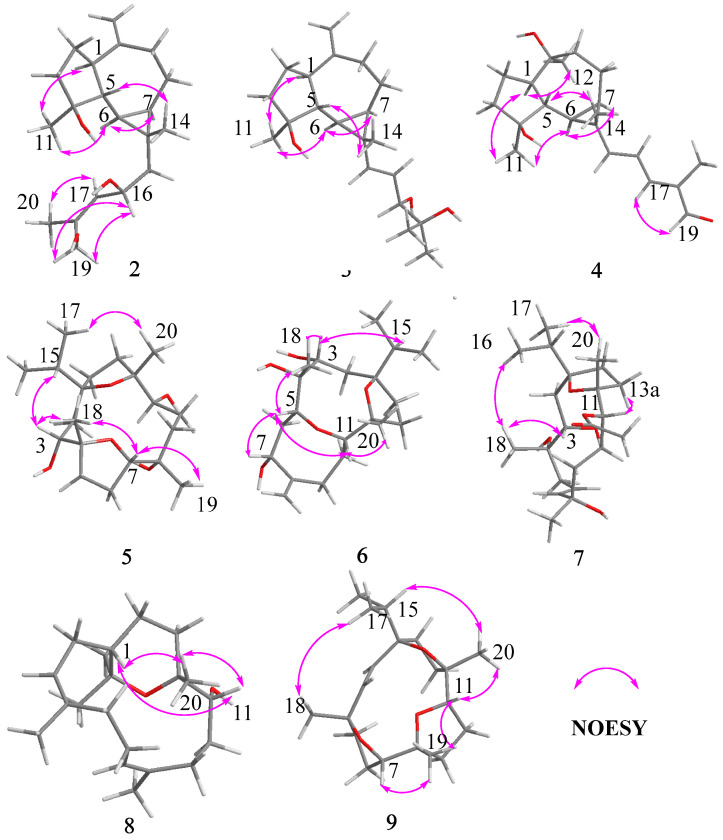
Key NOESY correlations of compounds **2**–**9**.

Compound **2** was obtained as a white amorphous solid with the molecular formula C_20_H_32_O_3_ determined by HR-ESI-MS ion at *m*/*z* 343.2232 [M + Na]^+^ (calculated for C_20_H_32_NaO_3_ as 343.2244) and 321.2410 [M + H]^+^ (calculated for C_20_H_33_O_3_ as 321.2424). Its ^1^H NMR spectrum (Table 1) displayed three methyl singlets at *δ*_H_ 1.10 (3H, s, Me-14), 1.28 (3H, s, Me-11), and 1.82 (3H, s, Me-20), two vinyl singlets at *δ*_H_ 4.70 (1H, s, H-12a) and 4.72 (1H, s, H-12b), an olefinic doublet at *δ*_H_ 5.34 (1H, d, *J* = 8.0 Hz, H-17), and a proton at oxygen-bearing methine at *δ*_H_ 4.69 (1H, overlap, H-16). ^13^C NMR and DEPT 135 spectra showed 20 carbon resonances (Table 2) including two double bonds at *δ*_C_ 106.6, 131.1, 138.6, and 153.5, a secondary alcohol at *δ*_C_ 62.1, and a quaternary oxygenated carbon at *δ*_C_ 80.7. The ^1^H and ^13^C NMR data of **2** (Table 1 and Table 2) were similar to those of cneorubin A (**12**), a known prenylaromadendrane-type [28] diterpenoid from olibanum, except that a hydroxymethyl group [*δ*_C_ 62.1 (C-19), *δ*_H_ 4.21 (d, *J* = 12.2 Hz, H-19a), 4.08 (d, *J* = 12.2 Hz, H-19b)] in **2** replaced the methyl group (C-19) in **12**. HMBC correlation (Figure 4) from H-19 to C-17/C-18/C-20 confirmed the hydroxymethyl group at C-19 in **2**. Detailed 2D NMR data analysis further determined the planar structure of **2**. The NOE enhancement of Me-14/H-5 indicated that Me-14 and H-5 were *β*-oriented, while H-1, H-6, H-7, and Me-11 were *α*-oriented according the NOESY correlations of H-1/H-6, H-6/H-7, and H-6/Me-11 (Figure 5). It was observed that the NMR data of C-16 to C-19 in **2** and the large coupling constant (*J* = 8.0 Hz) between H-16 and H-17 were identical with those of boscartol E, a similar compound with the same prenyl moiety isolated from this plant [16]. Considering the same skeleton and biogenetic pathway, the orientation of 16-OH was deduced as 16-*β*-OH, which is the same as that of boscartol E [16]. The 17-*Z* configuration was assigned by the NOESY correlations of H_2_-19/H-16 and H-17/Me-20 (Figure 5) as well as the similar NMR data of C-16 to C-19 between **2** and boscartol E [16]. Thus, the structure of **2** was established as shown and named boswellianol B.

**Figure 6:**
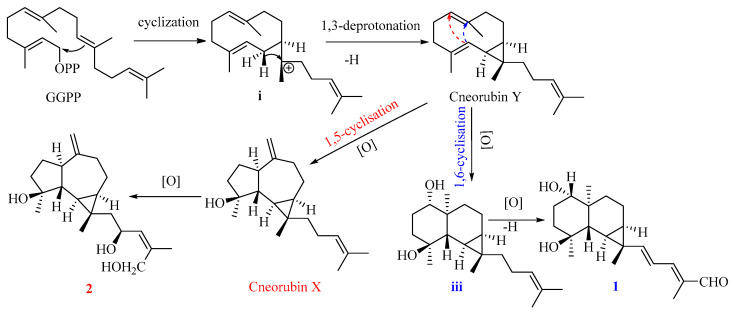
The plausible biosynthesis pathway of compounds **1** and **2**.

Compound **1** is the first example of a prenylmaaliane-type diterpenoid. The biosynthesis pathway of prenylaromadendrane-type diterpenoids is unclear. It was proposed (Figure 5) that compounds **1** and **2** could originate from the same intermediate cneorubin Y, a bicyclogermacrene-type diterpenoid, which should be formed from geranylgeranyl pyrophosphate (GGPP) by enzyme-catalyzed cyclization and deprotonation [31,32]. The 1,3-deprotonation and cyclization of intermediate **i** could be essential steps to construct the primary scaffold with a cyclopropane ring in these diterpenoids. The key step to form the 6,6-bicyclic system of the prenylmaaliane-type intermediate **iii** from cneorubin Y by oxygenation and 1,6-cyclisation (shown as the blue arrow in Figure 6) would be similar to the reported enzyme-catalyzed cyclization to generate the maaliane-type sesquiterpene skeleton from bicyclogermacrene-type sesquiterpenoids [33,34]. Subsequently, the intermediate iii may undergo further oxygenation and deprotonation to form compound **1**. On the other hand, the oxygenation, 1,5-cyclisation(shown as the red arrow in Figure 6), and double bond rearrangement of cneorubin Y could produce the prenylaromadendrane-type diterpenoid, cneorubin X [33,34], which could be further oxidized to form compound **2** (Figure 6).

Compound **3** shared the same formula as 2 deduced by its sodium adduct ion at *m*/*z* 343.2250 [M + Na]^+^ (calculated for C_20_H_32_NaO_3_ as 343.2244) and protonated ion at *m*/*z* 321.2411 [M + H]^+^ (calculated for C_20_H_33_O_3_ as 321.2424), indicating that **3** could be an isomer of **2** bearing the same prenylaromadendrane skeleton. The ^1^H and ^13^C NMR spectrum of **3** showed similar proton and carbon chemical shifts of aromadendrane moiety with **2** (Table 1 and Table 2). The major difference between the two compounds was the prenyl group side chain. The 2,3-dihydroxy-2-methylpent-4-ene side chain in **3** was established by ^1^H–^1^H COSY correlation between H-15 (*δ*_H_ 5.36, d, *J* = 15.5 Hz), H-16 (*δ*_H_, 5.41, dd, *J* = 15.5, 7.6 Hz), and H-17 [*δ*_H_ 3.84 (1H, d, *J* = 7.6 Hz), as well as HMBC correlations from H-17 to C-18/C-19/C-20 (Figure 4). The NOESY correlations of Me-14/H-5, H-6/H-7, and Me-11/ H-1, H-6 (Figure 5) suggested that the relative configuration of the aromadendrane moiety in **3** was the same as that of **2**. Because of the free rotation of the prenyl moiety, the configuration of C-17 was not determined in this study. Therefore, the structure of **3** was determined as shown and named boswellianol C.

Boswellianol D (**4**) has the same molecular formula of C_20_H_30_O_3_ as **1**. The 1D and 2D NMR data demonstrated that **4** shared the same prenylaromadendrane skeleton as **2** and **3**. The presence of the four methyl singlets, a quaternary oxygenated carbon at C-10 (*δ*_C_ 74.8), and the absence of two terminal olefinic methylene protons in **4** showed that the exocyclic double bonds at C-10 of **2** and **3** were oxidized, which was proved by HMBC correlations from Me-12 (*δ*_H_ 1.24, s) to C-1, C-9, and C-10. The remarkable *α*, *β*, *γ*, *δ*-unsaturated aldehyde fragment was established by the ^1^H–^1^H COSY correlation of H-15/H-16/H-17, and key HMBC correlation from H-19 to C-17/C-18/C-20 and H-20 to C-17/C-18/C-19 (Figure 4). The 15*E* double bond was confirmed by the big coupling constants (*J*_15,16_ = 15.1 Hz). The 17*E* was suggested by the NOE correlation of H-17/H-19 and the NMR data comparison with those of **1** and boscartol F [16]. The relative configuration of compound **4** was defined by NOESY interactions of H-1/H-11, H-12 and H-6/H-1, H-7 suggesting the same *α*-orientation as **2** and **3** (Figure 5).

The molecular formula of boswellianol E (**5**) was deduced as C_20_H_36_O_5_ by HR-ESI-MS [M + Na]^+^ ion peak at *m*/*z* 379. 2452 (calculated to be 379.2455), yielding three degrees of unsaturation. The ^1^H NMR spectrum (Table 3) revealed three methyl singlets [*δ*_H_ 1.12 (3H, s, Me-20), 1.16 (3H, s, Me-19), and 1.18 (3H, s, Me-18)], two methyl doublets [*δ*_H_ 0.94 (3H, d, *J* = 6.8 Hz, H-17) and 0.90 (3H, d, *J* = 6.8 Hz, Me-16)], and three protons at oxygen-bearing methines [*δ*_H_ 3.81 (1H, d, *J* = 7.2 Hz, H-3), 3.90 (1H, dd, *J* = 10.6, 5.0 Hz, H-7), and 4.25 (1H, dd, *J* = 11.4, 2.7 Hz, H-11)]. The ^13^C, HSQC, and DEPT 135 spectra gave five methyls, four methines, seven methylenes, and four quaternary carbons. By comparison with the MS and NMR data of boscartin R (a cembrane-type diterpenoid from olibanum) [19], it was believed that **5** was the 11-deacetylation product of boscartin R. The 2D NMR data analysis (Figure 4 and Figure 5) further established the structure of **5** as shown.

The molecular formula of boswellianol F (**6**) was confirmed as C_20_H_34_O_5_ by 20 signals in the ^13^C NMR spectrum and protonated molecules at *m*/*z* 355.2478 [M + H]^+^ (calculated as 355.2479 for C_20_H_35_O_5_) in the HR-ESI-MS spectrum, implying four indices of hydrogen deficiency. The ^1^H and ^13^C NMR data of **6** (Table 1 and Table 3) showed four protons at oxygen-bearing methines [*δ*_H_ 3.75 (1H, dd, *J* = 3.3, 2.6 Hz, H-3), 4.30 (1H, dd, *J* = 10.6, 4.7 Hz, H-7), *δ*_H_ 3.21 (1H, d, *J* = 10.0 Hz, H-5), and *δ*_H_ 3.27 (1H, dd, *J* = 11.4, 2.6 Hz, H-11)] and three quaternary oxygenated carbons at *δ*_C_ 87.7 (C-1), 76.7 (C-4), and 85.7 (C-12), indicating that **6** was also a highly oxidized cembrane-type diterpenoid. Two conspicuous olefinic singlets at *δ*_H_ 5.05 (1H, s, H-19a) and *δ*_H_ 5.30 (1H, s, H-19b) and two olefinic carbons at *δ*_C_ 116.2 (C-19) and 152.5 (C-8) revealed that a methyl group was reduced to an alkenyl group in the cembrane-scaffold. The double bond and the ring of the cembrane skeleton occupied two of four degrees of unsaturation, suggesting two oxygen-bridged rings in **6**. By comparison with **5**, the major difference is that a tetrahydrofuran ring (C4–O–C7) in **5** was replaced with an 8 membered oxane-ring (C5–O–C11) in **6**, deduced by HMBC correlations from H-11 to C-5 and H-5 to C-11. This 8 membered oxane-ring moiety was first reported in cembranoids isolated from the *Boswellia* genus. Additionally, the planar structure of **6** was determined by HMBC and ^1^H–^1^H COSY correlations (Figure 4). The NOESY cross peaks of H-3/Me-18, H-15, H-5/H-7, Me-18, H-11, and H-11/H-5, Me-20 exhibited that all the above protons at asymmetric centers were cofacial in the same *β*-orientation (Figure 5). Thus, the structure of **6** was elucidated as shown in Figure 1.

Compound **7** was obtained as a white amorphous solid with a molecular formula of C_22_H_38_O_6_ established by HR-ESI-MS sodium adduct ion at *m*/*z* 421.2540 (calculated as 421.2561 for C_22_H_38_NaO_6_). The ^1^H and ^13^C NMR data of **7** (Table 2 and Table 3) were similar to those of boscartin Z [19], except for the absence of the ketone (C-5) and the C3–C4 double bond of boscartin Z and the presence of two hydroxy groups at C-3 (*δ*_C_ 71.0) and C-4 (*δ*_C_ 75.1) in 7, which was supported by the HMBC correlations from Me-18 to C-3 and C-5 and from H-3 to C-1 and C-4 (Figure 4), as well as the molecular formula of **7**. The double bond [*δ*_H_ 5.56 (1H, td, *J* = 15.8, 7.0 Hz, H-6) and 5.77 (1H, td, *J* = 15.9, 1.4 Hz, H-7)] was assigned at the C-6 and C-7 positions based on ^1^H–^1^H COSY correlations of H-5/H-6/H-7 and the HMBC correlation from Me-19 to C-7, C-8, and C-9 (Figure 4). It has been reported that cembrane-type diterpenoids with 3-*α*-OH and quaternary C-4 have the smaller coupling between H-3 and H-2 (*J*_H-2, H-3_ = ~7.6–8.8 Hz) than those with 3-*β*-OH (*J*_H-2, H-3_ = 10.0–10.8 Hz) isolated from the *Boswellia* genus [15,19]. Considering the similar coupling constant between H-3 and H-2 (*J*_H-2, H-3_ = 8.5 Hz in **7**) and biogenesis, H-3*β* of **7** was deduced. The obvious NOE correlations of H-11/H-13a, Me-20/Me-16, and H-3/Me-18 (Figure 5), and the absence of the NOE correlation of H-11/Me-20 in the NOESY spectrum of **7,** as well as the similar C-11–C-13 NMR data with those of boscartin Z [19], suggested that the Me-20, Me-18, and 11-OAc group were in *β*-orientation in **7**. The orientation of Me-19 was not determined because of a lack of convincing evidence. Therefore, the structure of **7** was established as shown and named boswellianol G.

Boswellianol H (**8**) was isolated as a yellow oil, whose chemical formula was deduced as C_20_H_35_O_2_ from HR-ESI-MS ion at *m*/*z* 307.2637 ([M + H]^+^, calculated as 307.2632), yielding 4 degrees of unsaturation. The ^1^H and ^13^C NMR data of **8** (Table 2 and Table 3) were close to those of decaryiol [35,36], a cembranoid bearing a C12–O–C15 tetrahydropyran ring isolated from soft coral. The recognizable difference from decaryiol was the hydroxy group position. The ^1^H–^1^H COSY correlations of H-11/H-10/H-9 and HMBC correlations from Me-20 to C-11, C-12, and C-13 indicated that the hydroxy group was located at C-11 in **8** (Figure 4). The NOESY interactions of H-11/Me-20, H-11/H-1, and Me-20/H-1 (Figure 5) suggested the *β*-orientation of H-1, H-11, and Me-20, which is similar to those of decaryiol [35,36].

Boswellianol I (**9**), a yellow amorphous solid, has the molecular formula of C_20_H_32_O_3_ determined by HR-ESI-MS ion at *m*/*z* 321.2427 ([M + H]^+^, calculated as 321.2424) with 5 degrees of unsaturation. Compound **9** has two less oxygens and two more degrees of unsaturation than **5**. The ^1^H and ^13^C NMR data of **9** (Table 2 and Table 3) were similar to those of **5**, except that a double bond [*δ*_H_ 5.23 (1H, d, *J* = 16.3 Hz, H-3) and 5.55 (1H, d, *J* = 16.3 Hz, H-2)] was observed in **9**. The HMBC correlations from Me-18 to C-3/C-4/C-5 and from H-2 to C-1/C-14/C-15 fixed the location of the double bond at C-2/C-3. The remaining one degree of unsaturation in **9** results from the additional tetrahydrofuran ring (C11–O–C8), which was verified by HMBC correlation from H-11 to C-8 (Figure 4). The *β*-orientations of H-7, H-11, Me-18, Me-19, Me-20, and the isopropyl group were assigned by the cross peaks of H-15/Me-20, Me-20/H-11, Me-19/H-11, Me-19/H-7, and Me-17/Me-18 in the NOESY spectrum (Figure 5). Thus, the structure of **9** was established as shown (Figure 1).

### 2.2. TGF-β Inhibition Assessments of the Isolated Compounds

**Figure 7:**
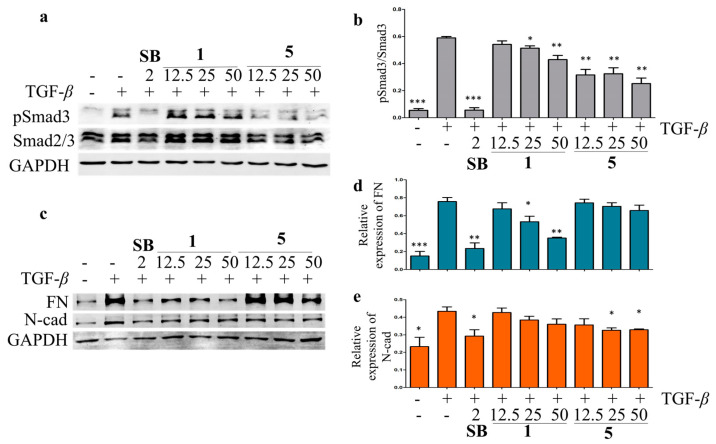
The inhibitory effects of compounds **1** and **5** on TGF-*β*/Smad signal pathway in LX-2 cells. The effects of compounds **1**, **5**, and SB-431542 (positive control) on Smad3 phosphorylation (**a**) and expression of EMT-related proteins, including FN and N-cad (**c**) in the TGF-*β*-induced LX-2 cells by Western blot. The rate of Smad3 phosphorylation (**b**), relative expression of FN (**d**), and N-cad (**e**) were calculated as the means ± SD (*n* = 3). Statistical significance was assessed by *t*-test for two groups comparison. * *p* < 0.05, ** *p* < 0.01, and *** *p* < 0.001 compared to the TGF-*β*-induced group.

Liver fibrosis is the result of a chronic wound-repairing response after injury [37]. While injured by viruses, toxins, or cholestasis, the quiescent hepatic stellate cells (HSCs) are targeted by profibrogenic cytokines TGF-*β* and activated as myofibroblasts to produce fibrotic-related marker collagen type I, fibronectin (FN), N-cadherin (N-cad), and *α*-smooth muscle actin [38]. The hepatoprotective activity against the hepatic fibrosis of the isolated compounds was evaluated in TGF-*β*-induced LX-2 HSCs by Western blot. The results showed that compounds **1**, **5**, **9**, and **11** inhibited the TGF-*β*-induced Smad3 phosphorylation at 25 μM in LX-2 cells (Figure S89). Compounds **1** and **5** were then chosen for the further analysis of their effects on the TGF signaling pathway at different concentrations, using SB-431542 (a TGF-*β* inhibitor) as a positive control. Compound **5** showed significant inhibitory effects on the TGF-*β*-induced Smad3 phosphorylation at 12.5, 25, and 50 μM, while a weaker inhibitory effect of **1** on the TGF-*β*-induced Smad3 phosphorylation was observed in LX-2 cells (Figure 7). The TGF-*β*-induced upregulation of N-cad and FN was suppressed by **1** and **5** in a dose-dependent manner (Figure 6). These data indicated that **1** and **5** could inhibit the LX-2 cell epithelial–mesenchymal transition by inhibiting the TGF/Smad signaling pathway and should be potential anti-fibrosis agents.

## 3. Material and Method

### 3.1. General Experimental Procedures

Optical rotations were measured on a Rudolph Research Analytical Autopol I (Rudolph, Hackettstown, USA) automatic polarimeter. UV spectra and ECD spectra were detected on a JASCO High Performance J-1500 CD spectrometer (JASCO, Tokyo, Japan). NMR spectra were obtained at 600 MHz for ^1^H NMR and 150 MHz for ^13^C NMR, respectively, on a Bruker Ascend 600 spectrometer (Bruker, Ettlingen, Germany) using TMS as an internal standard. IR spectra were obtained on a Shimadzu IR Affinity 1S spectrometer (Shimadzu, Kyoto, Japan). The Agilent 6230 MS spectrometer (Agilent, Santa Clara, CA, USA) was employed to perform high-resolution electrospray ionization mass spectrometry (HR-ESI-MS) analysis. HPLC purifications were carried out on a Waters 1525 pump system (Waters, Milford, MA, USA)equipped with a Waters 2489 UV/Vis detector(Waters, Milford, MA, USA), an Agilent 1260 pump system with an Agilent 1200 DAD detector (Agilent, Santa Clara, CA, USA), and a Thermo Fisher Ultimate 3000 RSLC system (Thermo, Waltham, MA, USA) using Waters XBridge C18 (5 μm, 250 × 10 mm, i.d.), Xselect CSH C18 (5 μm, 250 × 10 mm, i.d.), and T3 OBD (5 μm, 250 × 10 mm, i.d.) columns. Medium-pressure liquid chromatography (MPLC) was performed on a Buchi C-620 Sepacore Flash Chromatography System (Buchi, Flawil, Switzerland) equipped with a Siliabond C18 column (Unisil ODS gel, 10 μm, 36 × 460 mm, i.d.) with a flowrate of 20 mL/min and MCI column (Unips PS/DVB, 50 μm, 46 × 460 mm i.d.) with a flowrate of 50 mL/min and UV detection at 210 nm. Column chromatography (CC) was conducted using silica gel (200–400 mesh, Qingdao Haiyang Company, Qingdao, China) as the packing material.

### 3.2. Plant Resin Material

The oleo-gum resin of *B. carterii* was purchased in October 2019 from Bozhou Chinese Medicine Material Market, Anhui Province, China, and identified by Dr. G.-Y. Zhu. A voucher specimen (BC-202010) was preserved at the State Key Laboratory of Quality Research in Chinese Medicine, Macau University of Science and Technology.

### 3.3. Extraction and Isolation

The gum resin of *B. carterii* (4.7 kg) was first powdered and extracted sequentially with petroleum ether and methanol to obtain two fractions. The methanolic extract (607.0 g) was chromatographed on a silica gel CC eluted by gradient PE–EtOAc–MeOH (100:0:0–0:100:0–0:0:100) to obtain eight fractions (Fr.1–Fr.8). The Fr. 4 (103.0 g) was separated by MPLC equipped with an MCI column using a gradient mobile phase of MeCN–H_2_O (40:60–0:100) to give eight fractions (Fr.4-a–Fr.4-h). Fr.4-a (2.9 g) was further separated by MPLC on a C18 column eluted with MeCN–H_2_O (38:62–0:100) to obtain 11 sub-fractions (Fr.4-a-1–Fr.4-a-11). Fr.4-a-9 (202.0 mg) was subjected to the HPLC eluted with MeOH–H_2_O (25:75) at a flow rate of 3.0 mL/min to give **5** (2.5 mg, t_R_ = 23.3 min). 

Fr.4-b (3.3 g) was separated by MPLC on a C18 column eluted with MeCN–H_2_O (40:60–0:100) to obtain nine sub-fractions (Fr.4-b-1–Fr.4-a-9). Fr.4-b-4 (214.0 mg) was purified by HPLC eluted by MeOH-H_2_O (55:45) to produce **10** (9.0 mg, t_R_ = 18.5 min). Fr.4-b-6 (147.0 mg) was purified by HPLC eluted with MeCN–H_2_O (65:35) to obtain **6** (6.2 mg, t_R_ = 15.0 min). Fr.4-b-7 (102.5 mg) yielded **7** (4.2 mg, t_R_ = 11.7 min) through the HPLC purification using a mobile phase of MeCN–H_2_O (46:54).

Fr.4-c (8.5 g) was subjected to the MPLC system constructed with RP-C18 CC eluted MeCN–H_2_O (30:70–80:20) to obtain 20 products. Compound **9** (1.7 mg) was isolated from Fr.4-c-7 (138.6 mg) by repeated HPLC purification using mobile phases of MeOH–H_2_O (57:43) and MeOH–MeCN–H_2_O (28:28:44). Fr.4-c-10 (512.0 mg) was separated by HPLC eluted with MeCN–H_2_O (30:70) and then purified by HPLC eluted with MeOH–MeCN–H_2_O (19:19:62) and MeOH–H_2_O (60:40) to obtain **1** (0.7 mg, t_R_ = 29.0 min) and **4** (4.3 mg, t_R_ = 16.1 min), respectively. Fr.4-c-11 (1.2 g) was repeatedly purified by RP-HPLC eluted with MeOH–MeCN–H_2_O (15:15:70) and MeOH–H_2_O (70:30) to yield **11** (7.0 mg, t_R_ = 20.9 min). Fr.4-c-12 (1.2 g) was chromatographed on a semi-preparative HPLC to obtain Fr.4-c-12-1–Fr.4-c-12-3. Subsequently, Fr.4-c-12-1 (118.0 mg) was purified by the semi-preparative HPLC eluted with MeOH–MeCN–H_2_O (18:18:62) to produce **2** (2.3 mg, t_R_ = 31.0 min) and **3** (2.5 mg, t_R_ = 32.0 min). Compound **12** (10.0 mg, t_R_ = 30.5 min) was isolated from Fr.4-c-12-1-3 (130.0 mg) by HPLC eluted with MeOH–H_2_O (65:35). Fr.4-c-14 (790.0 mg) was purified by the HPLC eluted with MeCN–H_2_O (43:57) and MeOH–H_2_O (70:30) to obtain **8** (4.9 mg, t_R_ = 25.0 min).

*Boswellianol A (***1***)*. Yellow oil; ${\left[\alpha \right]}_{D}^{25}$ –12.3 (*c* 0.5, MeOH); IR (KBr) *ν*_max_ 2927, 2870, 1693, 1454, 1384, 1076, 1037 cm^–1^; UV (MeOH) *λ*_max_ (log *ε*): 258.9 (1.21), 305.2 (1.57) nm; ECD (MeOH) *λ*_max_ (Δε): 236.0 (1.27), 303.8 (1.57) nm; ^1^H (CDCl_3_, 600 MHz) and ^13^C NMR (CDCl_3_, 150 MHz) data, see Table 1 and Table 3; HR-ESI-MS *m*/*z* 319.2259 [M + H]^+^ (calculated for C_20_H_31_O_3_ as 319.2268).

*Boswellianol B (***2***)*. White amorphous solid; ${\left[\alpha \right]}_{D}^{25}$ –25.7 (*c* 0.5, MeOH); IR (KBr) *ν*_max_ 2962, 2870, 1458, 1437, 1006, 887 cm^–1^; UV (MeOH) *λ*_max_ (log *ε*) 196.8 (2.38) nm; ECD (MeOH) *λ*_max_ (Δε): 207.6 (0.30) nm; ^1^H (CDCl_3_, 600 MHz) and ^13^C NMR (CDCl_3_, 150 MHz) data, see Table 1 and Table 3; HR-ESI-MS *m*/*z* 321.2410 [M + H]^+^ (calculated for C_20_H_33_O_3_ as 321.2424) and 343.2232 [M + Na]^+^ (calculated for C_20_H_32_NaO_3_ as 343.2244).

*Boswellianol C (**3**)*. White amorphous solid; ${\left[\alpha \right]}_{D}^{25}$ –17.0 (*c* 0.5, MeOH); IR (KBr) *ν*_max_ 2927, 2870, 1454, 1373, 964, 887 cm^–1^; UV (MeOH) *λ*_max_ (log *ε*) 195.8 (1.99) nm; ECD (MeOH) *λ*_max_ (Δε): 20.5 (0.30) nm; ^1^H (CDCl_*3*_, 600 MHz) and ^13^C NMR (CDCl_3_, 150 MHz) data, see Table 1 and Table 3; HR-ESI-MS *m*/*z* 321.2411 [M + H]^+^ (calculated for C_20_H_33_O_3_ as 321.2424) and 343.2250 [M + Na]^+^ (calculated for C_20_H_32_NaO_3_ as 343.2244).

*Boswellianol D (***4***)*. Yellow oil; ${\left[\alpha \right]}_{D}^{25}$ –9.5 (*c* 0.5, MeOH); UV (MeOH) *λ*_max_ (log *ε*) 256.1 (1.19) nm, 301.0 (1.66); ECD (MeOH) *λ*_max_ (Δ*ε*): 222.5 (1.33), 294.7 (1.64) nm; ^1^H (CDCl_3_, 600 MHz) and ^13^C NMR (CDCl_3_, 150 MHz) data, see Table 1 and Table 3; HR-ESI-MS *m*/*z* 319.2263 [M + H]^+^ (calculated for C_20_H_31_O_3_ as 319.2268).

*Boswellianol E (***5***)*. Colorless oil; ${\left[\alpha \right]}_{D}^{25}$ –9.8 (*c* 0.5, MeOH); IR (KBr) *ν*_max_ 2966, 2931, 2873, 2364, 2341, 1701, 1458, 1373, 1099, 1055 cm^–1^; UV (MeOH) *λ*_max_ (log *ε*) 195.0 (1.40) nm; ECD (MeOH) *λ*_max_ (Δ*ε*): 199.0 (1.29) nm; ^1^H (CDCl_3_, 600 MHz) and ^13^C NMR (CDCl_3_, 150 MHz) data, see Table 2 and Table 3; HR-ESI-MS *m*/*z* 357.2625 [M + H]^+^ (calculated for C_20_H_37_O_5_ as 357.2636) and 379.2452 [M + Na]^+^ (calculated for C_20_H_36_NaO_5_ as 379.2455).

*Boswellianol F (***6***)*. Colorless oil; ${\left[\alpha \right]}_{D}^{25}$ –9.8 (*c* = 5.0, MeOH); IR (KBr) *ν*_max_ 2966, 2935, 2877, 1708, 1458, 1369, 1328, 1103, 1068 cm^–1^; UV (MeOH) *λ*_max_ (log *ε*) 195.0 (1.55) nm; ECD (MeOH) *λ*_max_ (Δ*ε*): 198.5 (1.48) nm; ^1^H (CDCl_3_, 600 MHz) and ^13^C NMR (CDCl_3_, 150 MHz) data, see Table 2 and Table 3; HR-ESI-MS *m*/*z* 355.2478 [M + H]^+^ (calculated for C_20_H_35_O_5_ as 355.2479) and 377.2304 [M + Na]^+^ (calculated for C_20_H_34_NaO_5_ as 357.2298).

*Boswellianol G (***7***)*. White amorphous solid; ${\left[\alpha \right]}_{D}^{25}$ 2.90 (*c* 0.5, MeOH); IR (KBr) *ν*_max_ 2966, 2935, 1712, 1454, 1242, 1095, 1026 cm^–1^; UV (MeOH) *λ*_max_ (log *ε*) 195.0 (1.48) nm; ECD (MeOH) *λ*_max_ (Δ*ε*): 198.5 (1.55); for ^1^H (CDCl_3_, 600 MHz) and ^13^C NMR (CDCl_3_, 150 MHz) data, see Table 2 and Table 3; HR-ESI-MS *m*/*z* 421.2540 [M + Na]^+^ (calculated for C_22_H_38_NaO_5_ as 421.2561).

*Boswellianol H (***8***)*. Yellow oil; ${\left[\alpha \right]}_{D}^{25}$ 30.48 (*c* 0.5, MeOH); UV (MeOH) *λ*_max_ (log *ε*) 195.0 (1.83) nm; ECD (MeOH) *λ*_max_ (Δ*ε*): 210.3 (2.19) nm; ^1^H (CDCl_3_, 600 MHz) and ^13^C NMR (CDCl_3_, 150 MHz) data, see Table 2 and Table 3; HR-ESI-MS *m*/*z* 307.2637 [M + H]^+^ (calculated for C_20_H_35_O_2_ as 307.2632).

*Boswellianol I (***9***)*. Yellow amorphous solid; ${\left[\alpha \right]}_{D}^{25}$ 9.59 (*c* 0.5, MeOH); IR (KBr) *ν*_max_ 3000, 2935, 2877, 108, 1454, 1369, 1226, 1068, 1029 cm^–1^; UV (MeOH) *λ*_max_ (log *ε*) 195.0 (1.61) nm; ECD (MeOH) *λ*_max_ (Δ*ε*): 203.5 (1.36) nm; ^1^H (CDCl_3_, 600 MHz) and ^13^C NMR (CDCl_3_, 150 MHz) data, see Table 2 and Table 3; HR-ESI-MS *m*/*z* 321.2427 [M + H]^+^ (calculated for C_20_H_33_O_3_ as 321.2424).

### 3.4. Cell Lines and Cultures

The human hepatic stellate cell line LX-2 was provided by the Cell Bank of the Chinese Academy of Sciences (Shanghai, China). The cells were cultured in Dulbecco’s modified Eagle medium (DMEM) with 10% fetal bovine serum (FBS, GIBCO, Grand Island, NY, USA) in a 5% CO_2_ humidified atmosphere at 37.0 °C.

### 3.5. Western Blot Analysis

The LX-2 cells were seeded into a 6-well plate and incubated for 24 h. Cells were pretreated with or without selected compounds (50 µM), SB431542 (2 µM, positive control), or 12.5, 25, and 50 µM of compounds **1** and **5** for 2 h and then stimulated with 5 ng/mL TGF-*β*1 (Sigma-Aldrich, St. Louis, MI, USA) for 45 min (for Phospho-Smad3 and Smad2/3) or 48 h (for FN and N-cad). Cells were collected and lysed in radioimmunoprecipitation (RIPA) buffer with protease inhibitor cocktail (Roche). Total protein was separated by 10% SDS-PAGE Gel and transferred to polyvinylidene difluoride (PVDF) membranes (Millipore, Burlington, MA, USA). Primary antibodies against Smad2/3, N-cad, and GAPDH were purchased from CST, USA, and pSmad3 and FN were obtained from Abclonal, Wuhan, China. Western blots were imaged using an LI-COR Odyssey imaging system (Lincoln, NE, USA).

### 3.6. ECD Calculation

The absolute configuration research was conducted by the Sybyl-X 2.0 software as previously reported [39,40]. All calculations were conducted with an implicit solvent environment described by the conductor-like polarizable continuum model (CPCM). The resulting eight lowest energy conformers for property calculations were determined as stable points on the potential energy surface (PES) without imaginary frequencies (Figure S90). The excitation energies, rotational strengths (velocity), and oscillatory strengths of the initial sixty excited states were calculated by employing the TD-DFT (time-dependent density functional theory) methodology at the PBE0/def2-TZVP level in CPCM methanol. The ECD spectra were simulated by the overlapping Gaussian function (half the bandwidth at 1/e peak height, Sigma = 0.30 for all). To obtain the final spectra, the simulated ECD spectra were averaged based on the Boltzmann distribution theory and their relative Gibbs free energy (∆*G*) to generate the Gaussian curve.

## 4. Conclusions

In conclusion, the phytochemical investigation on the methanolic fraction of olibanum provided a novel prenylmaaliane-type diterpenoid (**1**), three new prenylaromadendrane-type diterpenoids (**2**–**4**), five new highly oxidized cembrane-type diterpenoids (**5**–**9**), and three known diterpenoids (**10**–**12**). Compound **1** possesses an unprecedented diterpenoid skeleton with a 6/6/3 tricyclic system and a prenyl moiety at C-15. Based on the TGF-*β* inhibition screening, compounds **1**, **5**, **9**, and **11** showed TGF-*β* inhibition activities. Furthermore, compounds **1** and **5** dose-dependently suppressed TGF-*β*-induced Smad3 phosphorylation and the upregulation of N-cad and FN in LX-2 cells, which indicated that highly oxidated prenylmaaliane-type and cembrane-type diterpenoids could have stronger TGF-*β* inhibitory activity. These findings give a new insight into the chemical constituents of the polar fraction of olibanum and their inhibitory activities on the TGF-*β*/Smad signaling pathway.

## Figures and Tables

**Table 1:** ^1^H NMR (600 MHz) spectroscopic data of compounds **1**–**4** (*δ* in ppm, CDCl_3_, *J* in Hz).

No.	1	2	3	4
1	3.30, dd, (11.5, 4.4)	2.26, m	2.22, td, (16.0, 6.0)	1.91, overlap
2	1.62, m 1.79, m	1.66, m 1.93, m	1.65, m 1.92, m	1.70, m 1.92, overlap
3	1.53, d, (3.43) 1.84, overlap	1.67, m 1.74, m	1.58, m 1.78, m	1.58, m 1.80, dd
5	1.14, m	1.35, t, (10.7)	1.44, t, (10.9)	1.40, t, (10.5)
6	1.20, overlap	0.61, t, (10.2)	0.80, dd, (11.2, 9.6)	0.98, t, (10.2)
7	1.07, m	0.84, td, (10.3, 6.3)	1.06, q, (5.2)	1.17, dd, (10.1, 3.8)
8	1.70, m 1.93, m	1.03, m 2.03, m	1.07, overlap 2.03, overlap	1.09, m 1.92, overlap
9	0.83, m 1.78, m	2.01, m 2.44, m	2.06, overlap 2.45, q, (6.5)	1.62, m 1.68, m
11	1.31, s	1.28, s	1.22, s	1.23, s
12	0.92, s	4.70, s 4.72, s	4.69, s 4.72, s	1.22, s
14	1.19, s	1.10, s	1.18, s	1.29, s
15	5.80, d, (15.0)	1.44, m 1.57, m	5.36, d, (15.9)	5.79, d, (15.1)
16	6.43, dd, (15.0, 11.1)	4.69, overlap	5.41, d, (15.5, 7.6)	6.45, dd, (15.1, 11.1)
17	6.80, d, (11.1)	5.34, d, (8.0)	3.84, d, (7.6)	6.80, d, (11.1)
19	9.38, s	4.21, d, (12.2) 4.08, d, (12.1)	1.15, s	9.38, s
20	1.84, s	1.82, s	1.19, s	1.84, s

**Table 2:** ^13^C NMR (150 MHz) spectroscopic data of compounds **1**–**9** (*δ* in ppm, CDCl_3_).

No.	1	2	3	4	5	6	7	8	9
1	78.3 CH	50.6 CH	53.2 CH	55.6 CH	88.9 C	87.7 C	89.0 C	49.7 CH	86.4 C
2	25.2 CH_2_	25.2 CH_2_	26.8 CH_2_	23.7 CH_2_	38.9 CH_2_	38.9 CH_2_	35.4 CH_2_	30.7 CH_2_	132.8 CH
3	40.1 CH_2_	41.2 CH_2_	41.8 CH_2_	43.7 CH_2_	72.8 CH	74.4 CH	71.0 CH_2_	125.0 CH	130.1 CH
4	72.1 C	80.7 C	80.9 C	80.2 C	84.8 C	76.7 C	75.1 C	134.5 C	73.3 C
5	47.4 CH	53.1 CH	53.5 CH	47.3 CH	36.9 CH_2_	86.1 CH	42.5 CH_2_	38.9 CH_2_	38.5 CH_2_
6	22.4 CH	25.6 CH	31.0 CH	31.3 CH	27.6 CH_2_	38.7 CH_2_	123.0 CH	24.3 CH_2_	26.6 CH_2_
7	24.0 CH	27.6 CH	28.7 CH	29.5 CH	85.5 CH	75.6 CH	140.2 CH	124.2 CH	82.5 CH
8	15.2 CH_2_	24.8 CH_2_	24.2 CH_2_	19.7 CH_2_	74.7 C	152.5 C	73.7 C	135.2 C	86.3 C
9	36.9 CH_2_	38.7 CH_2_	38.6 CH_2_	41.2 CH_2_	33.9 CH_2_	26.9 CH_2_	23.1 CH_2_	39.2 CH_2_	36.4 CH_2_
10	38.0 C	153.5 C	152.8 C	75.2 C	27.4 CH_2_	32.4 CH_2_	38.8 CH_2_	28.1 CH_2_	28.0 CH_2_
11	23.2 CH_3_	24.5 CH_3_	26.1 CH_3_	24.4 CH_3_	77.3 CH	86.9 CH	78.8 CH	72.9 CH	83.1 CH
12	13.4 CH_3_	106.6 CH_2_	106.9 CH_2_	20.3 CH_3_	85.1 C	85.7 C	84.5 C	75.1 C	86.1 C
13	25.7 C	21.6 C	26.0 C	27.4 C	37.8 CH_2_	29.6 CH_2_	35.4 CH_2_	38.8 CH_2_	30.5 CH_2_
14	11.1 CH_3_	14.8 CH_3_	12.7 CH_3_	12.3 CH_3_	29.9 CH_2_	29.9 CH_2_	32.3 CH_2_	24.8 CH_2_	27.7 CH_2_
15	156.5 CH	50.0 CH_2_	144.9 CH	155.9 CH	32.3 CH	32.3 CH	34.1 CH	74.5 C	36.9 CH
16	120.3 CH	66.3 CH	122.6 CH	120.3 CH	16.6 CH_3_	16.7 CH_3_	17.5 CH_3_	25.9 CH_3_	17.0 CH_3_
17	150.0 CH	131.1 CH	80.2 CH	149.9 CH	18.9 CH_3_	19.3 CH_3_	18.0 CH_3_	29.5 CH_3_	18.4 CH_3_
18	134.8 C	138.7 C	72.8 C	135.0 C	20.3 CH_3_	14.0 CH_3_	23.7 CH_3_	15.6 CH_3_	31.3 CH_3_
19	195.0 CH	62.1 CH_2_	23.7 CH_3_	195.0 CH	29.5 CH_3_	116.2 CH_2_	29.5 CH_3_	17.6 CH_3_	19.0 CH_3_
20	9.4 CH_3_	22.0 CH_3_	26.4 CH_3_	9.4 CH_3_	20.1 CH_3_	25.4 CH_3_	22.4 CH_3_	24.4 CH_3_	25.2 CH_3_
AcO							171.3 C		
							21.2 CH_3_		

**Table 3:** ^1^H NMR (600 MHz) spectroscopic data of compounds **5**–**9** (*δ* in ppm, CDCl_3_, *J* in Hz).

No.	5	6	7	8	9
1				1.50, m	
2	1.56, t, (4.8) 1.99, m	1.54, m 2.20, dd, (15.0, 3.9)	1.66, overlap 1.69, overlap	1.72, overlap 2.12, overlap	5.55, d, (16.3)
3	3.81, d, (7.2)	3.75, dd, (3.3, 2.6)	3.67, dd, (8.5, 2.5)	5.12, dd, (8.3, 4.6)	5.23, d, (16.3)
5	1.73, overlap 2.22, overlap	3.21, d, (10.0)	2.48, ddd, (12.4, 7.2, 1.3) 2.30, dd, (14.4, 6.5)	2.13, overlap 2.25, overlap	1.57, q, (7.7) 1.93, m
6	1.73, overlap 2.28, overlap	1.92, td, (13.6, 10.4) 2.32, dd, (13.7, 5.1)	5.56, td, (15.7, 6.9)	2.13, overlap 2.25, overlap	1.23, m 1.29, m
7	3.90, dd, (10.6, 5.0)	4.30, dd, (10.6, 4.7)	5.77, td, (15.9, 1.4)	5.05, t, (5.34)	3.35, d, (8.9)
9	1.63, m 1.80, m	2.22, m 2.40, m	1.64, overlap 1.74, m	2.16, overlap 2.31, t, (7.3)	1.65, m 1.76, overlap
10	1.85, overlap 2.10, overlap	1.44, m 1.56, m	1.41, m 1.64, overlap	1.47, m 1.79, m	1.64, m 2.21, dd, (3.8, 1.4)
11	4.25, dd, (11.4, 2.7)	3.27, dd, (11.4, 2.6)	4.88, dd, (11.0, 2.2)	3.96, t, (7.4)	4.12, dd, (9.5, 3.0)
13	1.84, overlap 2.12, overlap	1.37, (12.2, 7.9) 2.09, td, (12.4, 7.6)	1.66, overlap 1.78, m	1.65, m 1.73, m	1.44, m 1.85, m
14	1.44, dd, (11.4, 2.0) 2.16, dd, (13.9, 7.1)	1.31, (11.9, 7.6) 1.77, td, (12.3, 8.0)	1.65, overlap 1.97, m	2.00, m 2.06, m	1.77, overlap 1.97, m
15	2.15, t, (6.2)	2.02, t, (7.0)	2.06, overlap	-	1.79, overlap
16	0.94, d, (6.8)	0.94, d, (6.9)	0.91, d, (6.8)	1.19, s	0.88, d, (6.9)
17	0.90, d, (6.8)	0.95, d, (6.9)	0.86, d, (6.8)	1.26, s	0.83, d, (6.9)
18	1.18, s	1.16, s	1.28, s	1.55, s	1.31, s
19	1.16, s	5.05, s 5.30, s	1.30, s	1.64, s	1.08, s
20	1.12, s	1.02, s	1.17, s	1.18, s	1.07, s
AcO			2.07, s		

## Data Availability

Data on the compounds are available in the Supplementary Materials.

## Supplementary Materials

The following supporting information can be downloaded at: https://www.mdpi.com/article/10.3390/plants13081074/s1; Figures S1–S90: HR-ESI-MS spectra, IR, ECD, UV-Vis, and 1D and 2D NMR spectra of compounds **1**–**9**, TGF-*β* inhibition screening and DFT computational optimized conformation of compound **1**; Tables S1 and S2: Detailed quantum chemical ECD computational data of compound **1**.

## References

[1] POWO Plants of the World Online. https://powo.science.kew.org/taxon/urn:lsid:ipni.org:names:5117-1.

[2] Ojha P.K., Poudel D.K., Rokaya A., Satyal R., Setzer W.N., Satyal P. (2022). Comparison of Volatile Constituents Present in Commercial and Lab-Distilled Frankincense (*Boswellia carteri*) Essential Oils for Authentication. Plants.

[3] Cao B., Wei X.-C., Xu X.-R., Zhang H.-Z., Luo C.-H., Feng B., Xu R.-C., Zhao S.-Y., Du X.-J., Han L. (2019). Seeing the Unseen of the Combination of Two Natural Resins, Frankincense and Myrrh: Changes in Chemical Constituents and Pharmacological Activities. Molecules.

[4] Gadisa E., Weldearegay G., Desta K., Tsegaye G., Hailu S., Jote K., Takele A. (2019). Combined antibacterial effect of essential oils from three most commonly used Ethiopian traditional medicinal plants on multidrug resistant bacteria. BMC Complement. Altern. Med..

[5] Elakkiya V., Krishnan K., Bhattacharyya A., Selvakumar R. (2020). Advances in Ayurvedic medicinal plants and nanocarriers for arthritis treatment and management: A review. J. Her. Med..

[6] Abolhasanzadeh Z., Ashrafi H., Badr P., Azadi A. (2017). Traditional neurotherapeutics approach intended for direct nose to brain delivery. J. Ethnopharmacol..

[7] Eltahir H.M., Fawzy M.A., Mohamed E.M., Alrehany M.A., Shehata A.M., Abouzied M.M. (2020). Antioxidant, anti-inflammatory and anti-fibrotic effects of *Boswellia serrate* gum resin in CCl_4_-induced hepatotoxicity. Exp. Ther. Med..

[8] Latella G., Sferra R., Vetuschi A., Zanninelli G., D’Angelo A., Catitti V., Caprilli R., Gaudio E. (2008). Prevention of colonic fibrosis by *Boswellia* and *Scutellaria* extracts in rats with colitis induced by 2,4,5-trinitrobenzene sulphonic acid. Eur. J. Clin. Investig..

[9] Sferra R., Vetuschi A., Catitti V., Ammanniti S., Pompili S., Melideo D., Frieri G., Gaudio E., Latella G. (2012). *Boswellia serrata* and *Salvia miltiorrhiza* extracts reduce DMN-induced hepatic fibrosis in mice by TGF-*β*1 downregulation. Eur. Rev. Med. Pharmacol. Sci..

[10] Shen Y., Takahashi M., Byun H.-M., Link A., Sharma N., Balaguer F., Leung H.-C., Boland C.R., Goel A. (2012). Boswellic acid induces epigenetic alterations by modulating DNA methylation in colorectal cancer cells. Cancer Biol. Ther..

[11] Vuddanda P.R., Singh S., Velaga S. (2016). Boswellic acid—Medicinal use of an ancient herbal remedy. J. Her. Med..

[12] Liu M., Chen P., Büchele B., Dong S., Huang D., Ren C., Zhang Y., Hou X., Simmet T., Shen J. (2013). A boswellic acid-containing extract attenuates hepatic granuloma in C57BL/6 mice infected with *Schistosoma japonicum*. Parasitol. Res..

[13] Liu M., Liu T., Shang P., Zhang Y., Liu L., Liu T., Sun S. (2018). Acetyl-11-keto-*β*-boswellic acid ameliorates renal interstitial fibrosis via Klotho/TGF-*β*/Smad signalling pathway. J. Cell. Mol. Med..

[14] Zhang Y., Ning Z., Lu C., Zhao S., Wang J., Liu B., Xu X., Liu Y. (2013). Triterpenoid resinous metabolites from the genus Boswellia: Pharmacological activities and potential species-identifying properties. Chem. Cent. J..

[15] Al-Harrasi A., Avula S.K., Csuk R., Das B. (2021). Cembranoids from *Boswellia* species. Phytochemistry.

[16] Wang Y.-G., Ren J., Wang A.-G., Yang J.-B., Ji T.-F., Ma Q.-G., Tian J., Su Y.-L. (2013). Hepatoprotective Prenylaromadendrane-Type Diterpenes from the Gum Resin of *Boswellia carterii*. J. Nat. Prod..

[17] Yu J., Geng Y., Zhao H., Wang X. (2018). Diterpenoids from the gum resin of *Boswellia carterii* and their biological activities. Tetrahedron.

[18] Sun X., Geng Y., Wang X., Qin D., Yu J. (2020). Cembrane-type diterpenoids from the gum resin of *Boswellia carterii* and their biological activities. RSC Adv..

[19] Wang Y.-g., Ren J., Ma J., Yang J.-b., Ji T., Wang A.-g. (2019). Bioactive cembrane-type diterpenoids from the gum-resin of *Boswellia carterii*. Fitoterapia.

[20] Guo F., Zhao L., Zhang K., Wang X., Yu J. (2021). Anti-inflammatory and hepatoprotective cembranes from the gum resin of *Boswellia carterii*. Phytochem. Lett..

[21] Pollastro F., Golin S., Chianese G., Putra M.Y., Schiano Moriello A., De Petrocellis L., García V., Munoz E., Taglialatela-Scafati O., Appendino G. (2016). Neuroactive and Anti-inflammatory Frankincense Cembranes: A Structure–Activity Study. J. Nat. Prod..

[22] Wang J.-J., Suo X.-Y., Sun H.-R., Wang X., Lin M.-B., Wang J.-H., Jiang J.-D., Ji T.-F. (2022). Prenylaromadendrane-type diterpenoids from the gum resin of *Boswellia sacra* flueck and their cytotoxic effects. Nat. Prod. Res..

[23] Yuan Z., Liu D., Zhang B., Cao S., Yao T., Zhao Q., Qiu F., Zhao F. (2023). New verticillane-diterpenoid as potent NF-κB inhibitor isolated from the gum resin of *Boswellia sacra*. Fitoterapia.

[24] Chen F.L., Liu D.L., Fu J., Yang J., Bai L.P., Zhang W., Jiang Z.H., Zhu G.Y. (2022). (±)-Atrachinenins A–C, Three Pairs of Caged C27 Meroterpenoids from the Rhizomes of *Atractylodes chinensis*. Chin. J. Chem..

[25] Liu X., Fu J., Shen R.-S., Wu X.-J., Yang J., Bai L.-P., Jiang Z.-H., Zhu G.-Y. (2021). Linderanoids A–O, dimeric sesquiterpenoids from the roots of *Lindera aggregata* (Sims) Kosterm. Phytochemistry.

[26] Wu X.-J., Cao D., Chen F.-L., Shen R.-S., Gao J., Bai L.-P., Zhang W., Jiang Z.-H., Zhu G.-Y. (2022). Chlorfortunones A and B, Two Sesquiterpenoid Dimers, Possessing Dispiro[4,2,5,2]pentadecane-6,10,14-tren Moiety from *Chloranthus fortunei*. ACS Omega.

[27] Ren J., Wang Y.-G., Wang A.-G., Wu L.-Q., Zhang H.-J., Wang W.-J., Su Y.-L., Qin H.-L. (2015). Cembranoids from the Gum Resin of *Boswellia carterii* as Potential Antiulcerative Colitis Agents. J. Nat. Prod..

[28] Brochini C.B., Roque N.F. (2000). Two new cneorubin related diterpenes from the leaves of *Guarea guidonia* (*Meliaceae*). J. Braz. Chem. Soc..

[29] Kitajima J., Kimizuka K., Tanak Y. (2000). Three new sesquiterpenoid glucosides of Ficus pumila fruit. Chem. Pharm. Bull..

[30] Feng Y., Zhang Q., Sun L. (2021). Five terpenoids from the gum resin of *Boswellia carterii* and their cytotoxicity. Fitoterapia.

[31] Xu B., Li Z., Alsup T.A., Ehrenberger M.A., Rudolf J.D. (2021). Bacterial diterpene synthases prenylate small molecules. ACS Catal..

[32] Li H., Dickschat J.S. (2022). Diterpene Biosynthesis from Geranylgeranyl Diphosphate Analogues with Changed Reactivities Expands Skeletal Diversity. Angew. Chem. Int. Ed..

[33] Durán-Peña M.J., Botubol Ares J.M., Hanson J.R., Collado I.G., Hernández-Galán R. (2015). Biological activity of natural sesquiterpenoids containing a gem-dimethylcyclopropane unit. Nat. Prod. Rep..

[34] Hackl T., König W.A., Muhle H. (2004). Isogermacrene A, a proposed intermediate in sesquiterpene biosynthesis. Phytochemistry.

[35] Rahelivao M.P., Gruner M., Lübken T., Islamov D., Kataeva O., Andriamanantoanina H., Bauer I., Knölker H.-J. (2016). Chemical constituents of the soft corals *Sinularia vanderlandi* and *Sinularia gravis* from the coast of Madagascar. Org. Biomol. Chem..

[36] Carmely S., Groweiss A., Kashman Y. (1981). Decaryiol, a new cembrane diterpene from the marine soft coral *Sarcophyton decaryi*. J. Org. Chem..

[37] Dhar D., Baglieri J., Kisseleva T., Brenner D.A. (2020). Mechanisms of liver fibrosis and its role in liver cancer. Exp. Biol. Med..

[38] Kisseleva T., Brenner D. (2021). Molecular and cellular mechanisms of liver fibrosis and its regression. Nat. Rev. Gastroenterol. Hepatol..

[39] Chen F.-L., Liu D.-L., Fu J., Fu L., Gao J., Bai L.-P., Zhang W., Jiang Z.-H., Zhu G.-Y. (2022). Atrachinenynes A–D, four diacetylenic derivatives with unprecedented skeletons from the rhizomes of *Atractylodes chinensis*. New J. Chem..

[40] Ren W.-J., Io C.-C., Jiang R., Ng K.-F., Liu J.-Z., Bai L.-P., Zhang W., Jiang Z.-H., Liu Y.-H., Zhu G.-Y. (2023). Di- and Triterpenoids from the Rhizomes of *Isodon amethystoides* and Their Anti-inflammatory Activities. J. Nat. Prod..

