# Supplementary material for: Boswellianols A–I, Structurally Diverse Diterpenoids from the Oleo-Gum Resin of Boswellia carterii and Their TGF-β Inhibition Activity
Source: Plants (Basel). 2024 Apr 11;13(8):1074. doi: 10.3390/plants13081074 (PMC11054202; doi:10.3390/plants13081074)
Supplement: Supplementary file 1 [file plants-13-01074-s001.zip › plants-2926063-supplementary.pdf]

Supplementary Materials

Boswellianols A–I, Structurally Diverse Diterpenoids from the Oleo-gum Resin of *Boswellia carterii* and Their TGF-*β* Inhibition Activity

Zhi-Rong Lin [1], Meng-Yu Bao [1], Hao-Ming Xiong [1], Dai Cao [1], Li-Ping Bai [1], Wei Zhang [1], Cheng-Yu Chen [2], Zhi-Hong Jiang [1*], and Guo-Yuan Zhu [1*]

[1] State Key Laboratory of Quality Research in Chinese Medicines, Guangdong-Hong Kong-Macao Joint Laboratory of Respiratory Infectious Disease, Macau Institute for Applied Research in Medicine and Health, Macau University of Science and Technology, Macau 999078, China; thomas05@sina.cn (Z.-R.L.); 3220004965@student.must.edu.mo (M.-Y.B.); 3230006692@ student.must.edu.mo (H.-M.X.); caodai8989@163.com (D.C.); lpbai@must.edu.mo (L.-P.B.); wzhang@must.edu.mo (W. Z.)

[2] Jiaheng Pharmaceutical Technology Co., Ltd., Zhuhai 519000, China; yanfachenchengyu@fusenpharma.com

* Correspondence: zhjiang@must.edu.mo (Z.-H. Jiang), gyzhu@must.edu.mo (G.-Y. Zhu).

The List of Contents

FigureS1. HRESIMS spectrum of compound 1..1
FigureS2. ^{1}H NMR spectrum of compound 1..1
FigureS3. ^{13}C-NMR spectrum of compound 1..2
FigureS4. HSQC NMR spectrum of compound 1..2
FigureS5. HMQC NMR spectrum of compound 1..3
FigureS6. ^{1}H-^{1}H COSY spectrum of compound 1..3
FigureS7. NOESY spectrum of compound 1..4
FigureS8. The UV spectrum of compound 1..4
FigureS9. The IR spectrum of compound 1..5
FigureS10. The ECD spectrum of compound 1..5
FigureS11. HRESIMS spectrum of compound 2..6
FigureS12. ^{1}H NMR spectrum of compound 2..6
FigureS13. ^{13}C NMR spectrum of compound 2..7
FigureS14. HSQC NMR spectrum of compound 2..7
FigureS15. HMQC NMR spectrum of compound 2..8
FigureS16. ^{1}H-^{1}H COSY spectrum of compound 2..8
FigureS17. NOESY NMR spectrum of compound 2..9
FigureS18. UV spectrum of compound 2. 9
FigureS19. IR spectrum of compound 2. 10
FigureS20. ECD spectrum of compound 2. 10
FigureS21. HRESIMS spectrum of compound 3..11
FigureS22. ^{1}H NMR spectrum of compound 3..11
FigureS23. ^{13}C NMR spectrum of compound 3..12
FigureS24. HSQC NMR spectrum of compound 3..12
FigureS25. HMBC NMR spectrum of compound 3..13
FigureS26. ^{1}H-^{1}H COSY NMR spectrum of compound 3..13
FigureS27. NOESY NMR spectrum of compound 3..14
FigureS28. UV spectrum of compound 3..14
FigureS29. IR spectrum of compound 3. 15
FigureS30. ECD spectrum of compound 3..15
FigureS31. HRESIMS spectrum of compound 4..16
FigureS32. ^{1}H NMR spectrum of compound 4..16
FigureS33. ^{13}C-NMR spectrum of Compound 4..17
FigureS34. HSQC NMR spectrum of compound 4..17
FigureS35. HMBC NMR spectrum of compound 4..18
FigureS36. ^{1}H-^{1}H COSY NMR spectrum of compound 4..18
FigureS37. NOESY NMR spectrum of compound 4..19
FigureS38. UV spectrum of compound 4..19
FigureS39. ECD spectrum of compound 4..20
FigureS40. HRESIMS spectrum of compound 5..21
FigureS41. ^{1}H NMR spectrum of compound 5..21

FigureS42. ^{13}C NMR spectrum of compound 5....22
FigureS43. HSQC NMR spectrum of compound 5....22
FigureS44. HMBC NMR spectrum of compound 5....23
FigureS45. ^{1}H-^{1}H COSY NMR spectrum of compound 5....23
FigureS46. NOESY NMR spectrum of compound 5....24
FigureS47. UV spectrum of compound 5....24
FigureS48. IR spectrum of compound 5....25
FigureS49. ECD spectrum of compound 5....25
FigureS50. HRESIMS spectrum of compound 6....26
FigureS51. ^{1}H NMR spectrum of compound 6....26
FigureS52. ^{13}C NMR spectrum of compound 6....27
FigureS53. HSQC NMR spectrum of compound 6....27
FigureS54. HMBC NMR spectrum of compound 6....28
FigureS55. ^{1}H-^{1}H COSY NMR spectrum of compound 6....28
FigureS56. NOESY NMR spectrum of compound 6....29
FigureS57. UV spectrum of compound 6....29
FigureS58. IR spectrum of compound 6....30
FigureS59. ECD spectrum of compound 6....30
FigureS60. HRESIMS spectrum of compound 7....31
FigureS61. ^{1}H NMR spectrum of compound 7....31
FigureS62. ^{13}C NMR spectrum of compound 7....32
FigureS63. HSQC NMR spectrum of compound 7....32
FigureS64. HMBC NMR spectrum of compound 7....33
FigureS65. ^{1}H-^{1}H COSY NMR spectrum of compound 7....33
FigureS66. NOESY NMR spectrum of compound 7....34
FigureS67. UV spectrum of compound 7....34
FigureS68. IR spectrum of compound 7....35
FigureS69. ECD spectrum of compound 7....35
FigureS70. HRESIMS spectrum of compound 8....36
FigureS71. ^{1}H NMR spectrum of compound 8....36
FigureS72. ^{13}C NMR spectrum of Compound 8....37
FigureS73. HSQC NMR spectrum of compound 8....37
FigureS74. HMBC NMR spectrum of compound 8....38
FigureS75. ^{1}H-^{1}H COSY NMR spectrum of compound 8....38
FigureS76. NOESY NMR spectrum of compound 8....39
FigureS77. UV spectrum of compound 8....39
FigureS78. ECD spectrum of compound 8....40
FigureS79. HRESIMS spectrum of compound 9....41
FigureS80. ^{1}H NMR spectrum of compound 9....41
FigureS81. ^{13}C NMR spectrum of compound 9....42
FigureS82. HSQC NMR spectrum of compound 9....42
FigureS83. HMBC NMR spectrum of compound 9....43
FigureS84. ^{1}H-^{1}H COSY NMR spectrum of compound 9....43
FigureS85. NOESY NMR spectrum of compound 9....44

FigureS86. UV spectrum of compound 9 44
FigureS87. IR spectrum of compound 9 45
FigureS88. ECD spectrum of compound 9 45
FigureS89. Western blot of TGF-β/Smad signal molecules for screening isolated compounds 1, 5, 7, 9, 10, and 11 at 25μM. GADPH was used as an invariant control for equal loading.............46
FigureS90. DFT computational optimized conformations of (1*R*,4*R*,5*R*,6*S*,7*S*,10*R*,13*R*)-1. 46
TableS1. Gibbs free energies[a] and equilibrium populations[b] of low-energy conformers of compound 1. 46
TableS2. Cartesian coordinates for the low-energy reoptimized random research conformers of compound 1 at B3LYP-D3(BJ)/6-31G* level of theory in methanol. 47

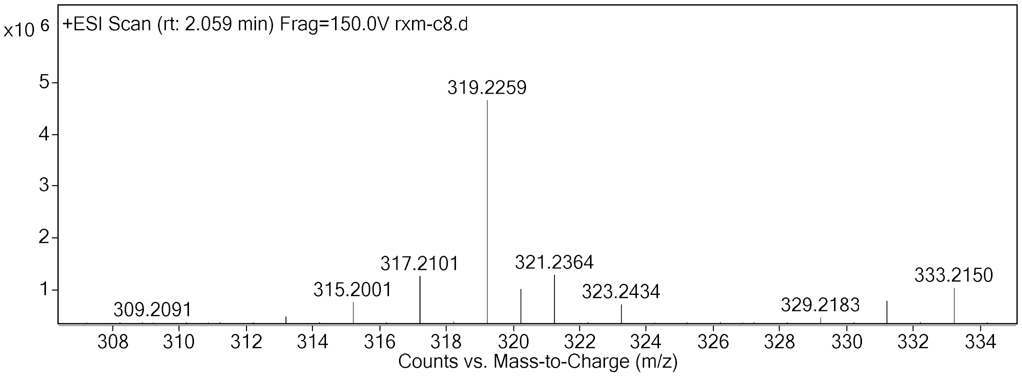

Formular	Score	Mass	Mass (MGF)	*m/z* (Calc)	Diff (ppm)	Ion Formular	*m/z*
$C_{20}H_{30}O_3$	99.84	318.2191	319.2268	319.2268	1.17	$C_{20}H_{31}O_3$	319.2259

FigureS1. HRESIMS spectrum of compound **1**.

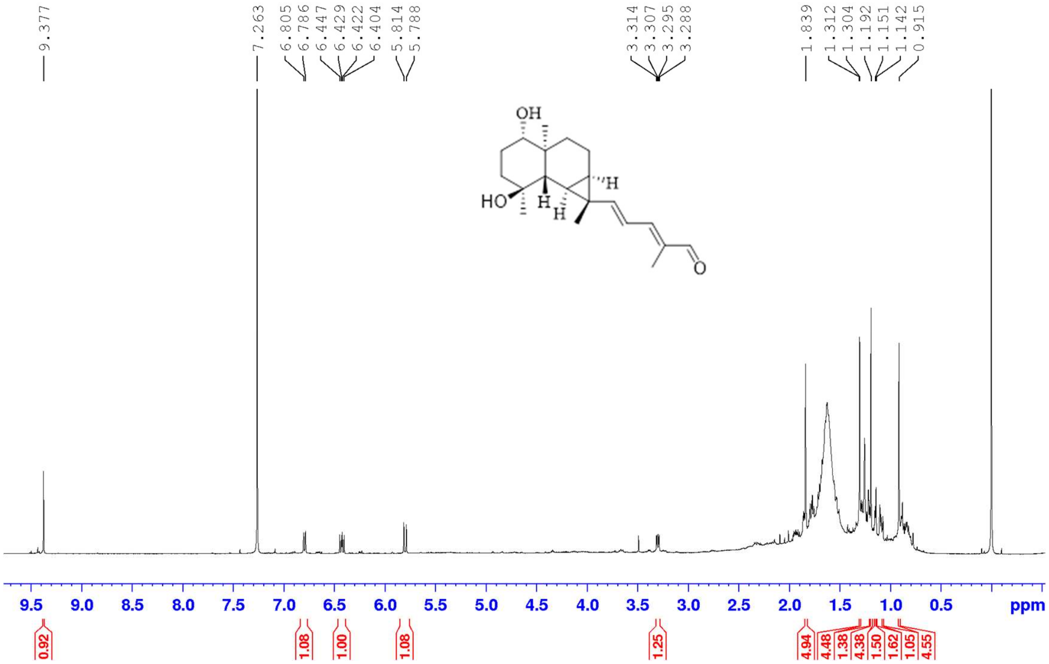

FigureS2.^{1}H NMR spectrum of compound **1**

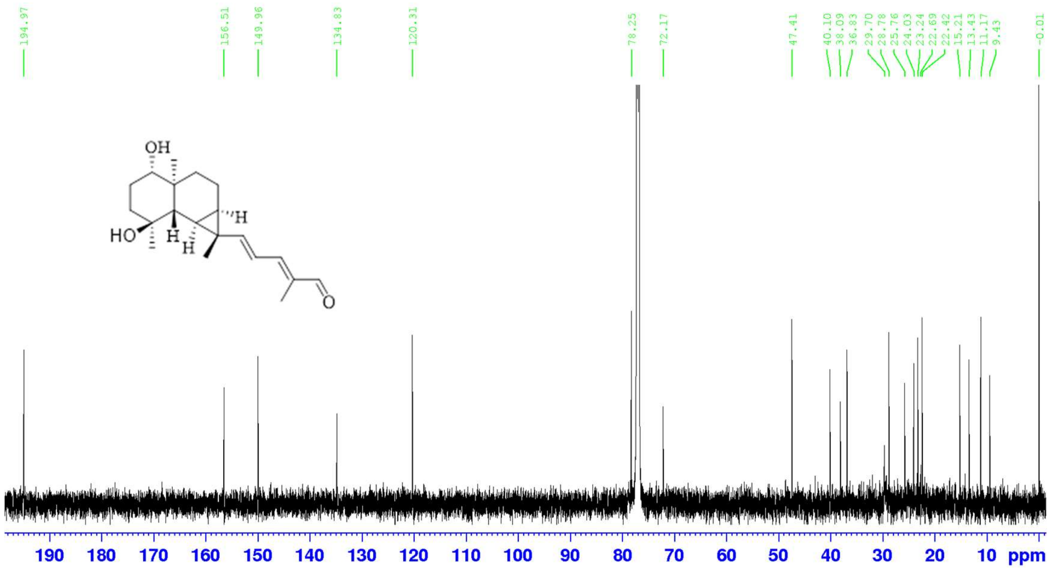

FigureS3.^{13}C-NMR spectrum of compound **1**

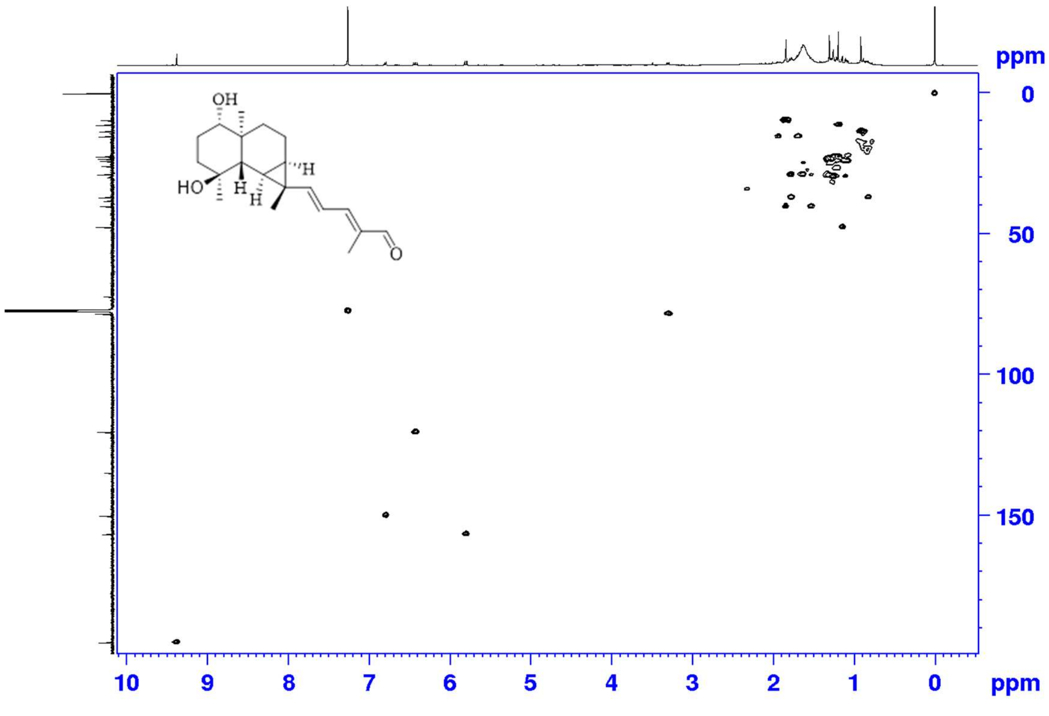

FigureS4.HSQC NMR spectrum of compound **1**

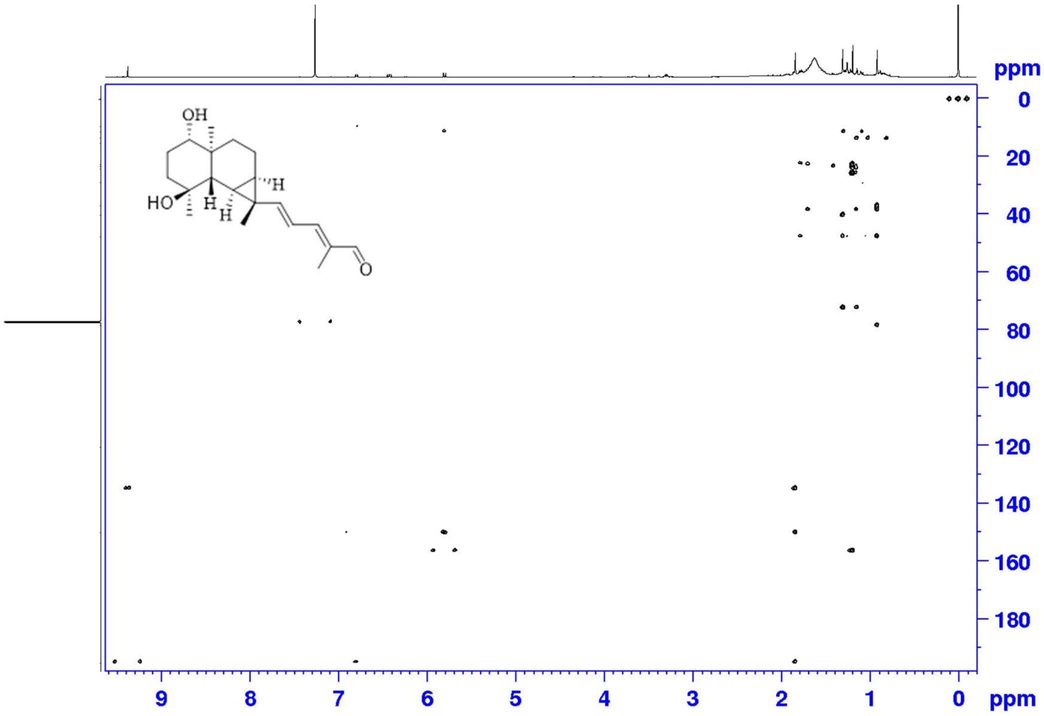

FigureS5.HMQC NMR spectrum of compound 1

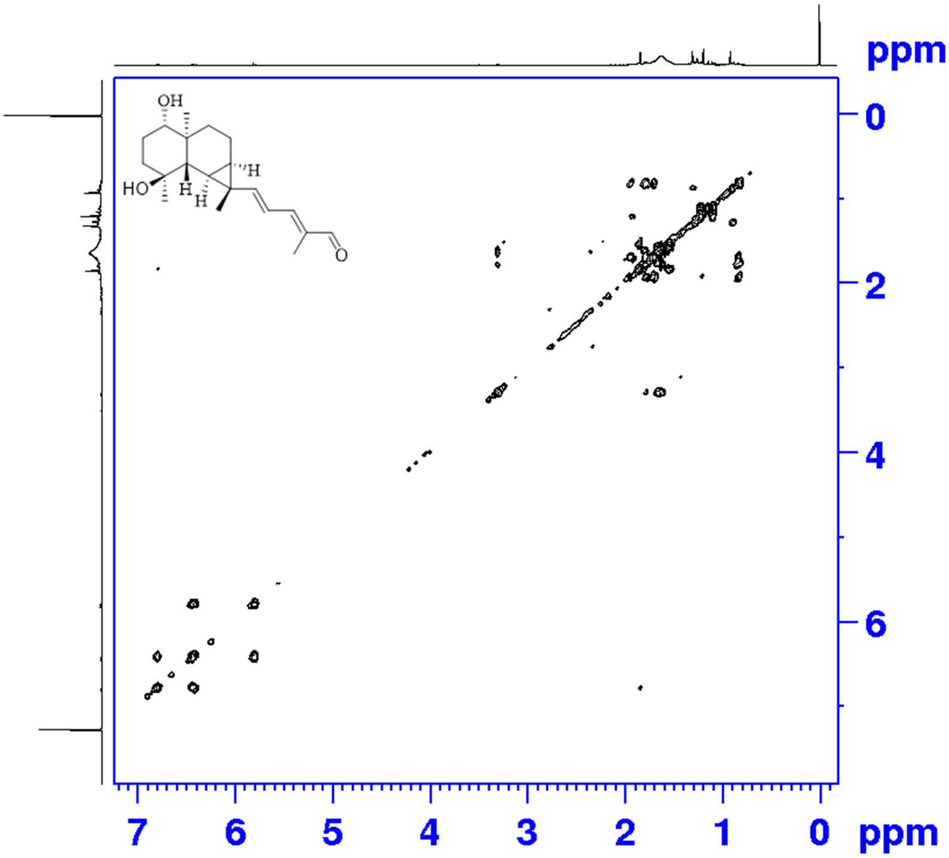

FigureS6.^{1}H-^{1}H COSY spectrum of compound **1**

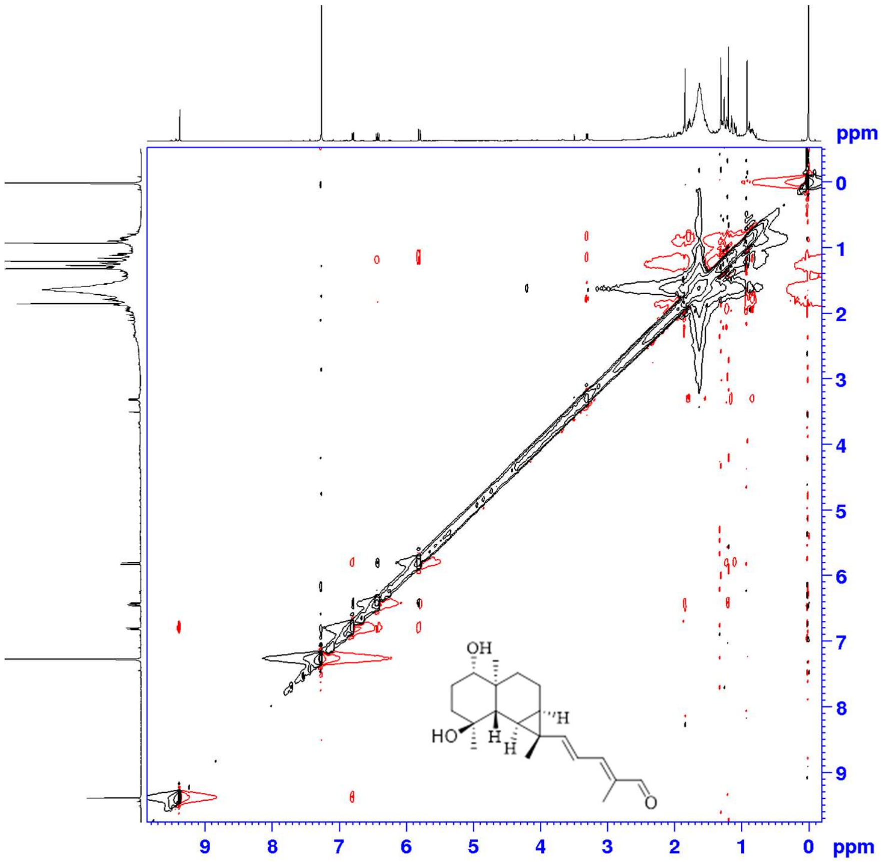

FigureS7.NOESY spectrum of compound **1**

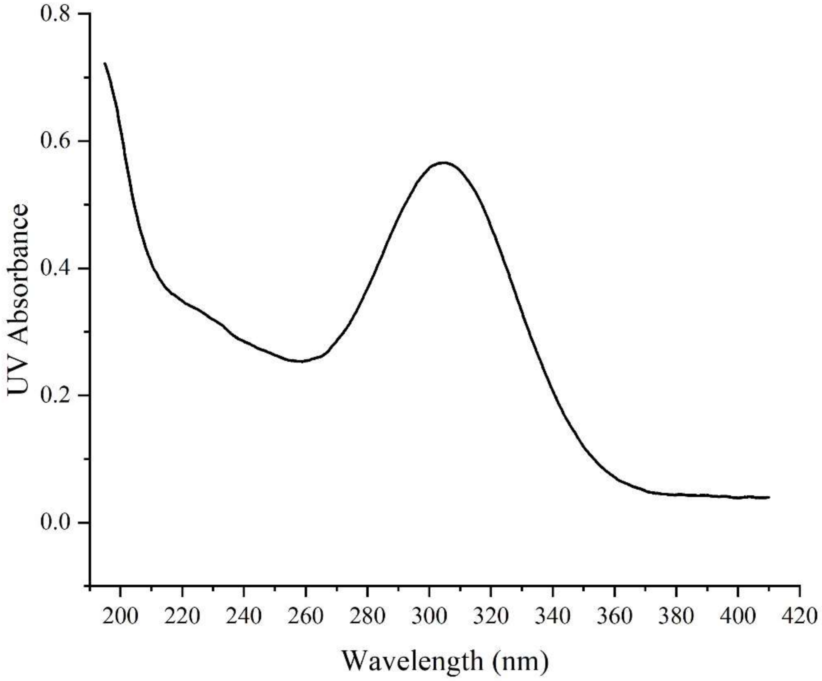

FigureS8. The UV spectrum of compound **1**

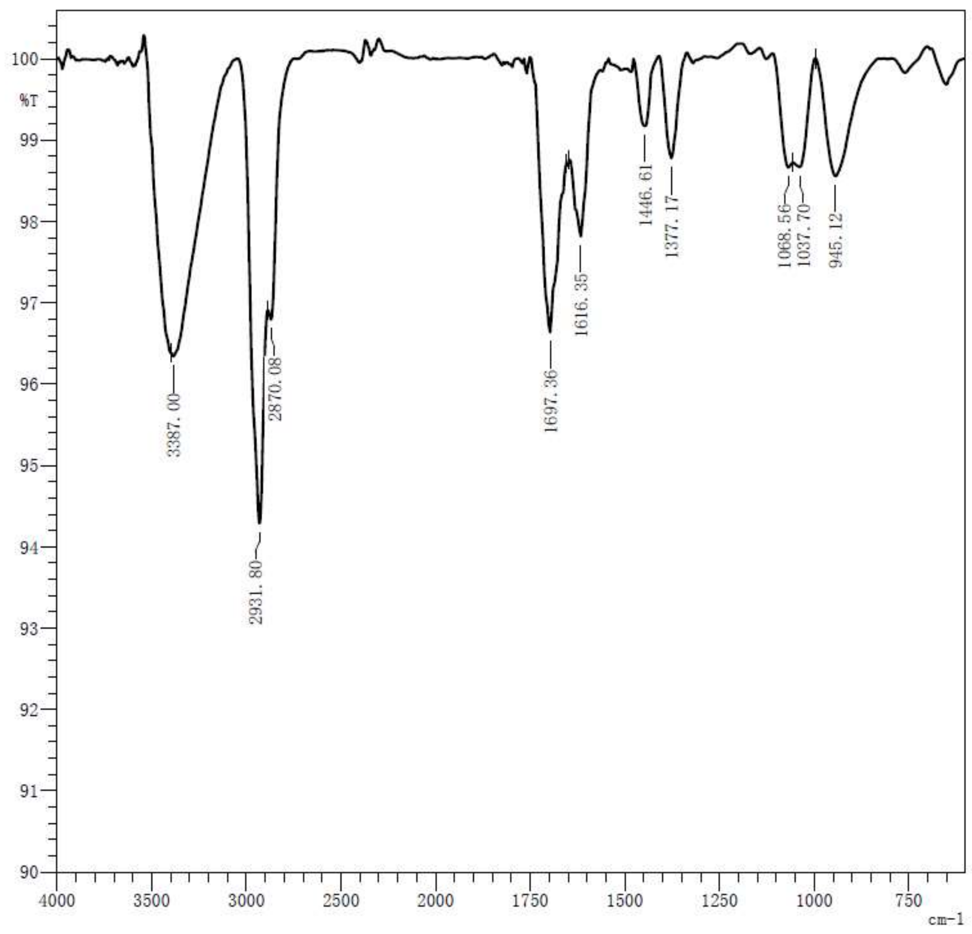

FigureS9. The IR spectrum of compound **1**.

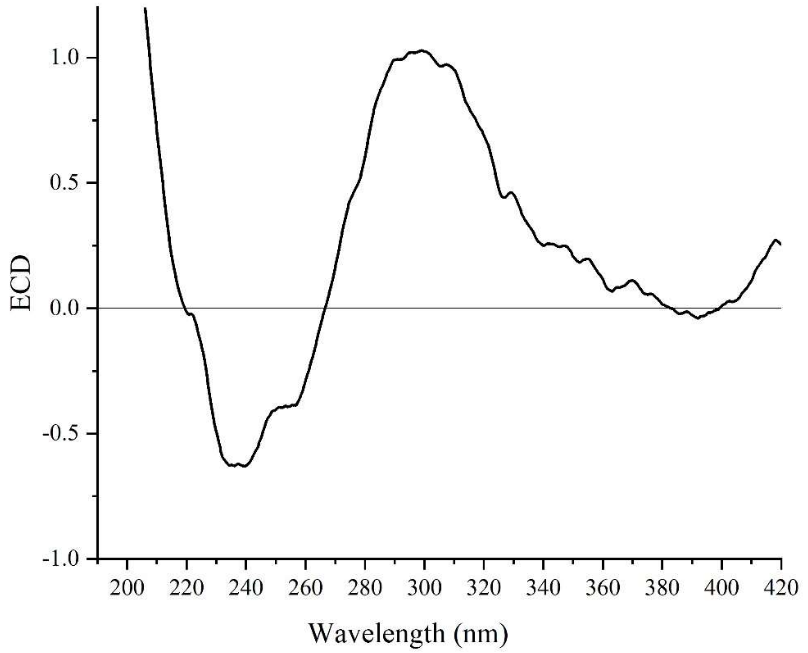

FigureS10. The ECD spectrum of compound **1**

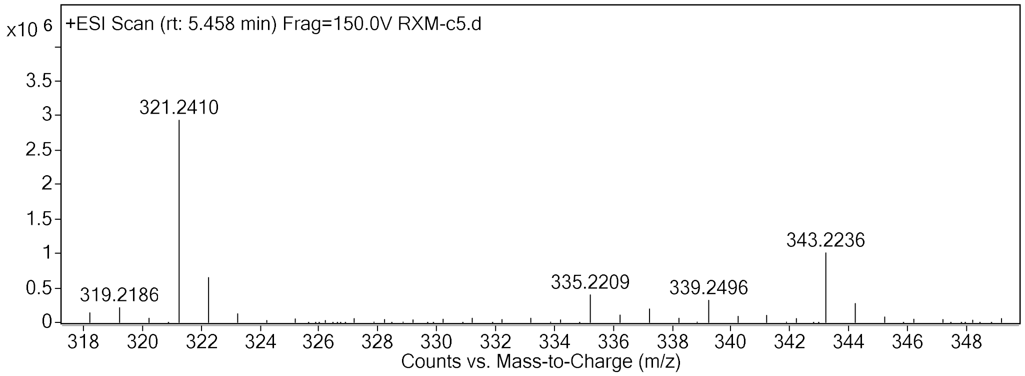

Formular	Score	Mass	Mass (MGF)	*m/z* (Calc)	Diff (ppm)	Ion Formular	*m/z*
$C_{20}H_{32}O_3$	97.75	320.2340	320.2337	321.2424	4.44	$C_{20}H_{33}O_3$	321.2410
$C_{20}H_{32}O_3$	99.33	320.2340	320.2351	343.2244	3.64	$C_{20}H_{32}NaO_3$	343.2236

FigureS11. HRESIMS spectrum of compound **2**

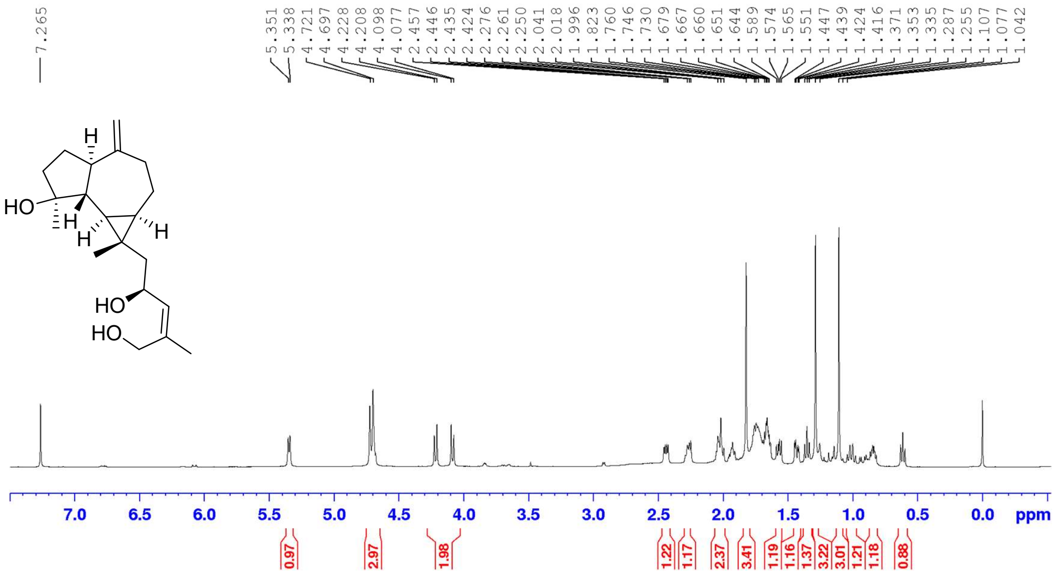

FigureS12. ^{1}H NMR spectrum of compound **2**.

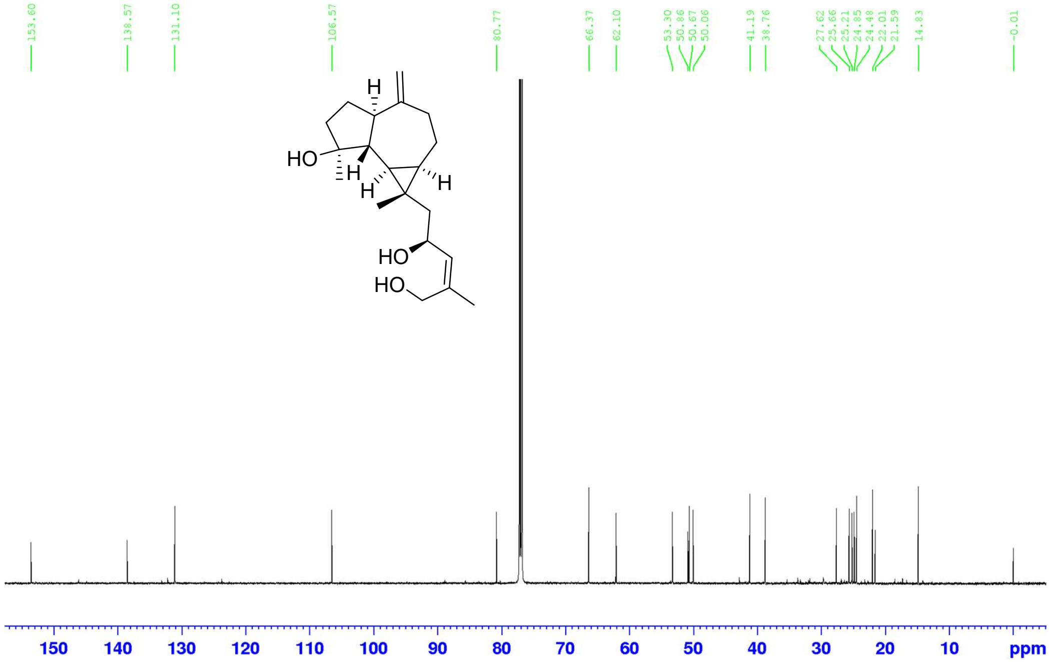

FigureS13. ^{13}C NMR spectrum of compound **2**

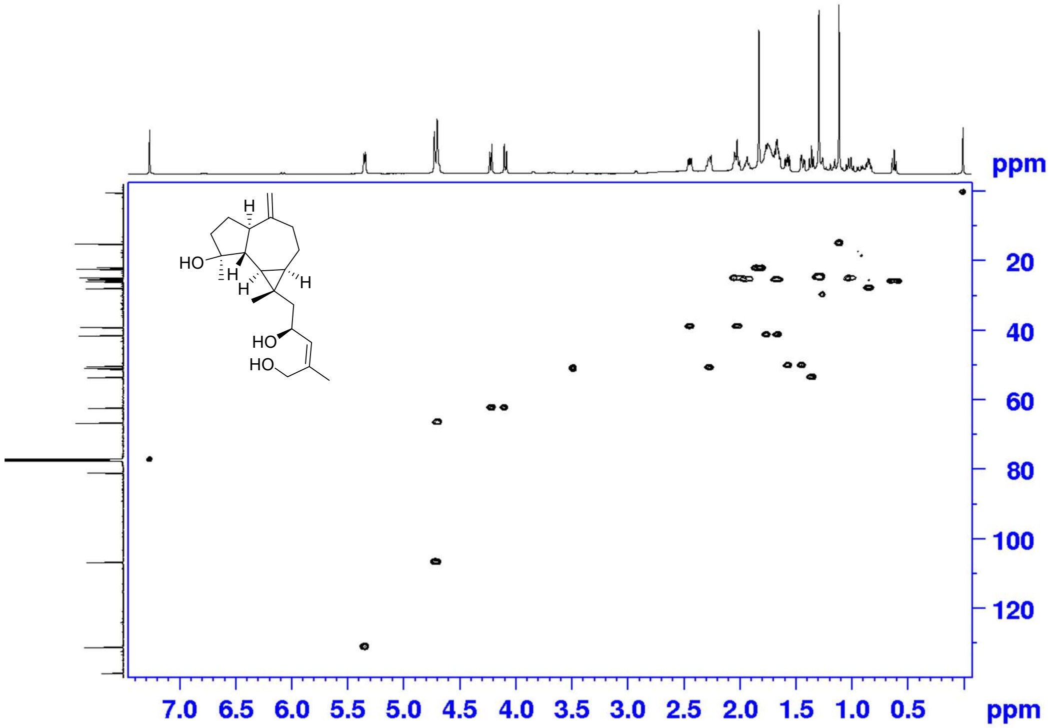

FigureS14. HSQC NMR spectrum of compound **2**

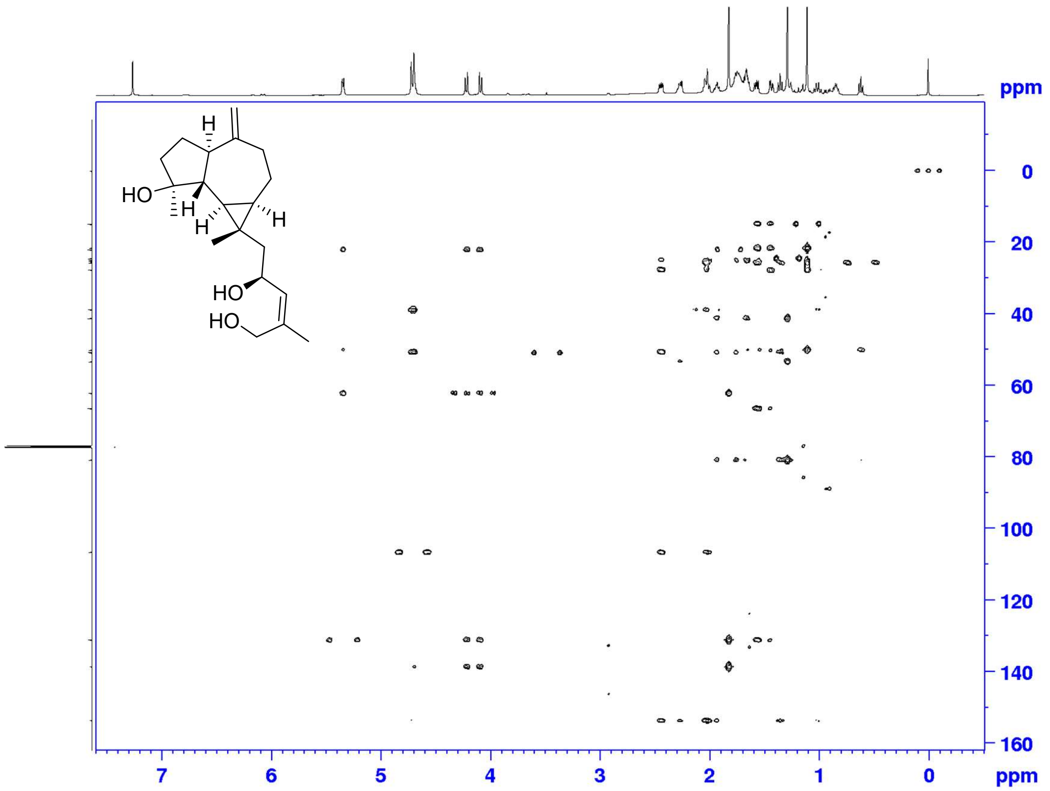

FigureS15. HMQC NMR spectrum of compound **2**

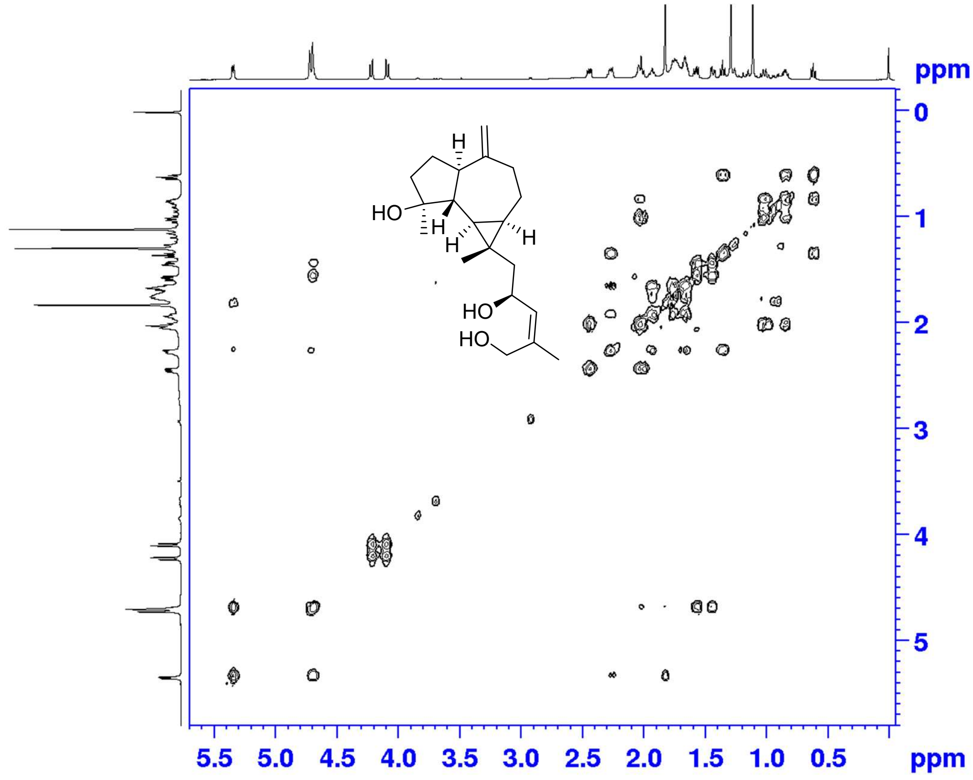

FigureS16. ^{1}H-^{1}H COSY spectrum of compound **2**

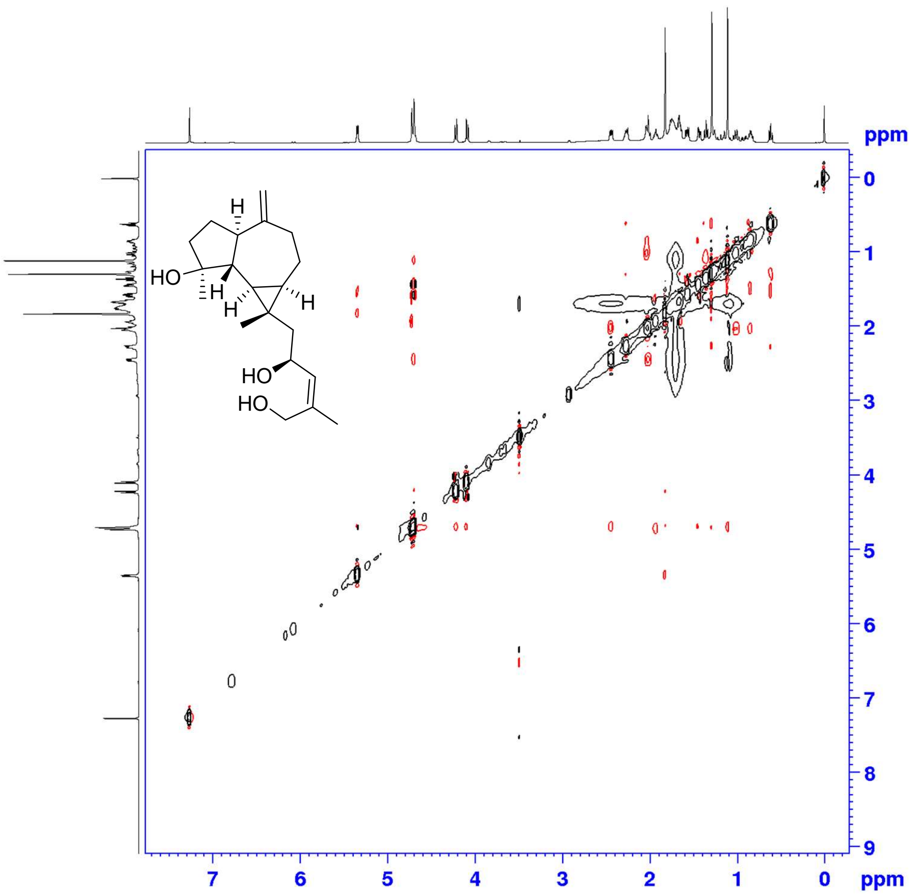

FigureS17. NOESY NMR spectrum of compound **2**

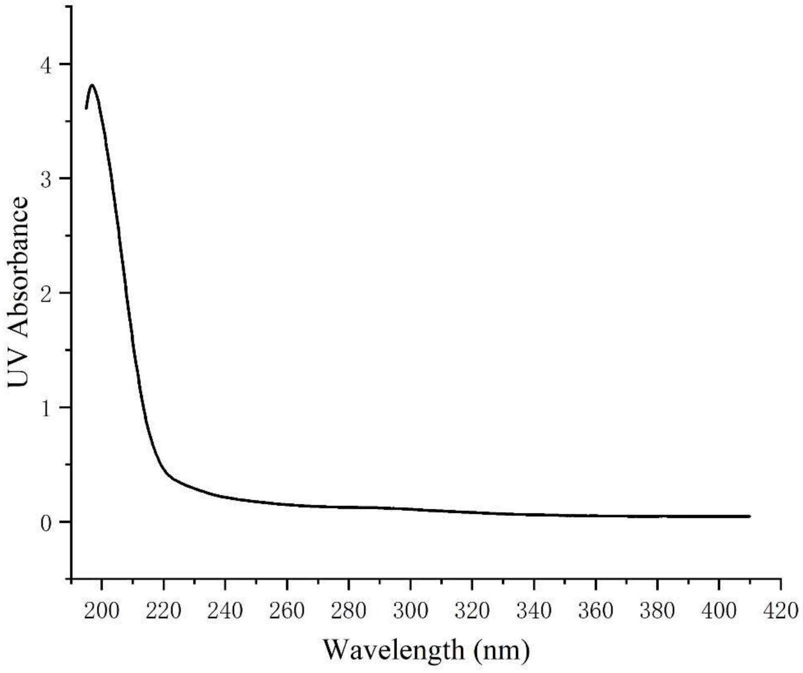

FigureS18. UV spectrum of compound **2**.

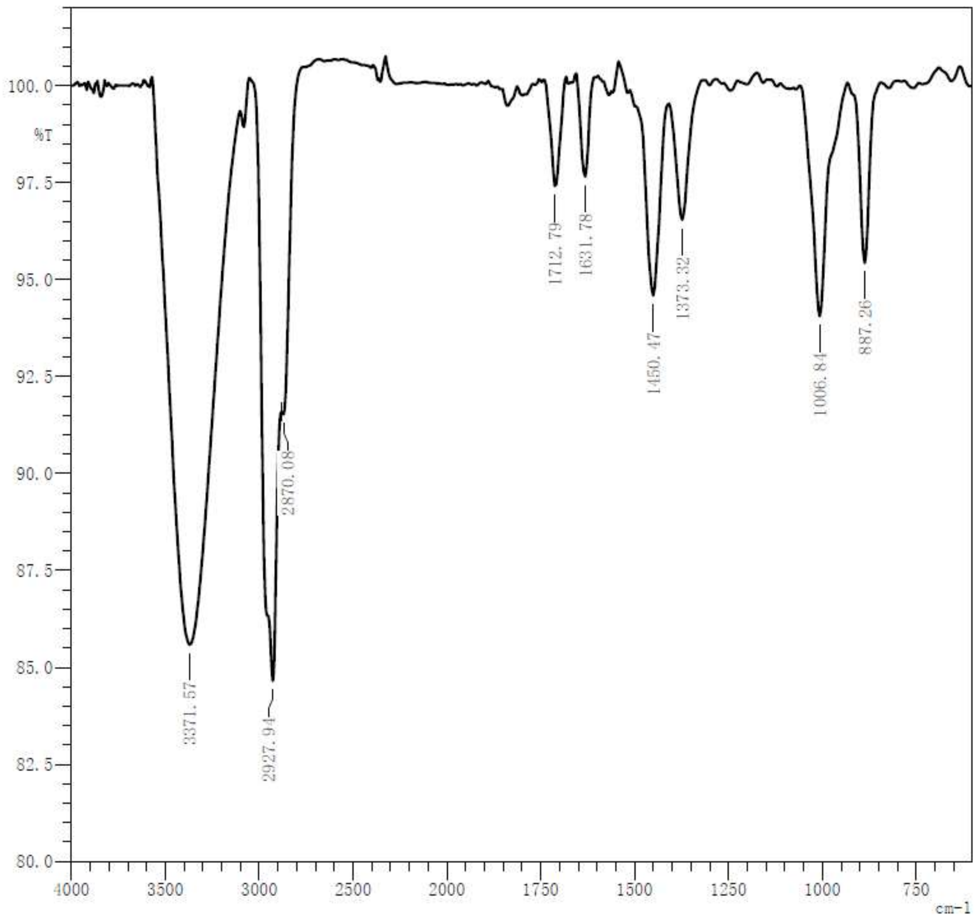

FigureS19. IR spectrum of compound **2**.

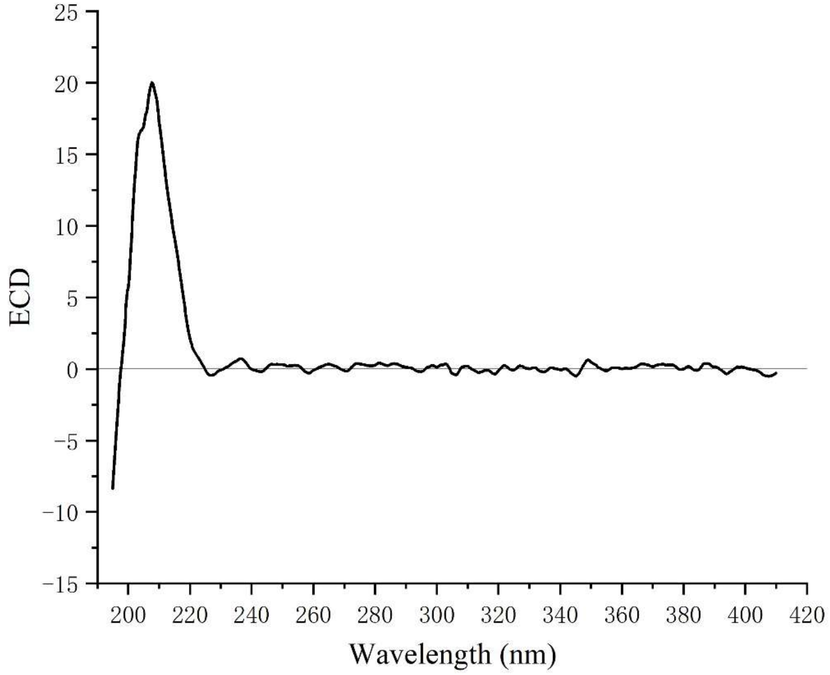

FigureS20. ECD spectrum of compound **2**.

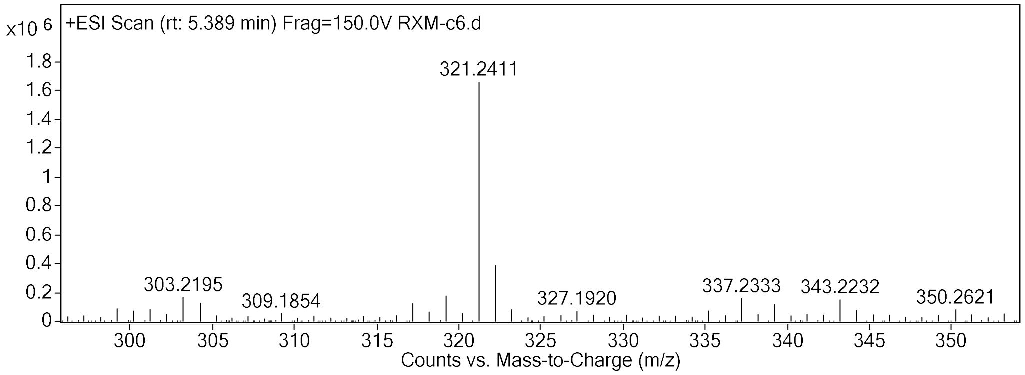

Formular	Score	Mass	Mass (MGF)	*m/z* (Calc)	Diff (ppm)	Ion Formular	*m/z*
$C_{20}H_{32}O_3$	98.05	320.2338	320.2351	321.2424	4.13	$C_{20}H_{33}O_3$	321.2411
$C_{20}H_{32}O_3$	98.47	320.2340	230.2351	343.2244	3.64	$C_{20}H_{32}NaO_3$	343.2232

FigureS21. HRESIMS spectrum of compound **3**.

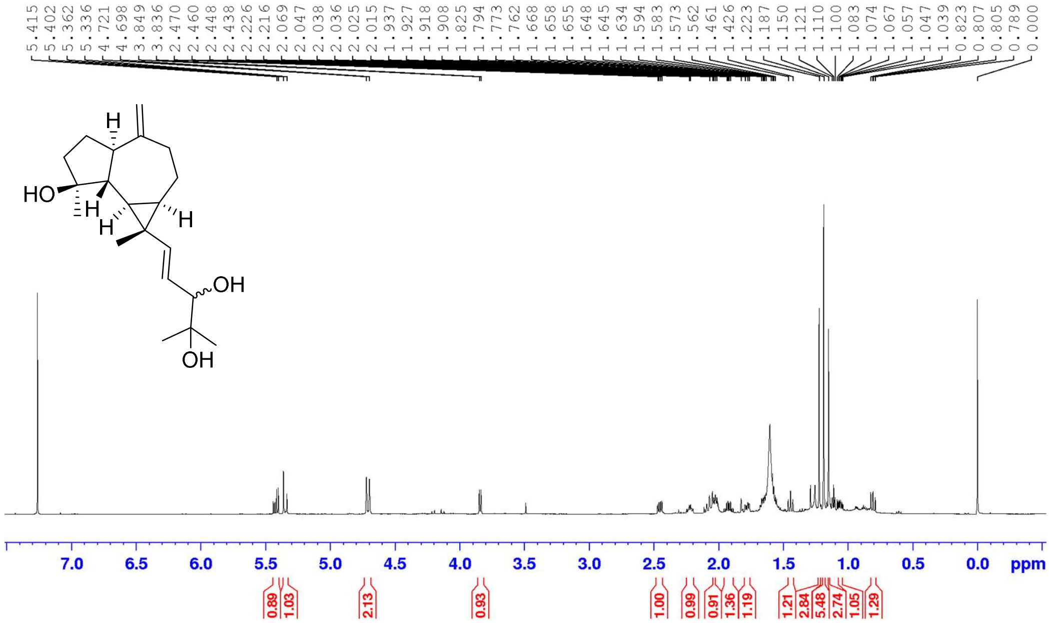

FigureS22. ^{1}H NMR spectrum of compound **3**

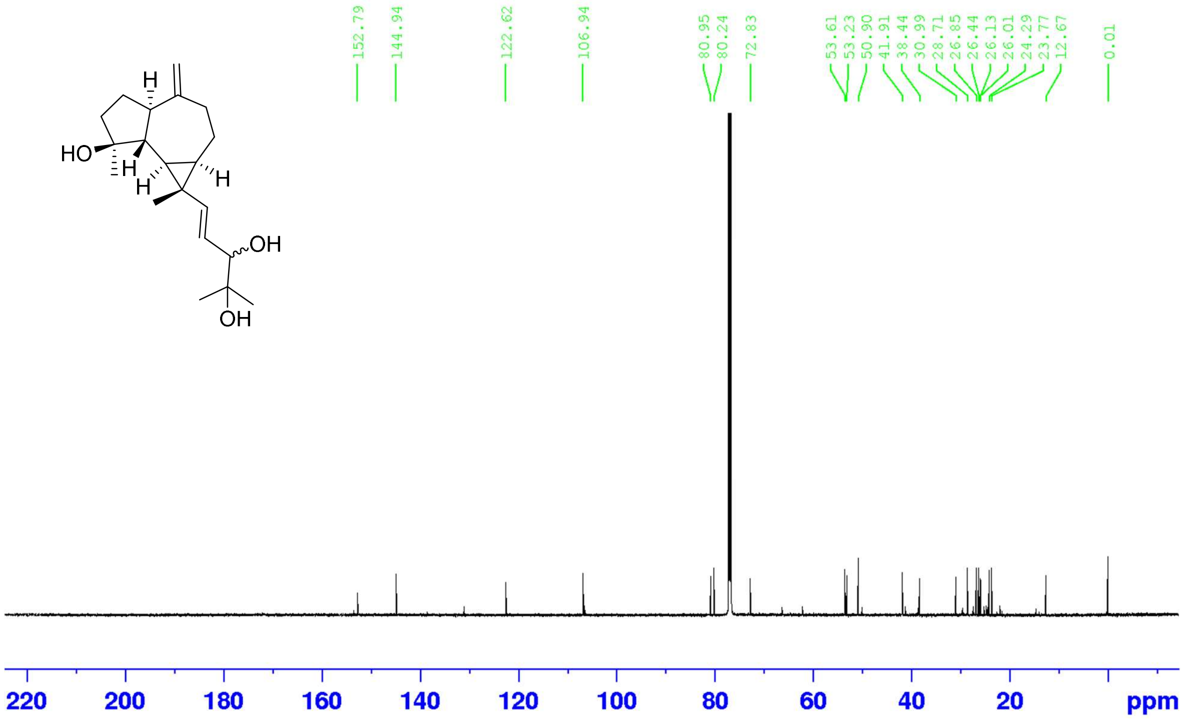

FigureS23. ^{13}C NMR spectrum of compound **3**

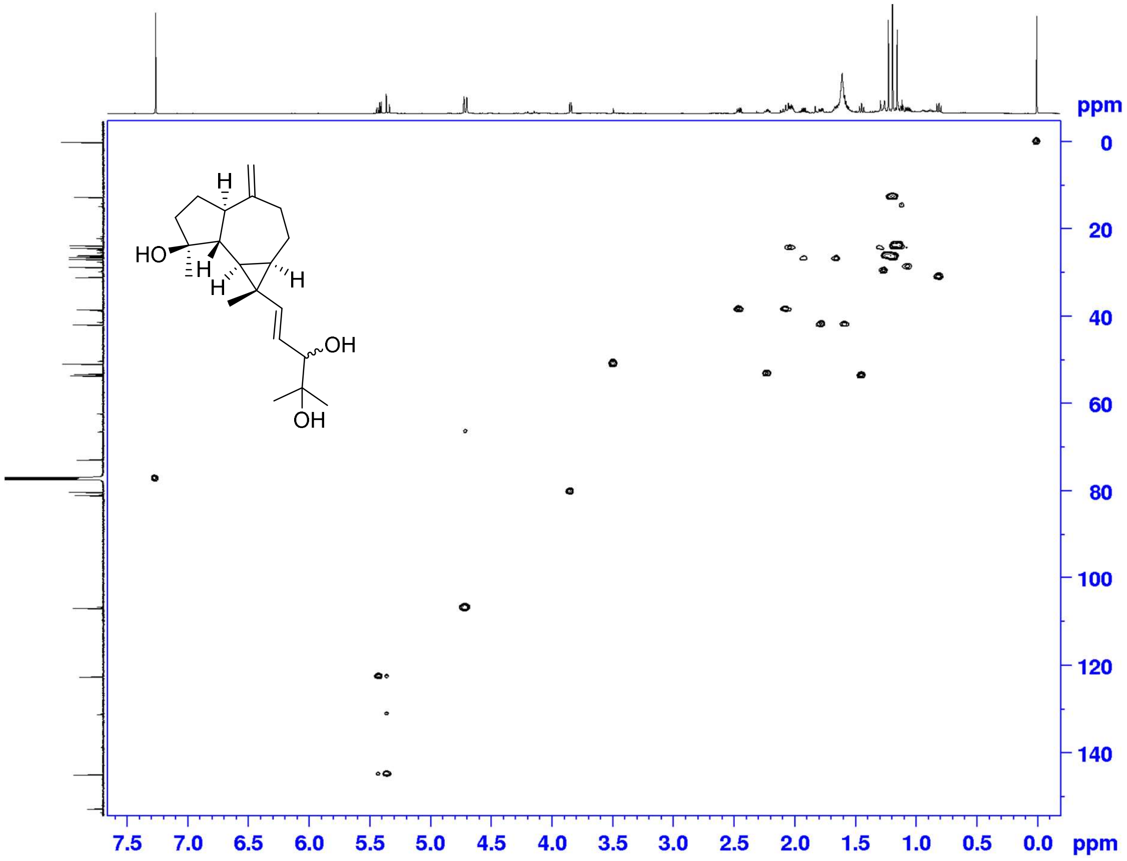

FigureS24. HSQC NMR spectrum of compound **3**

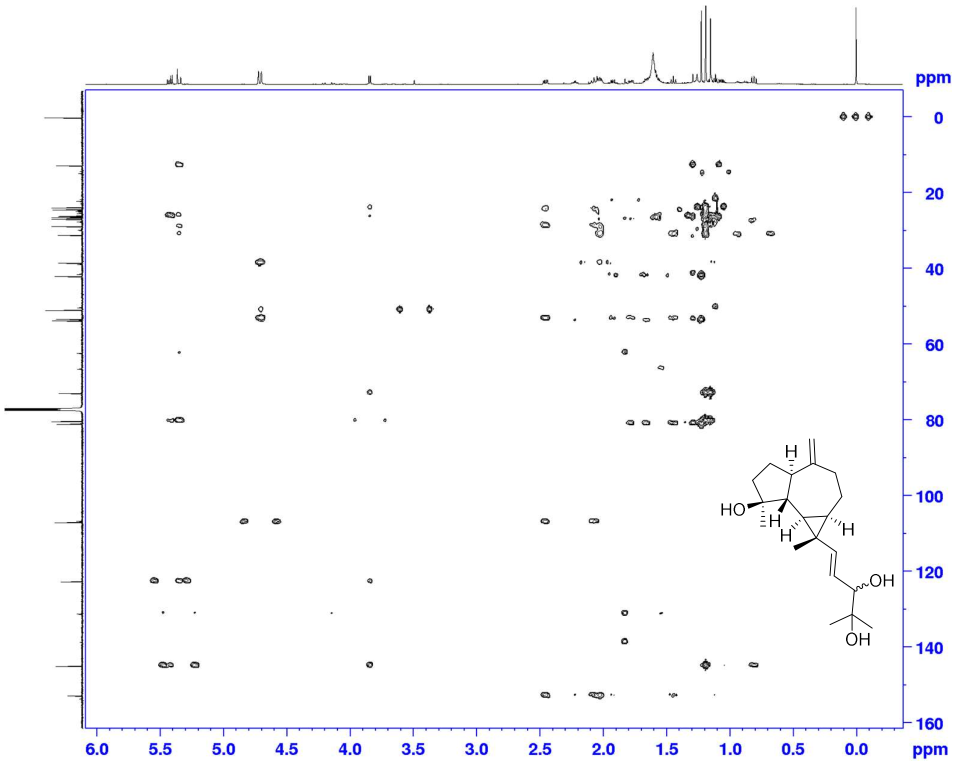

FigureS25. HMBC NMR spectrum of compound **3**

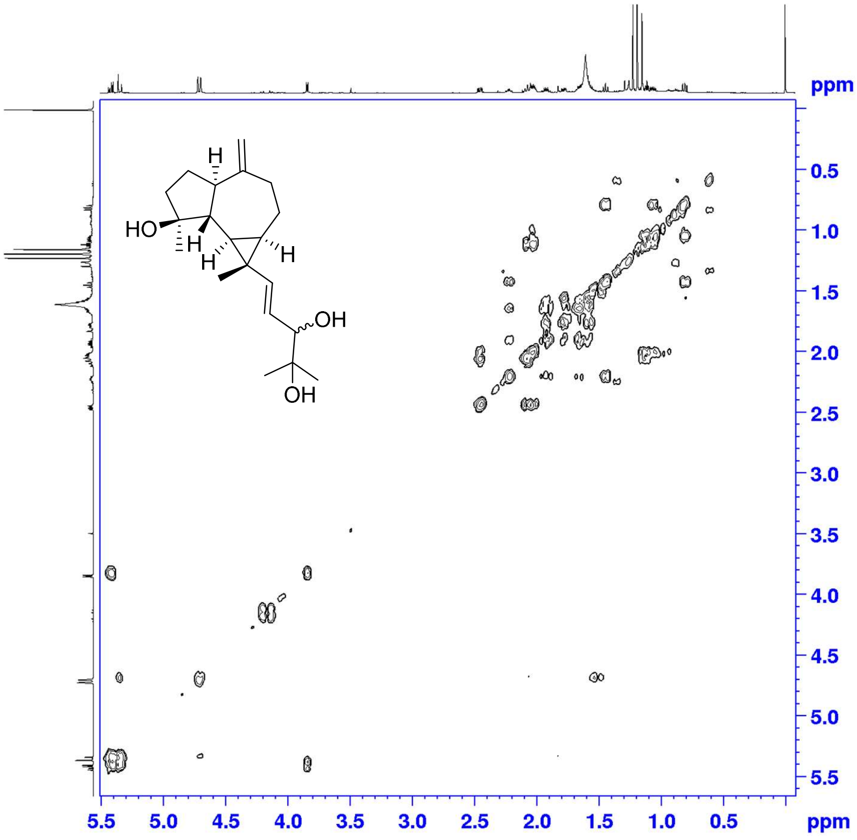

FigureS26. ^{1}H-^{1}H COSY NMR spectrum of compound **3**

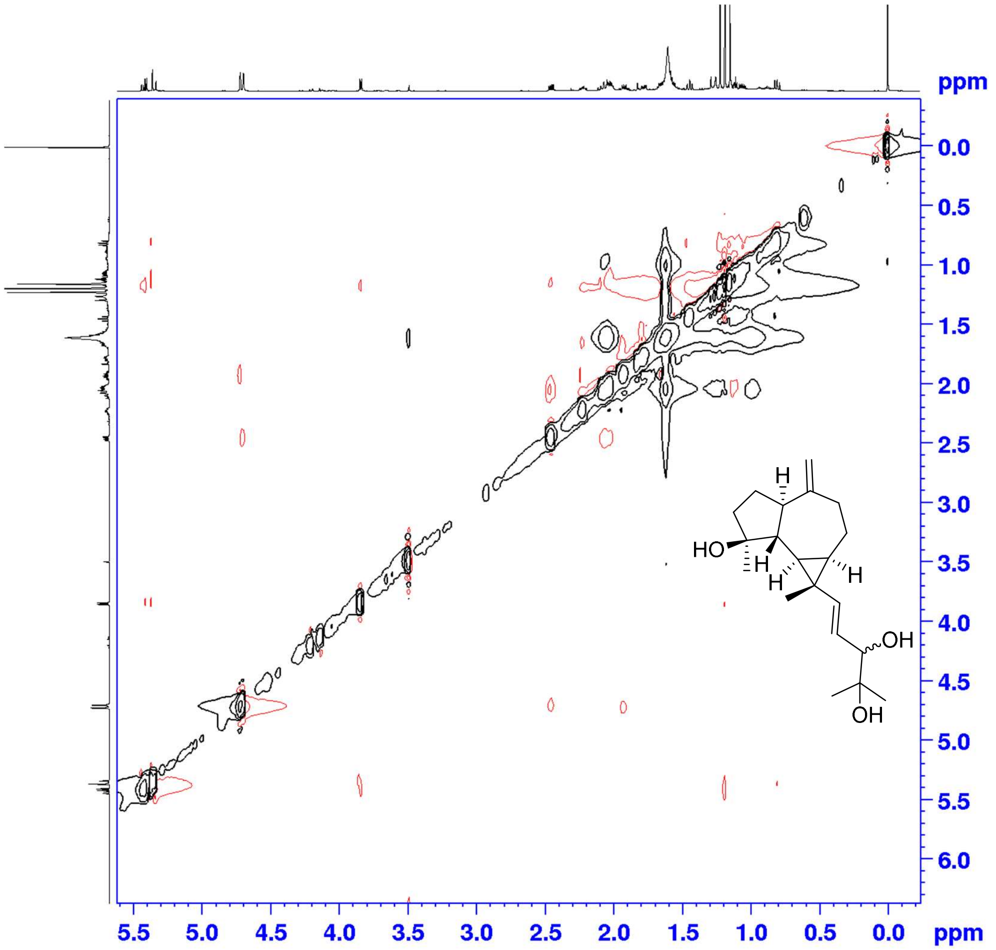

FigureS27. NOESY NMR spectrum of compound **3**

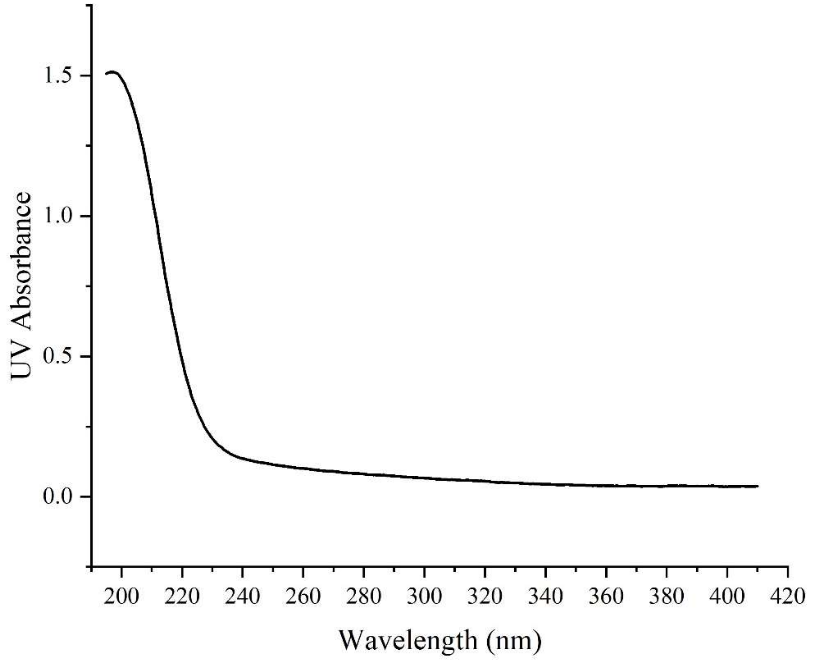

FigureS28. UV spectrum of compound **3**

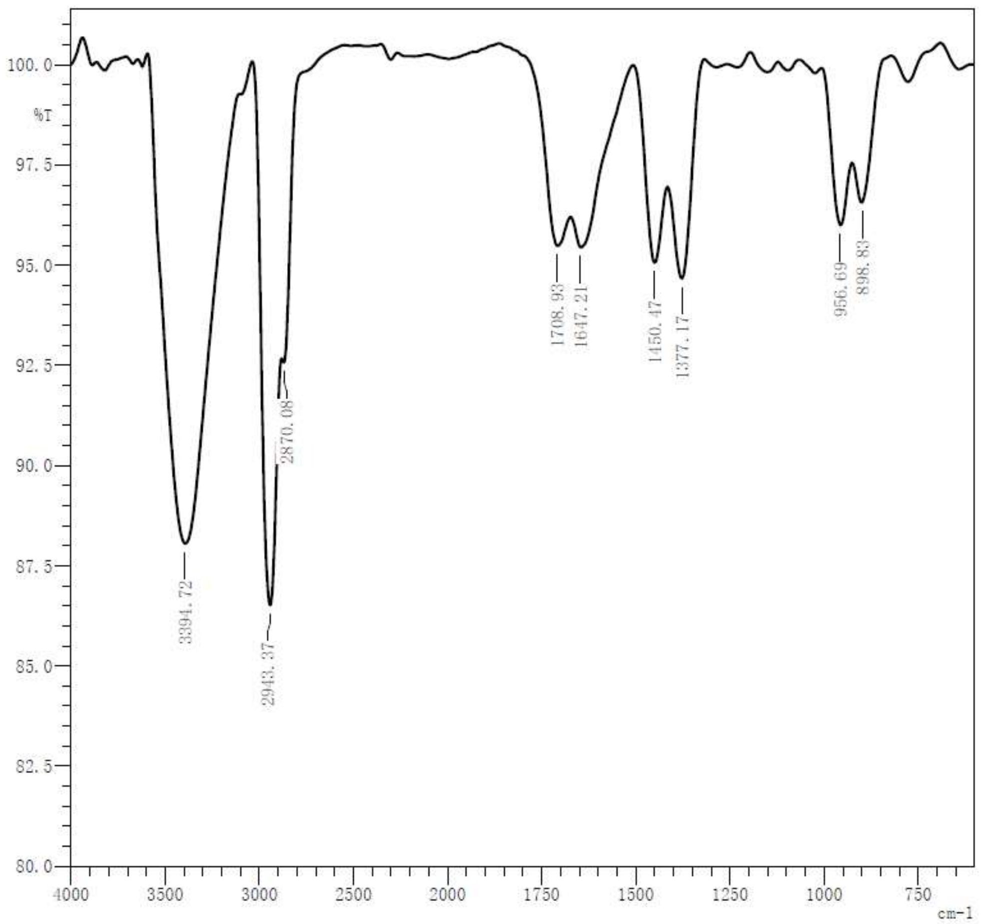

FigureS29. IR spectrum of compound **3**.

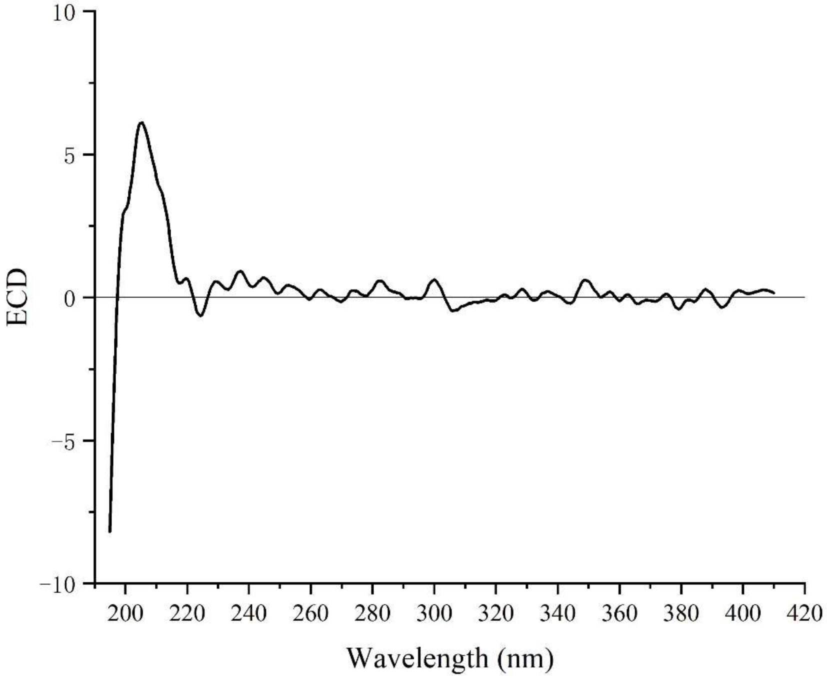

FigureS30. ECD spectrum of compound **3**

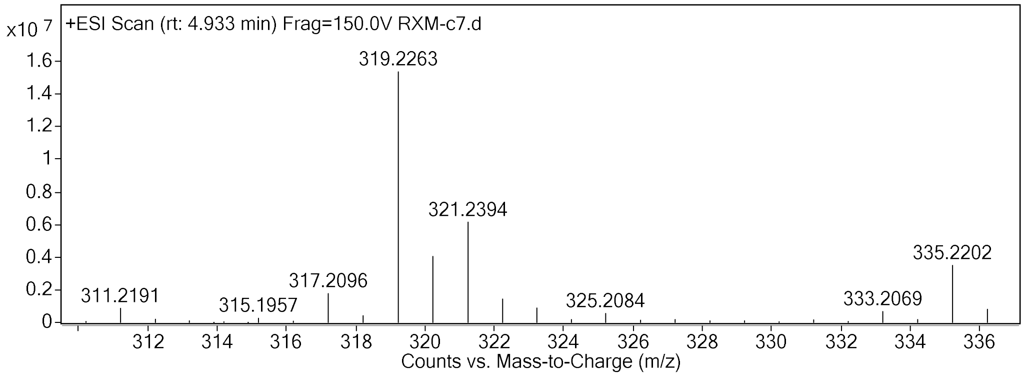

Formular	Score	Mass	Mass (MGF)	*m/z* (Calc)	Diff (ppm)	Ion Formular	*m/z*
$C_{20}H_{30}O_3$	98.47	318.2184	318.2195	319.2268	3.37	$C_{20}H_{31}O_3$	319.2263

FigureS31. HRESIMS spectrum of compound **4**.

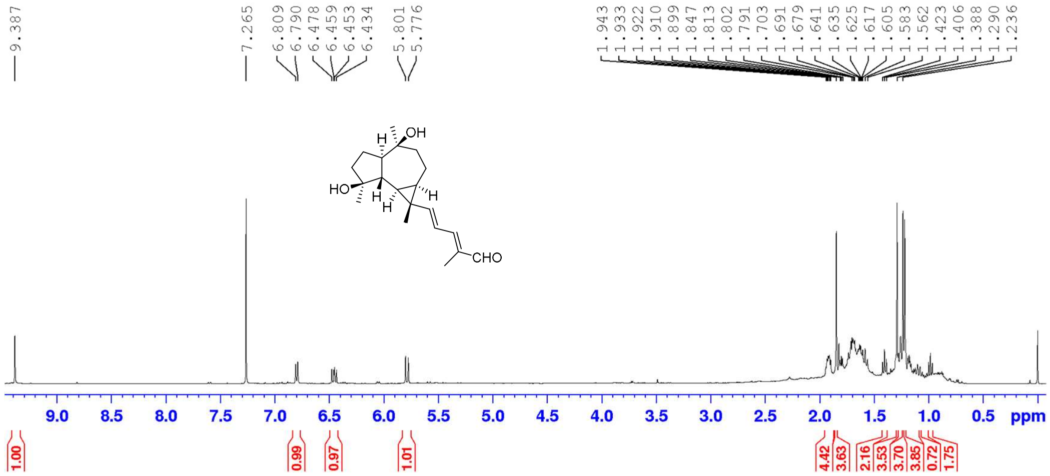

FigureS32. ^{1}H NMR spectrum of compound **4**

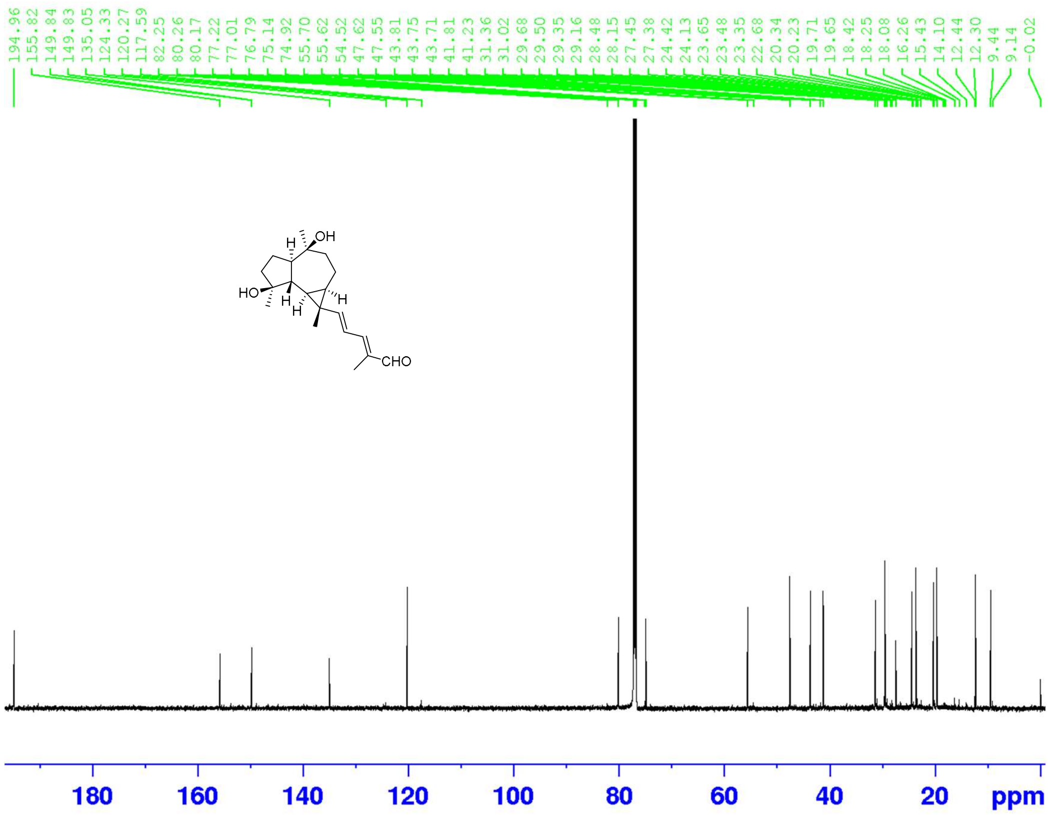

FigureS33. ^{13}C-NMR spectrum of Compound **4**

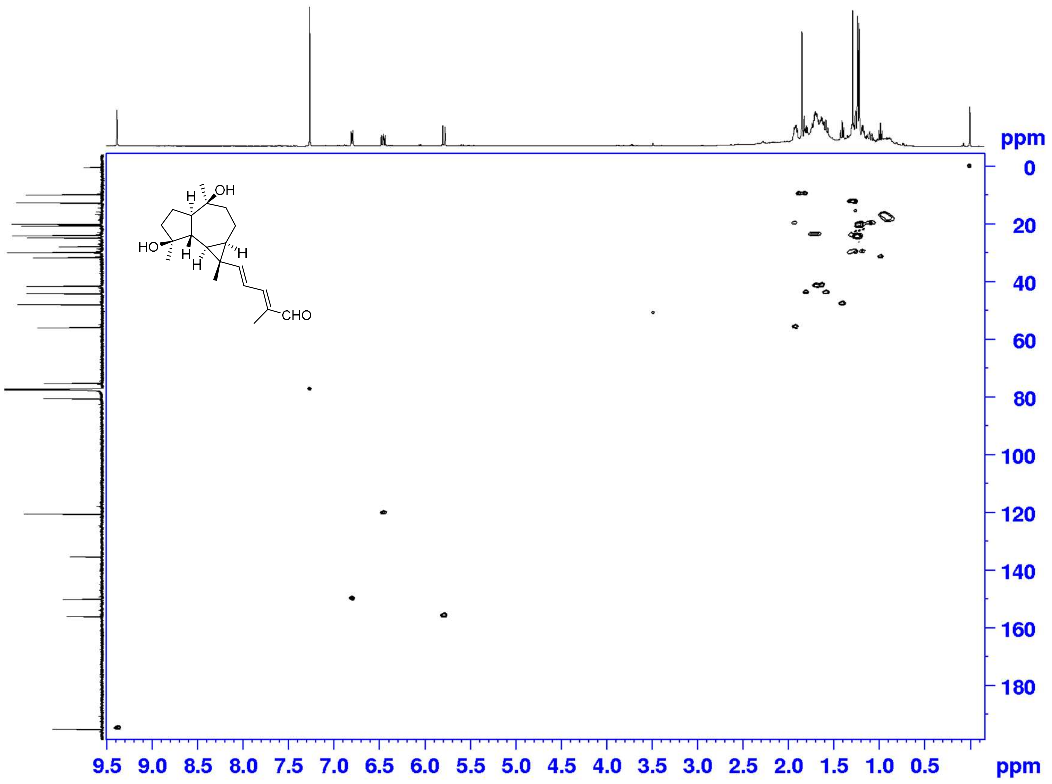

FigureS34. HSQC NMR spectrum of compound **4**

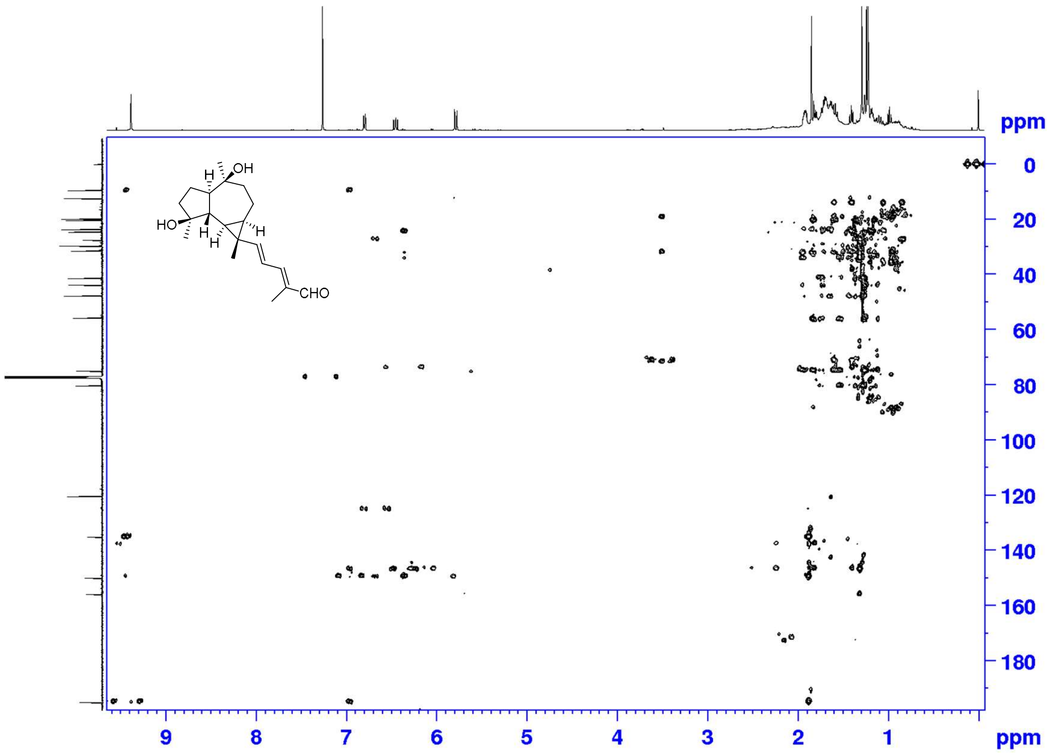

FigureS35. HMBC NMR spectrum of compound **4**

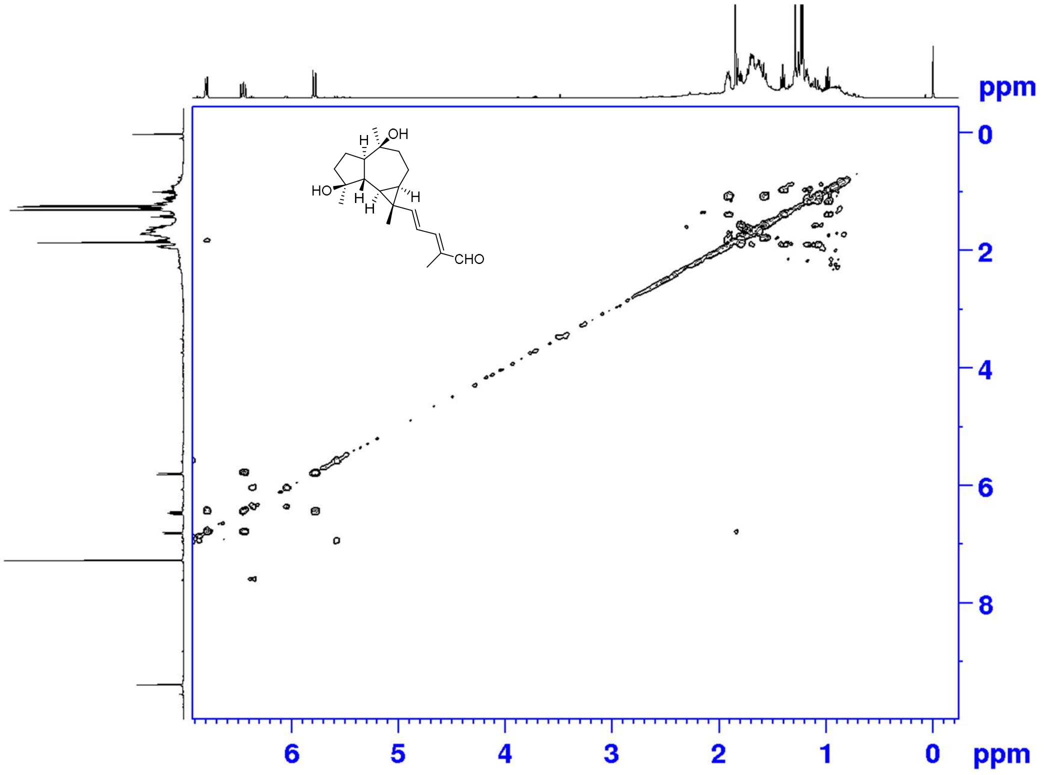

FigureS36. ^{1}H-^{1}H COSY NMR spectrum of compound **4**

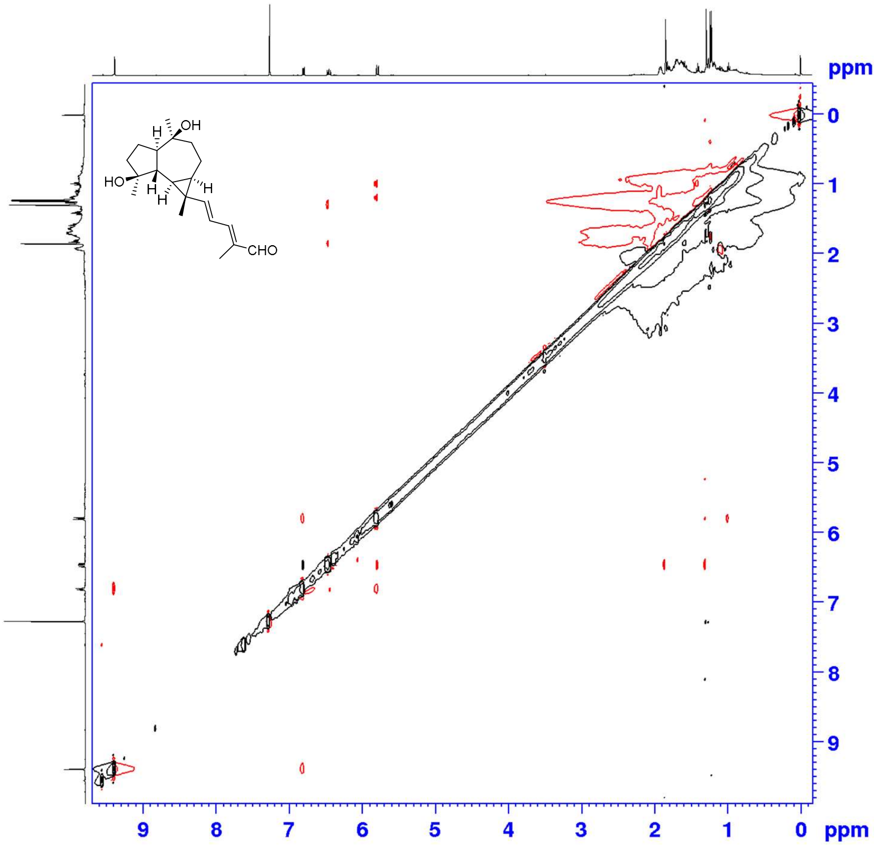

FigureS37. NOESY NMR spectrum of compound **4**

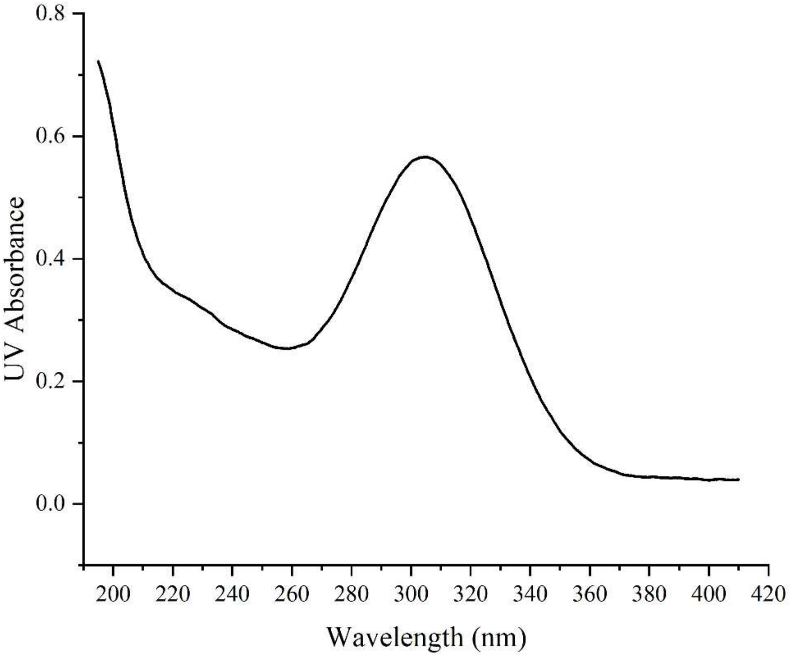

FigureS38. UV spectrum of compound **4**

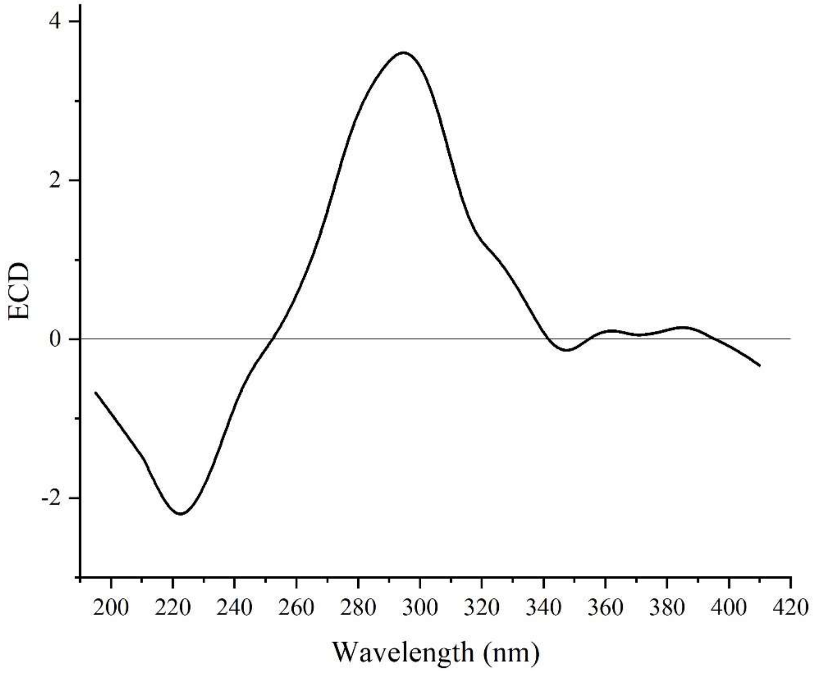

FigureS39. ECD spectrum of compound **4**

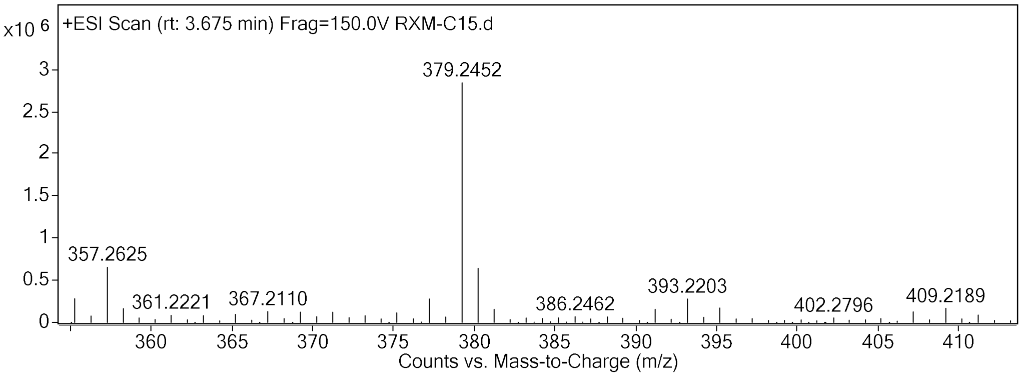

Formular	Score	Mass	Mass (MGF)	*m/z* (Calc)	Diff (ppm)	Ion Formular	*m/z*
$C_{20}H_{36}O_5$	98.90	356.2552	356.2563	357.2636	2.95	$C_{20}H_{37}O_5$	357.2625
$C_{20}H_{36}O_5$	99.91	356.2560	356.2563	379.2455	0.83	$C_{20}H_{36}NaO_5$	379.2452

FigureS40. HRESIMS spectrum of compound **5**.

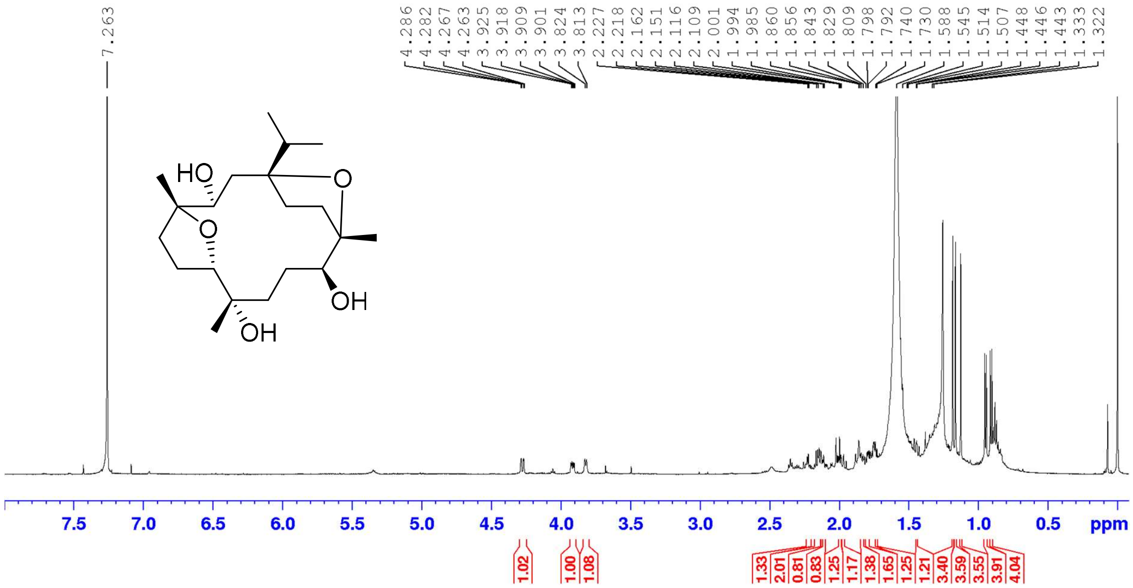

FigureS41. ^{1}H NMR spectrum of compound **5**

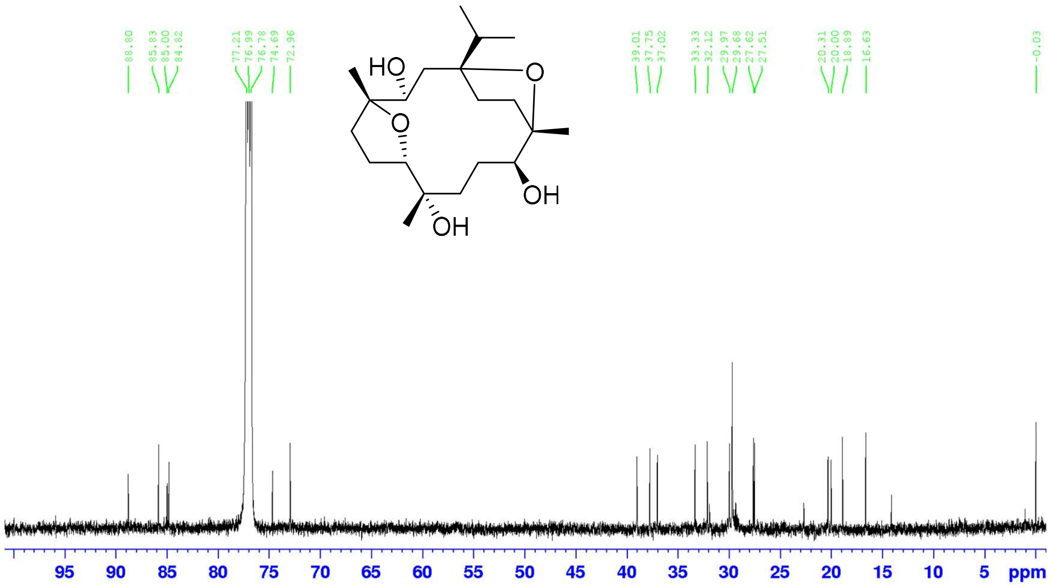

FigureS42. ^{13}C NMR spectrum of compound **5**

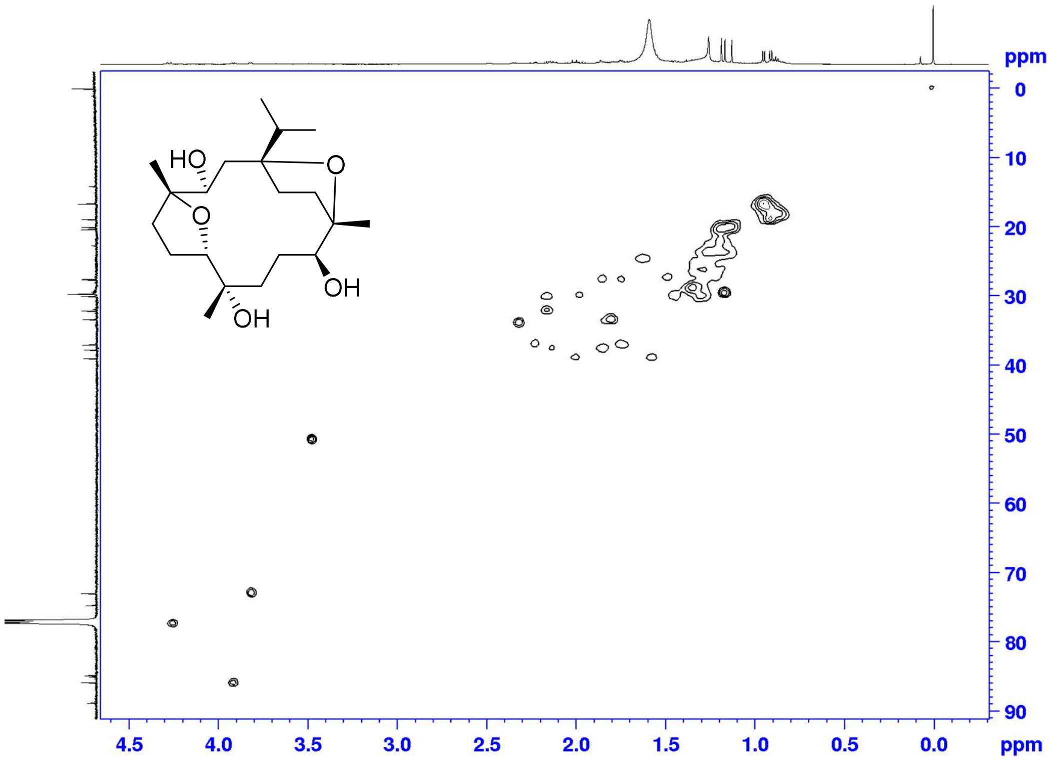

FigureS43. HSQC NMR spectrum of compound **5**

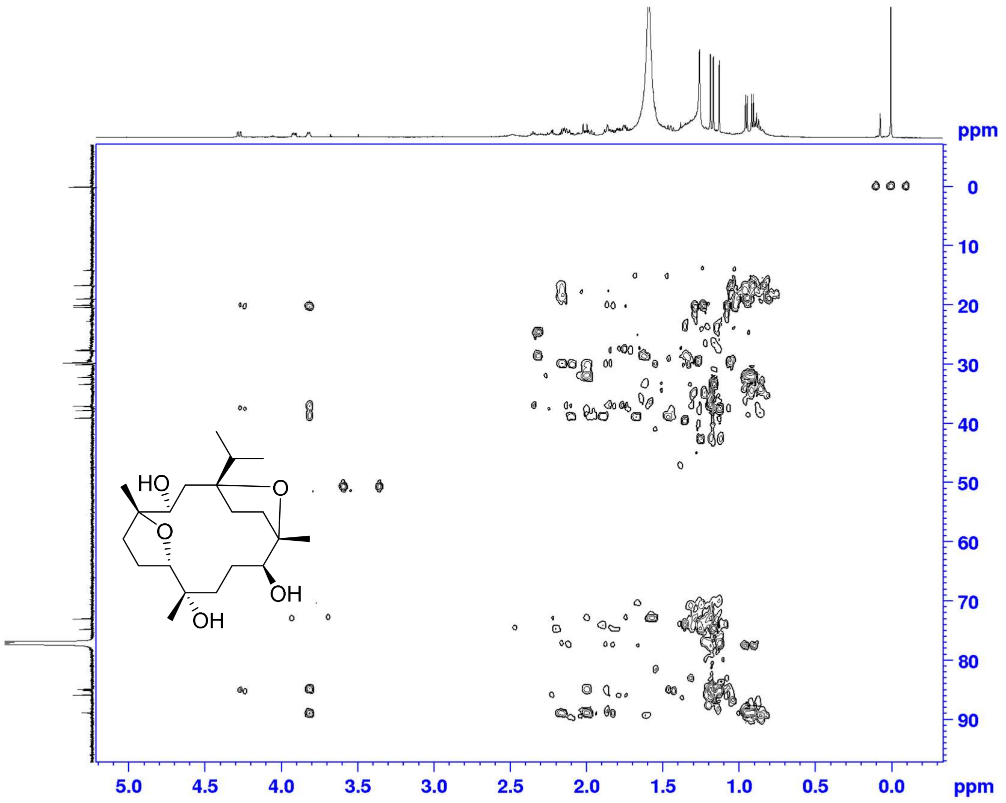

FigureS44. HMBC NMR spectrum of compound **5**

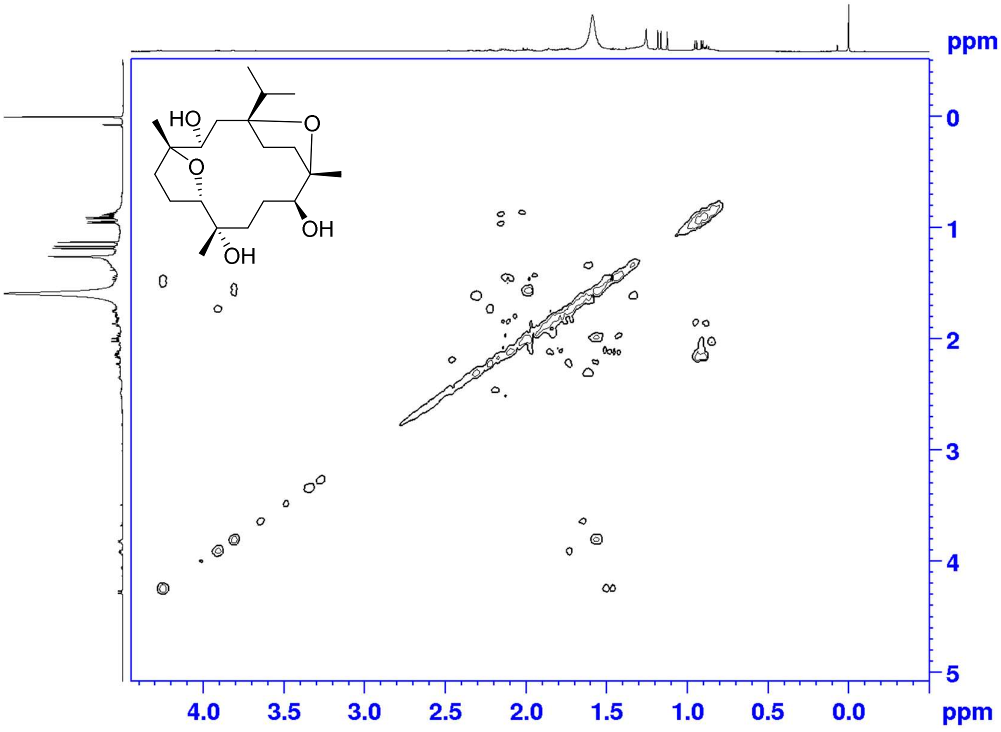

FigureS45. ^{1}H-^{1}H COSY NMR spectrum of compound **5**

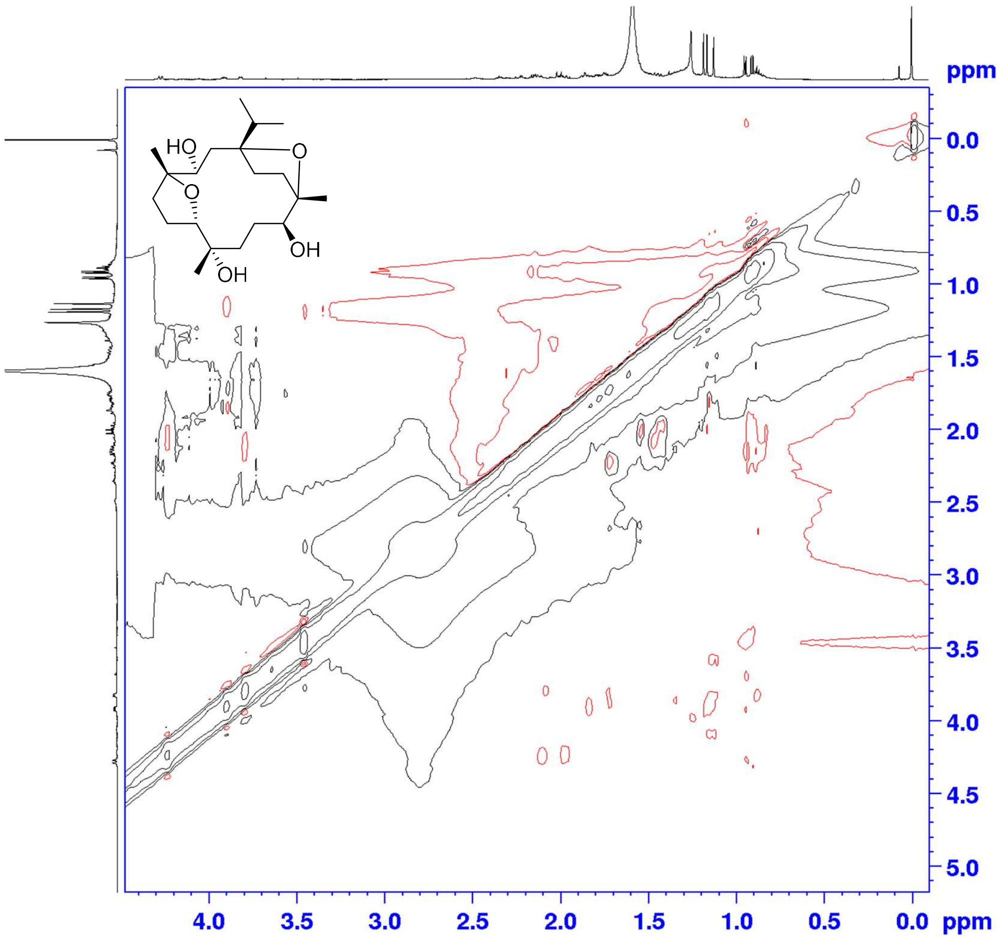

FigureS46. NOESY NMR spectrum of compound **5**.

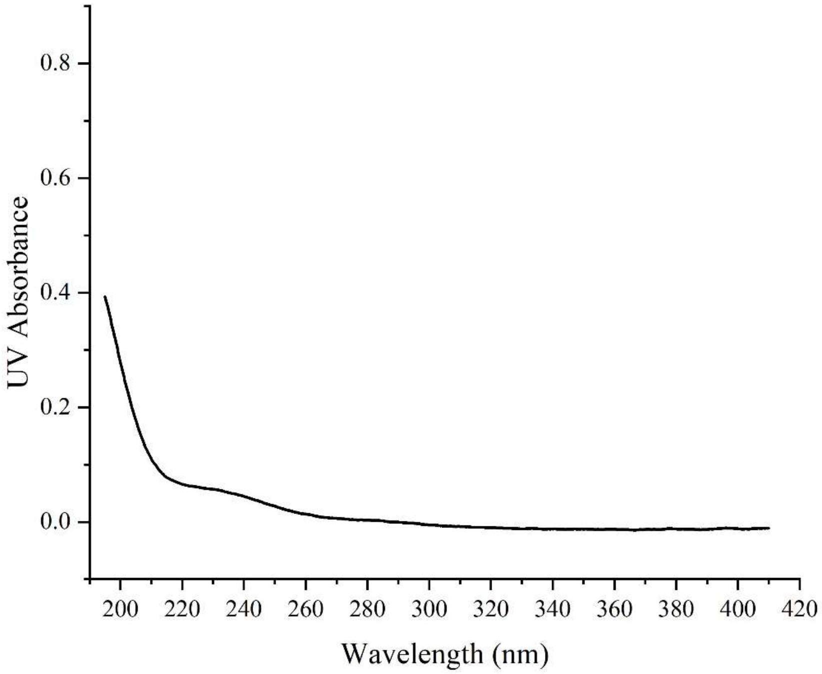

FigureS47. UV spectrum of compound **5**

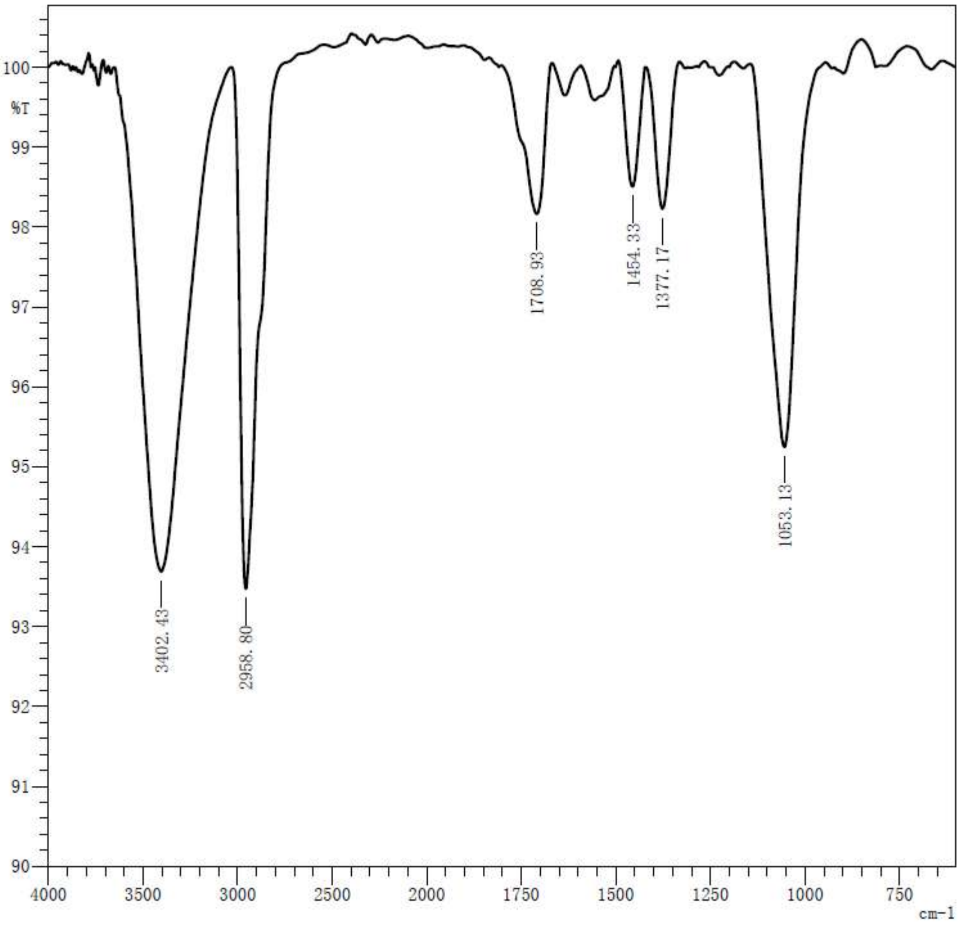

FigureS48. IR spectrum of compound **5**.

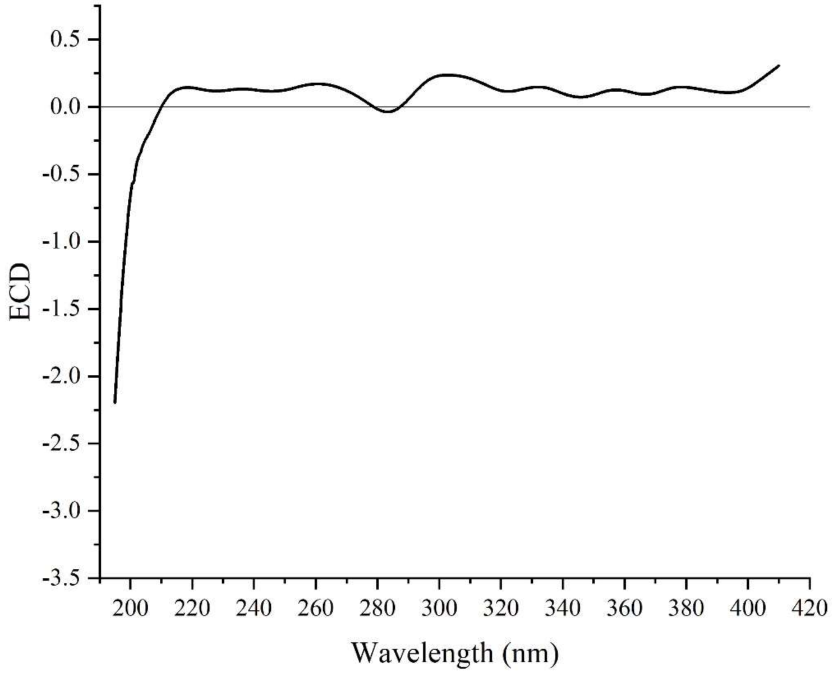

FigureS49. ECD spectrum of compound **5**

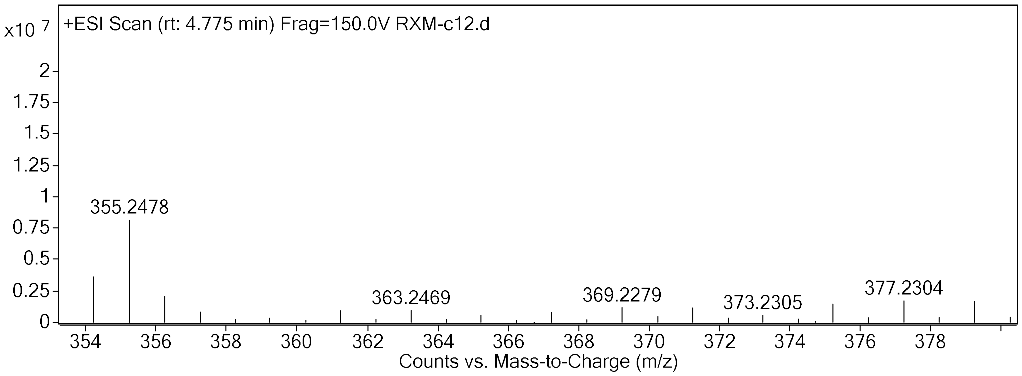

Formular	Score	Mass	Mass (MGF)	*m/z* (Calc)	Diff (ppm)	Ion Formular	*m/z*
$C_{20}H_{34}O_5$	99.7	354.2405	354.2406	355.2479	0.28	$C_{20}H_{35}O_5$	355.2478
$C_{20}H_{34}O_5$	99.69	354.2412	354.2406	377.2298	-1.57	$C_{20}H_{34}NaO_5$	377.2304

FigureS50. HRESIMS spectrum of compound **6.**

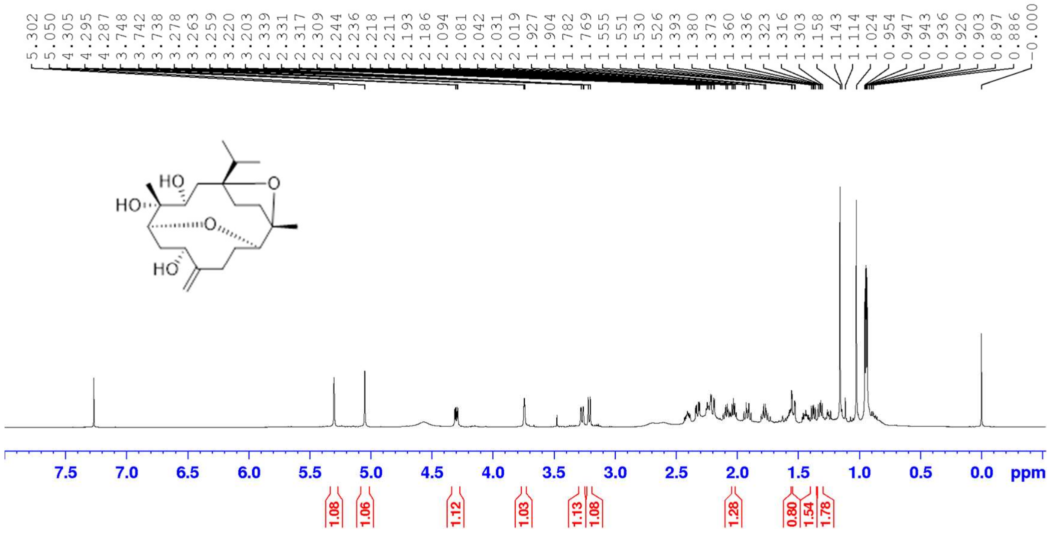

FigureS51. ^{1}H NMR spectrum of compound **6**

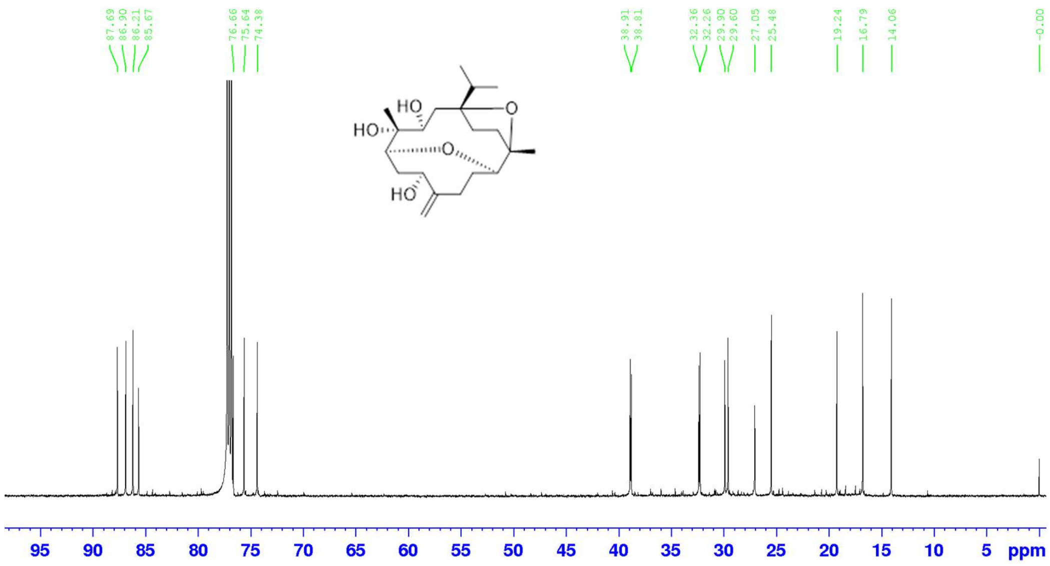

FigureS52. ^{13}C NMR spectrum of compound **6**

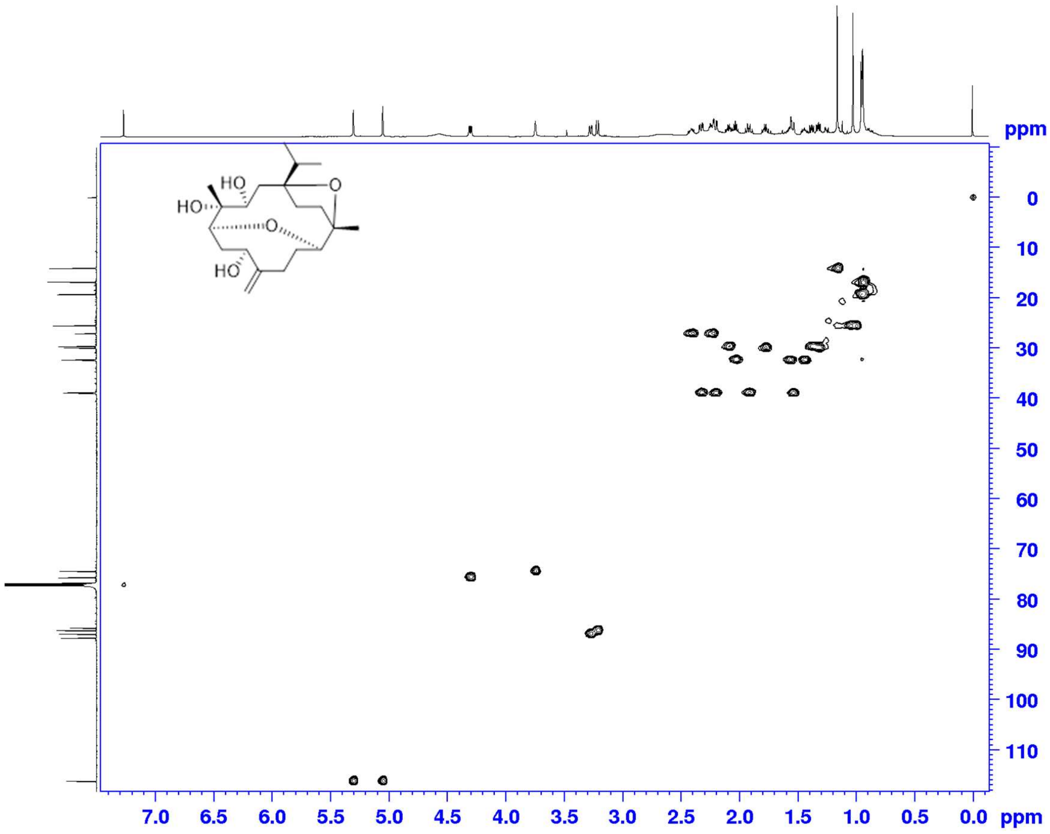

FigureS53. HSQC NMR spectrum of compound **6**

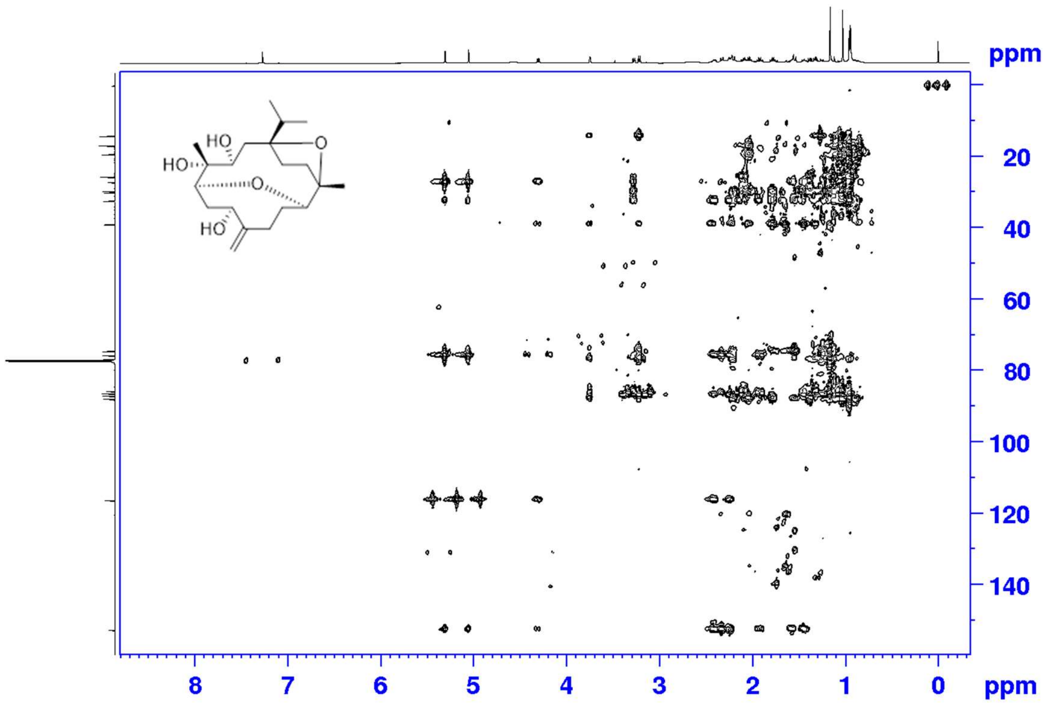

FigureS54. HMBC NMR spectrum of compound **6**

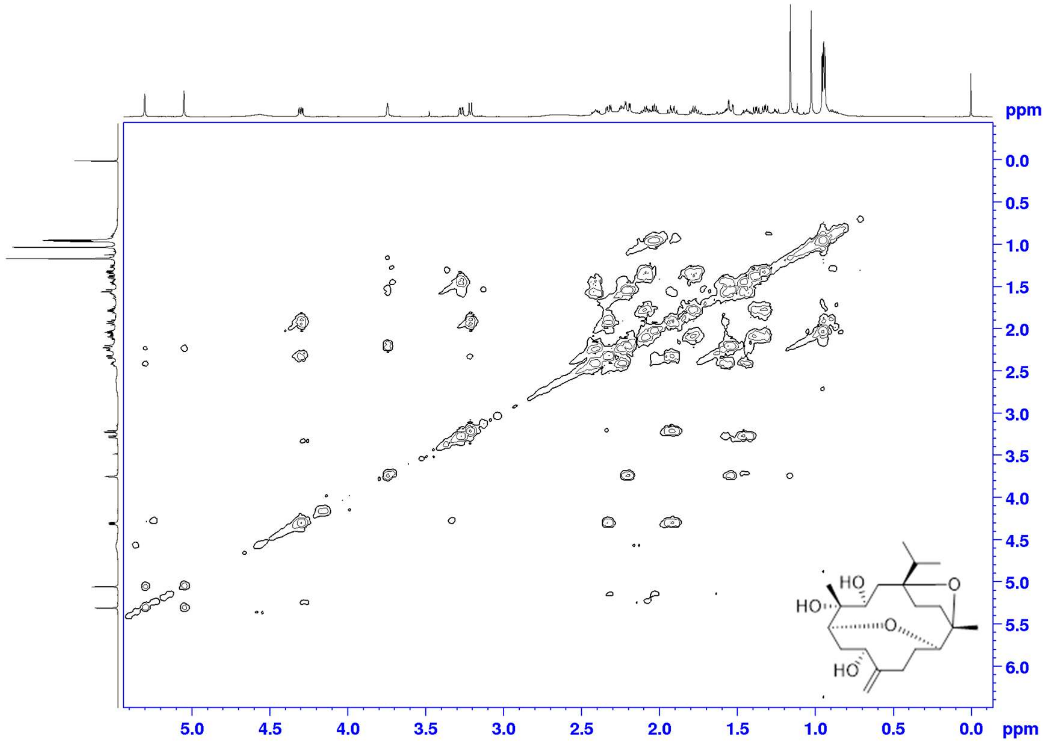

FigureS55. ^1H-^1H COSY NMR spectrum of compound **6**

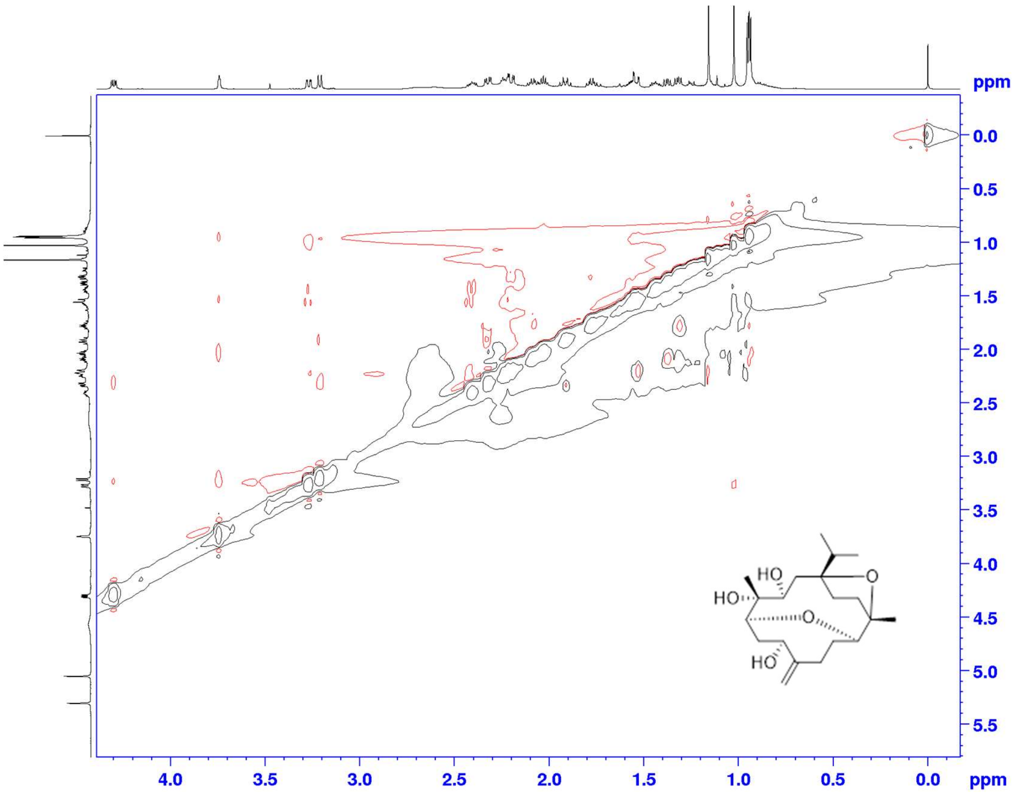

FigureS56. NOESY NMR spectrum of compound **6**

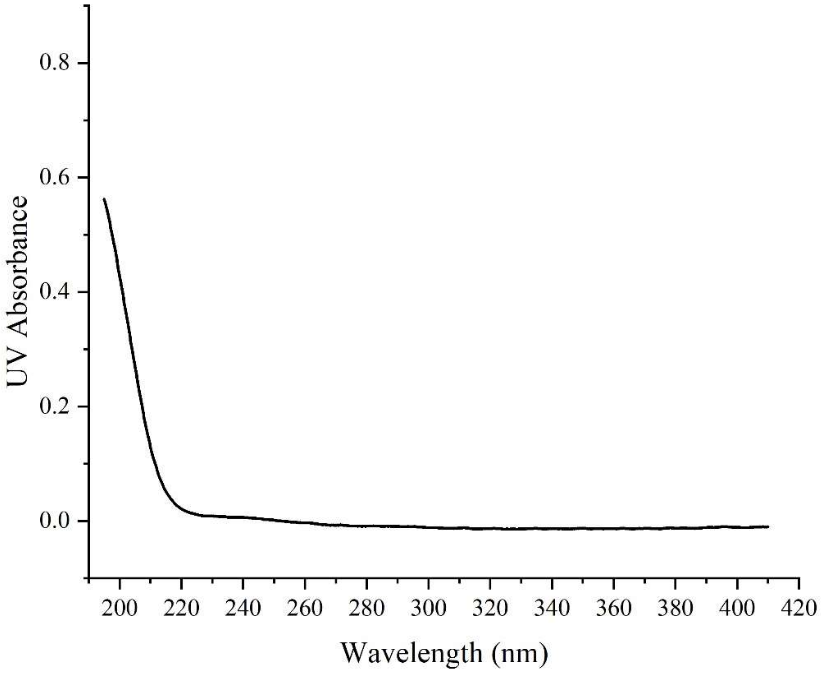

FigureS57. UV spectrum of compound **6**

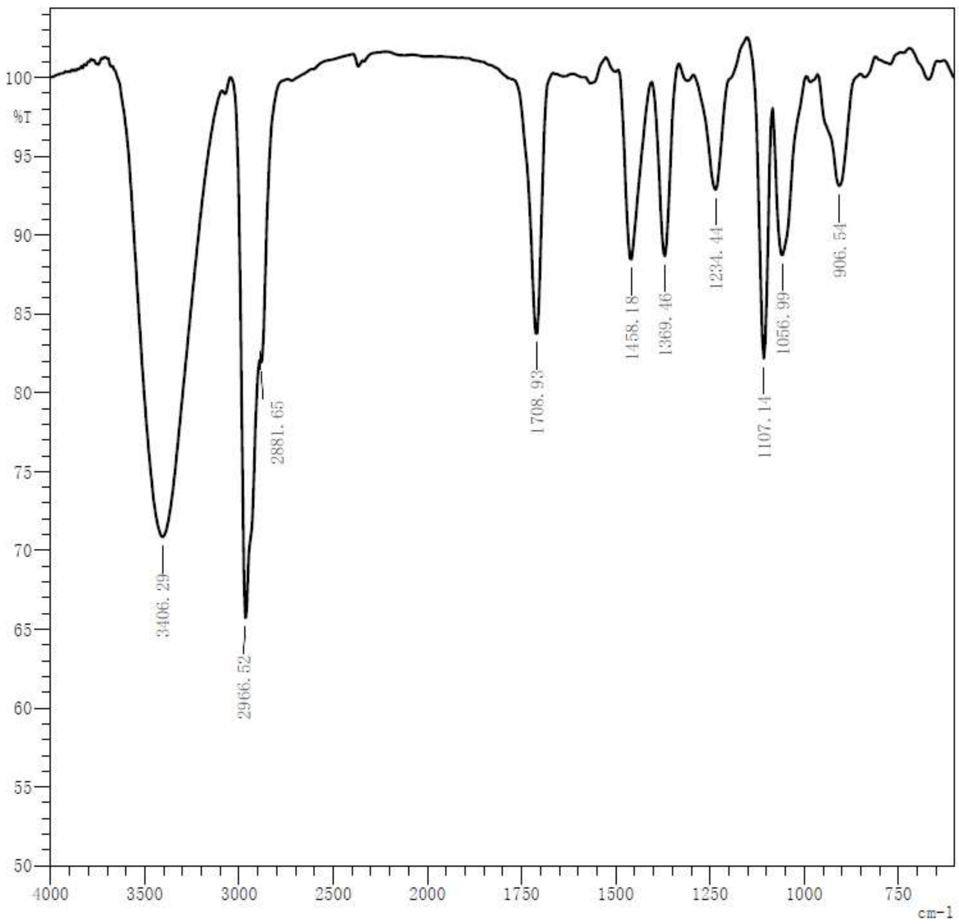

FigureS58. IR spectrum of compound **6**

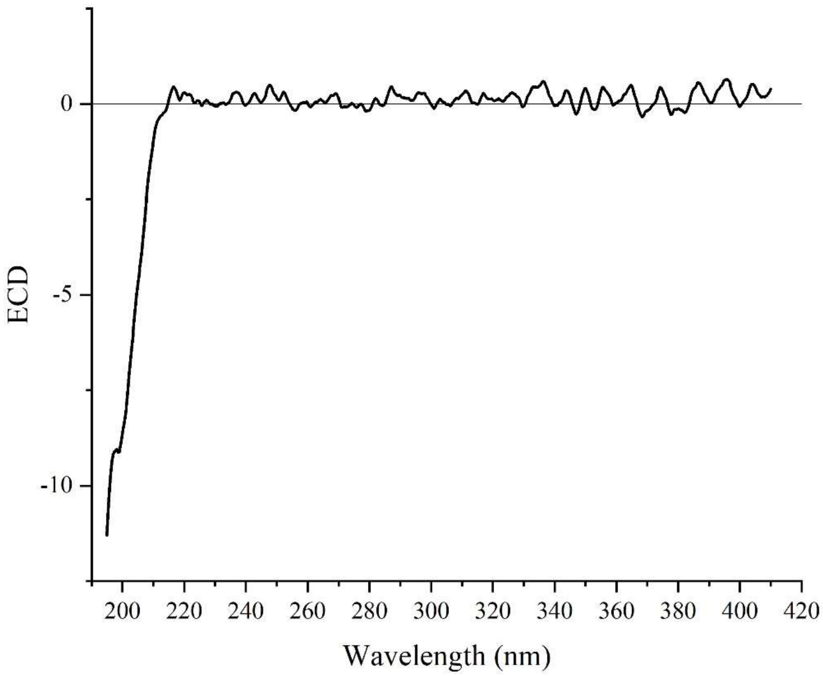

FigureS59. ECD spectrum of compound **6**.

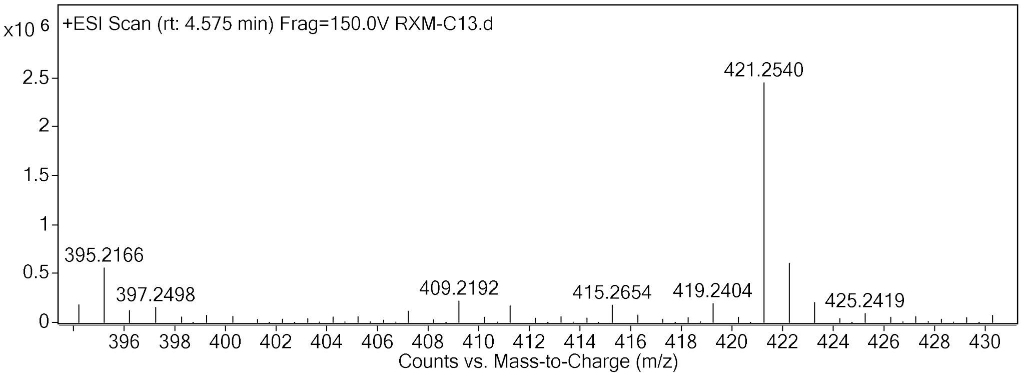

Formular	Score	Mass	Mass (MGF)	*m/z* (Calc)	Diff (ppm)	Ion Formular	*m/z*
$C_{22}H_{38}O_6$	96.51	398.2648	398.2668	421.2561	5.17	$C_{22}H_{38}NaO_6$	421.2540

FigureS60. HRESIMS spectrum of compound **7**.

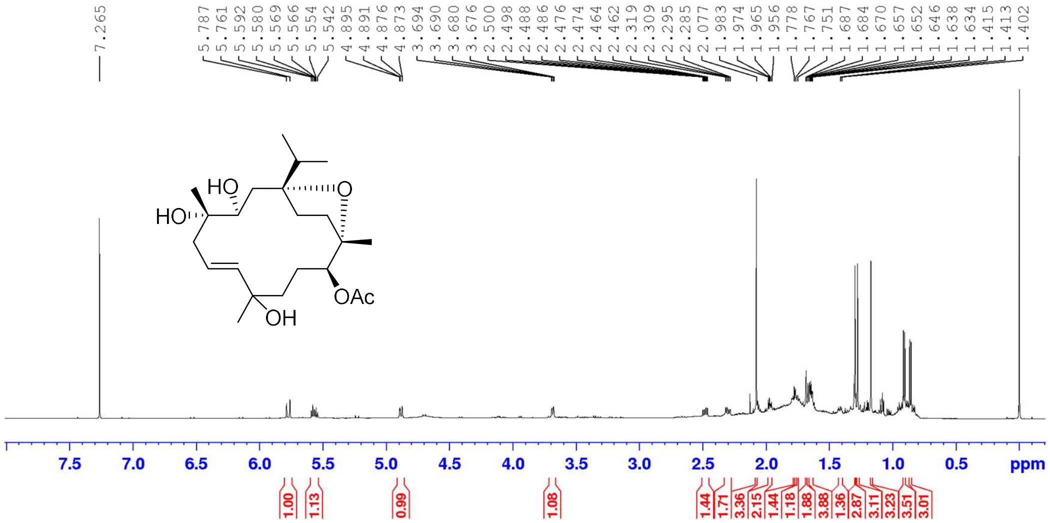

FigureS61. ^{1}H NMR spectrum of compound **7**

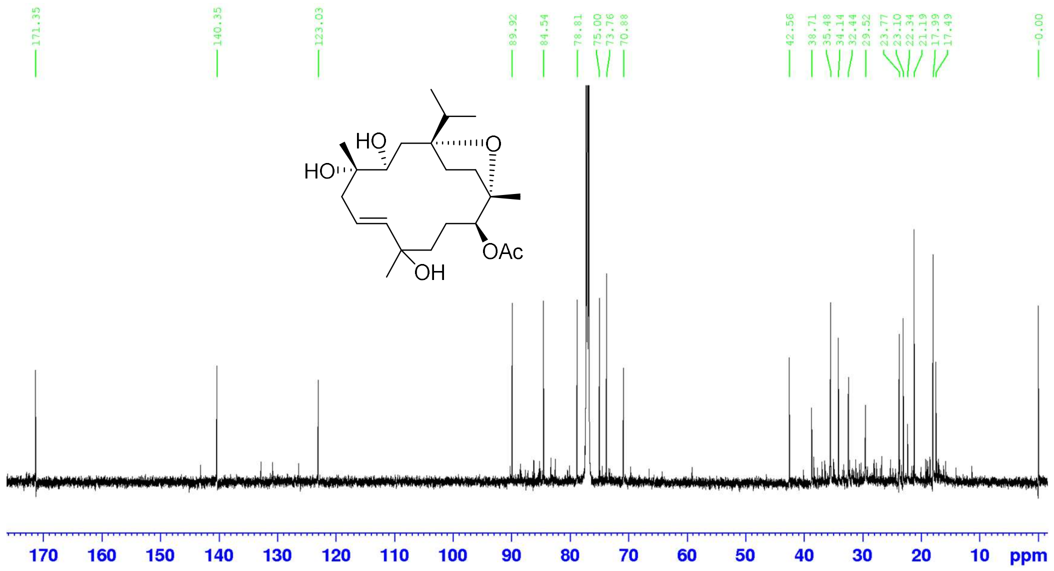

FigureS62. ^{13}C NMR spectrum of compound **7**

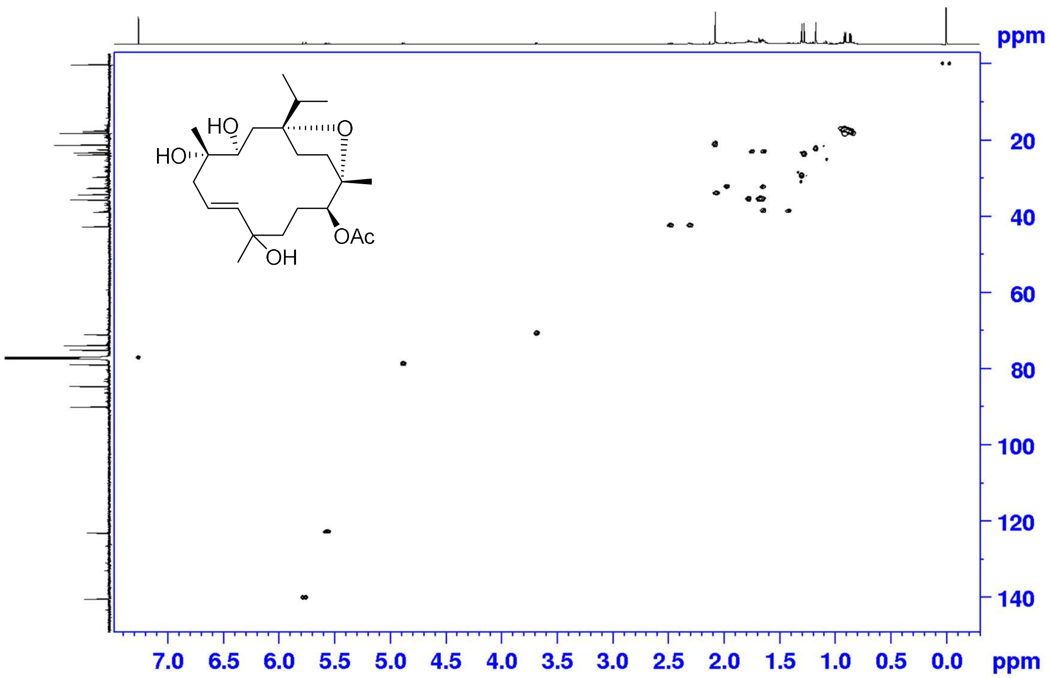

FigureS63. HSQC NMR spectrum of compound **7**

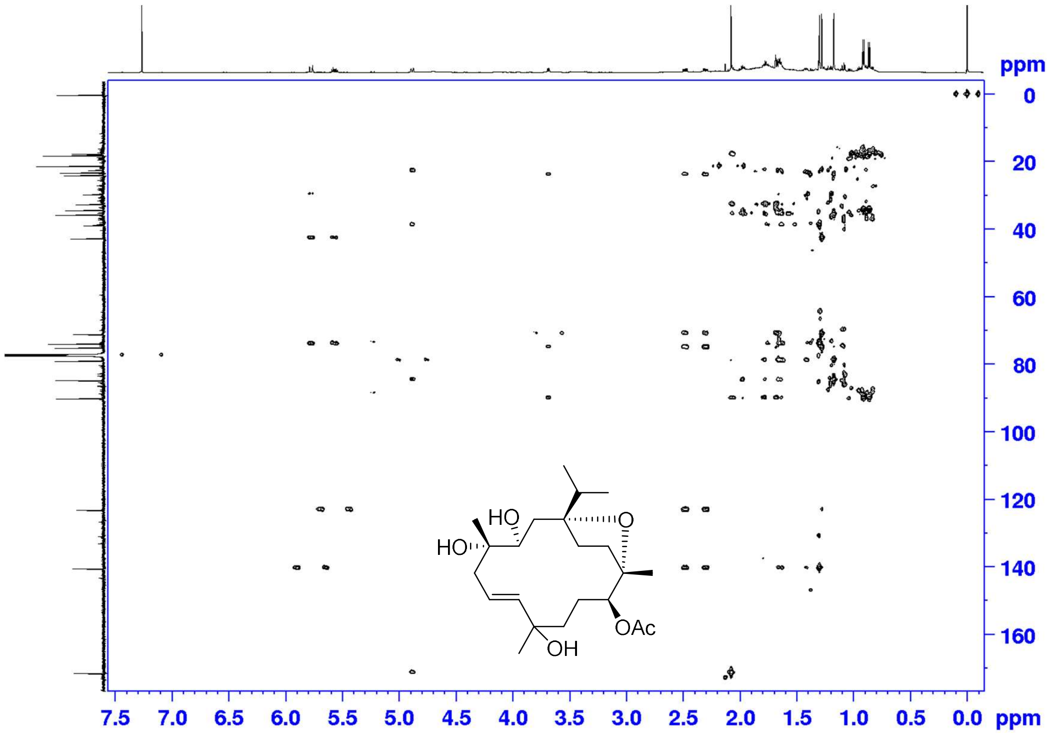

FigureS64. HMBC NMR spectrum of compound **7**

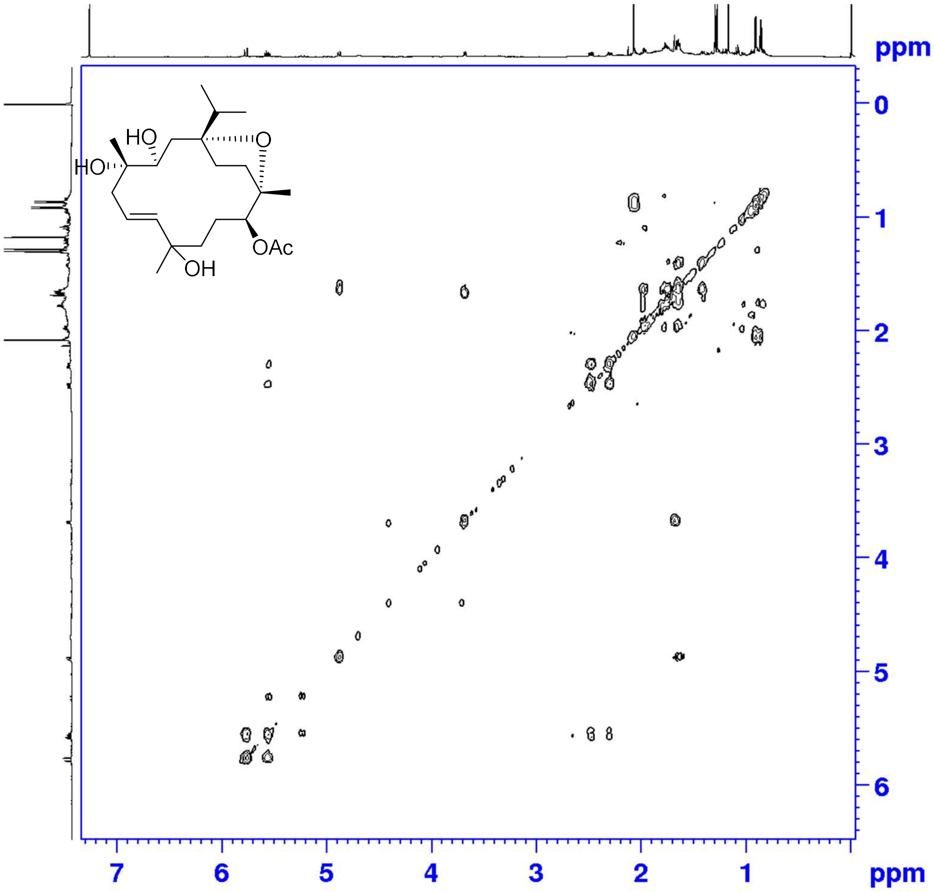

FigureS65. ^{1}H-^{1}H COSY NMR spectrum of compound **7**

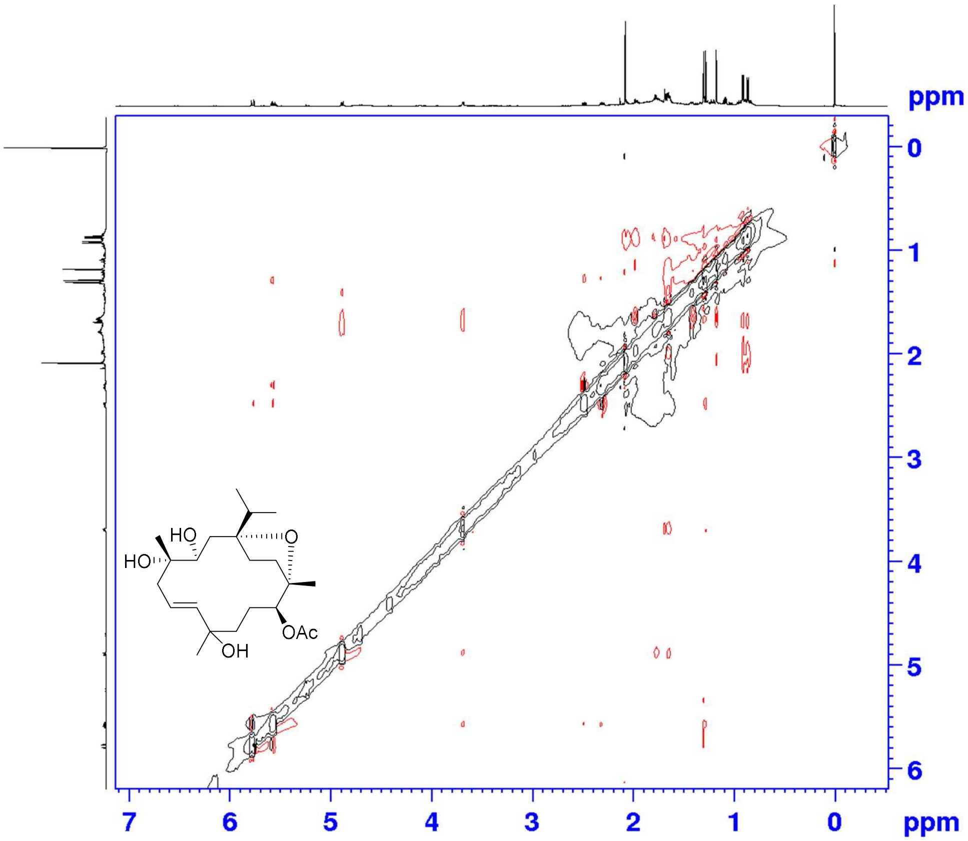

FigureS66. NOESY NMR spectrum of compound **7**

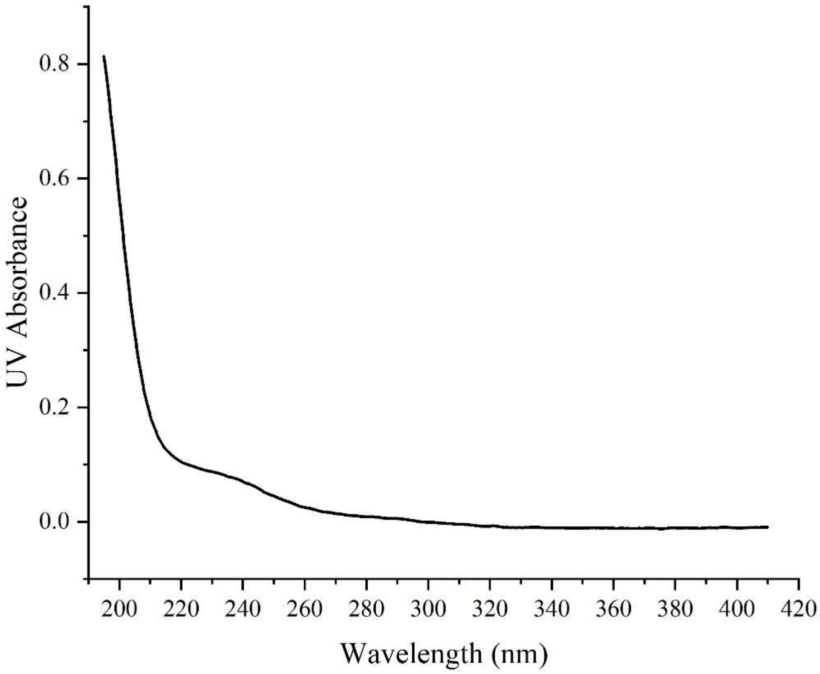

FigureS67. UV spectrum of compound **7**

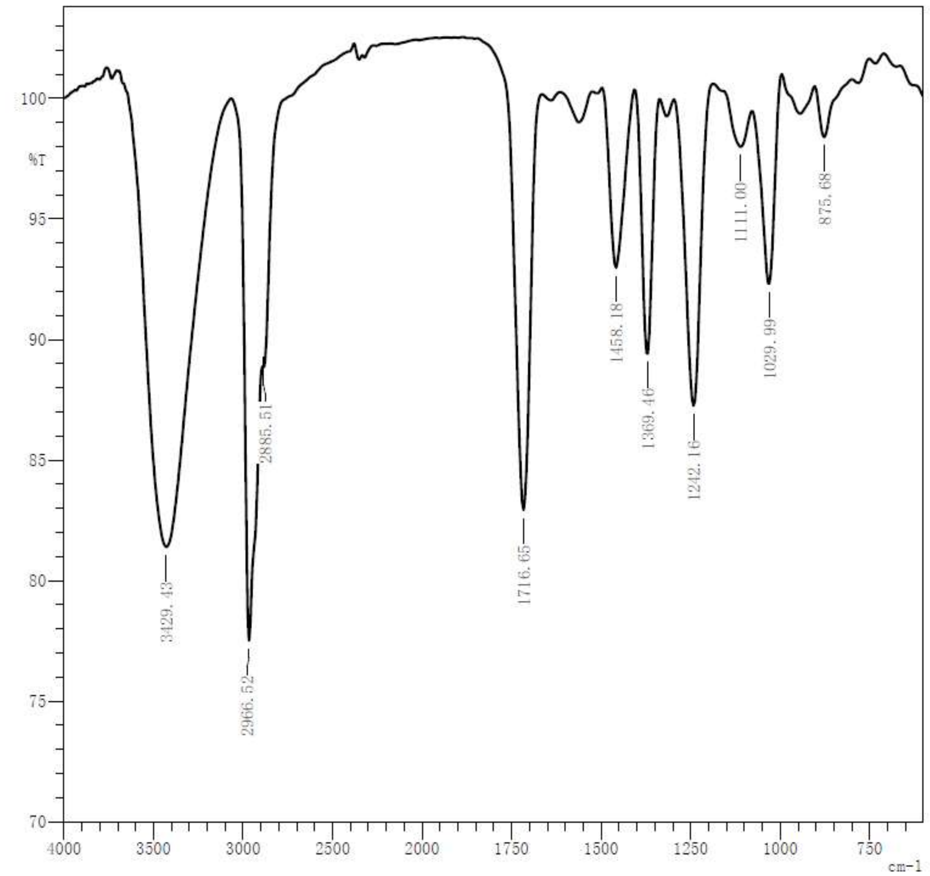

FigureS68. IR spectrum of compound **7**

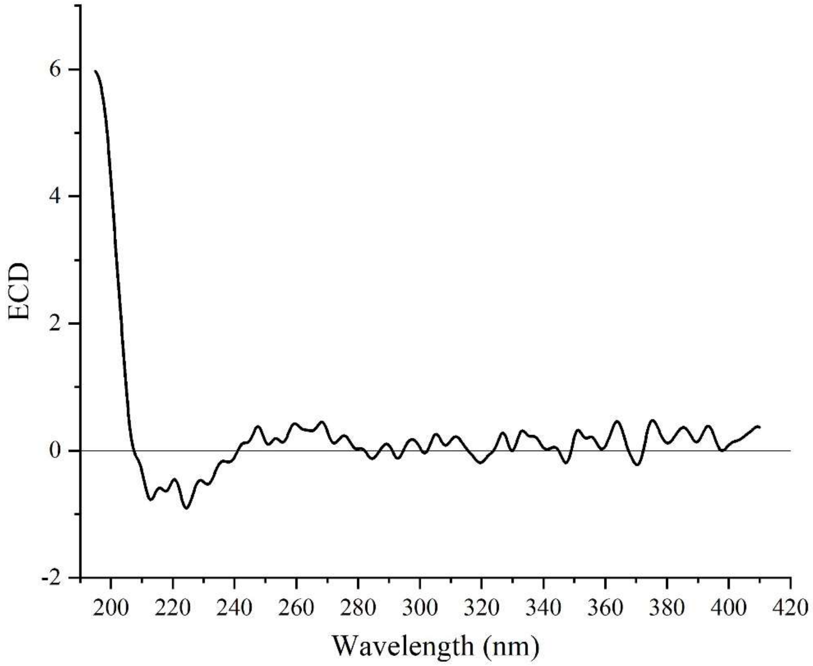

FigureS69. ECD spectrum of compound **7**

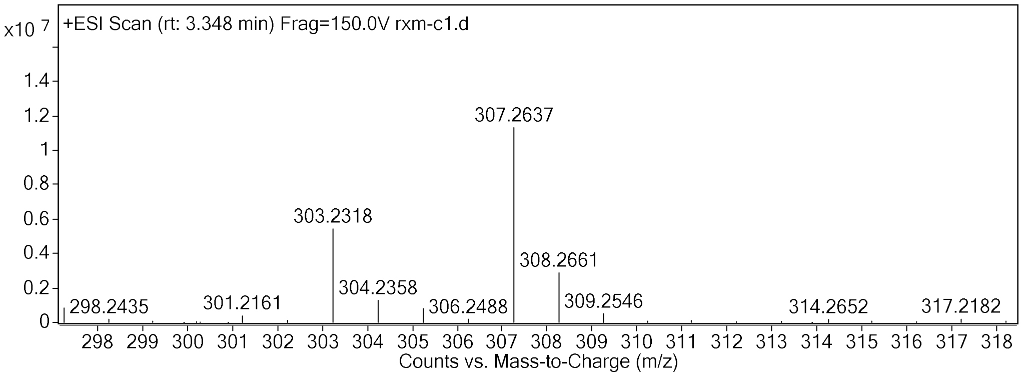

Formular	Score	Mass	Mass (MGF)	*m/z* (Calc)	Diff (ppm)	Ion Formular	*m/z*
$C_{20}H_{34}O_2$	99.64	306.2564	306.2559	307.2632	-1.77	$C_{20}H_{35}O_2$	307.2637

FigureS70. HRESIMS spectrum of compound **8**.

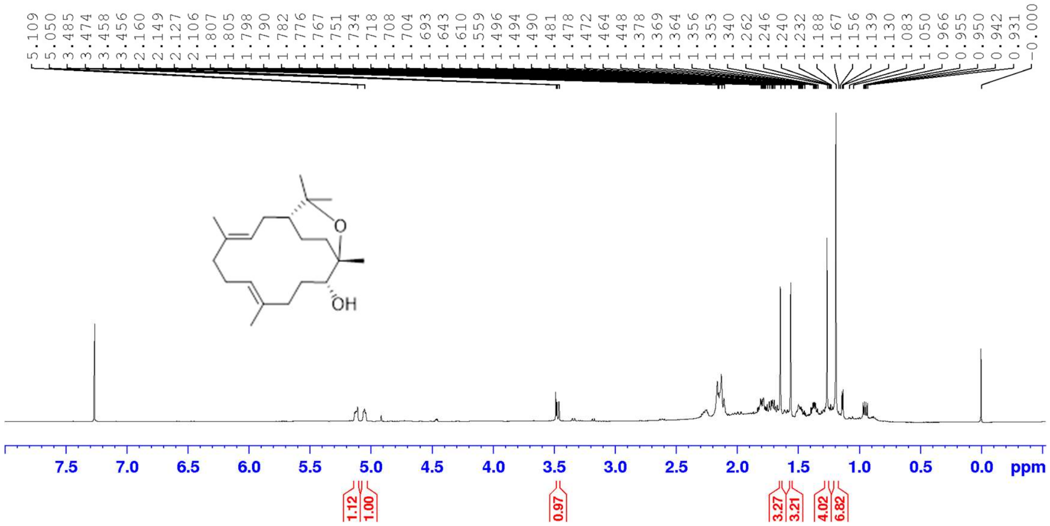

FigureS71. ^{1}H NMR spectrum of compound **8**

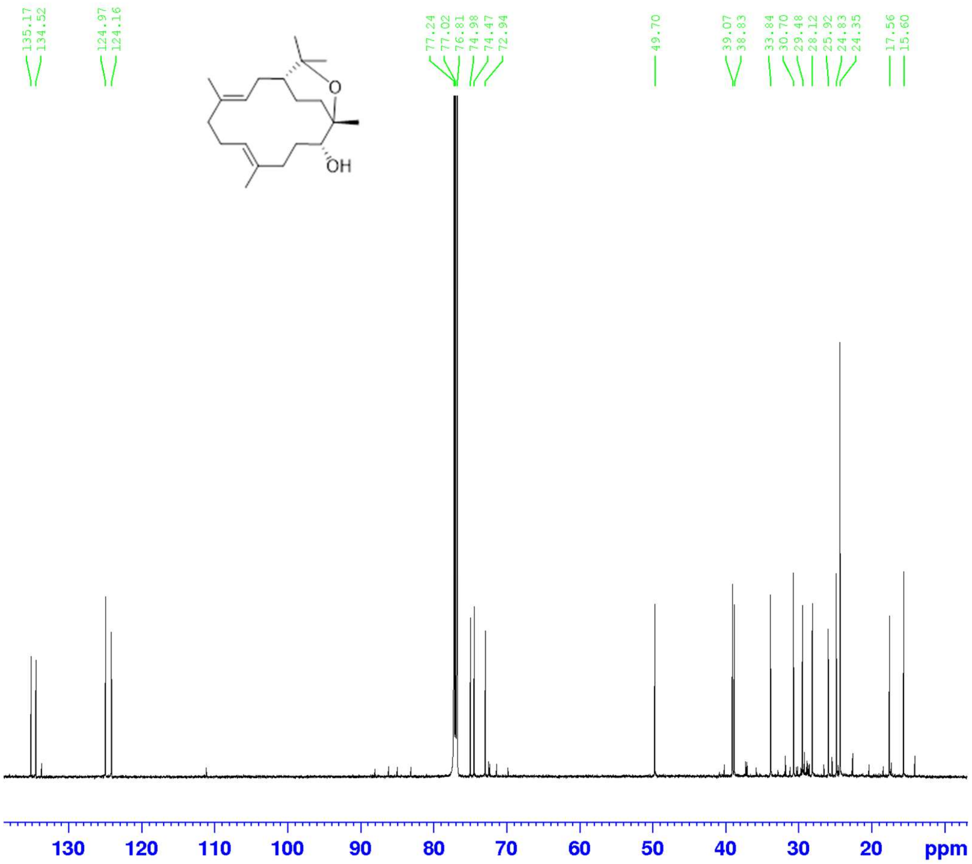

FigureS72. ^{13}C NMR spectrum of Compound **8**

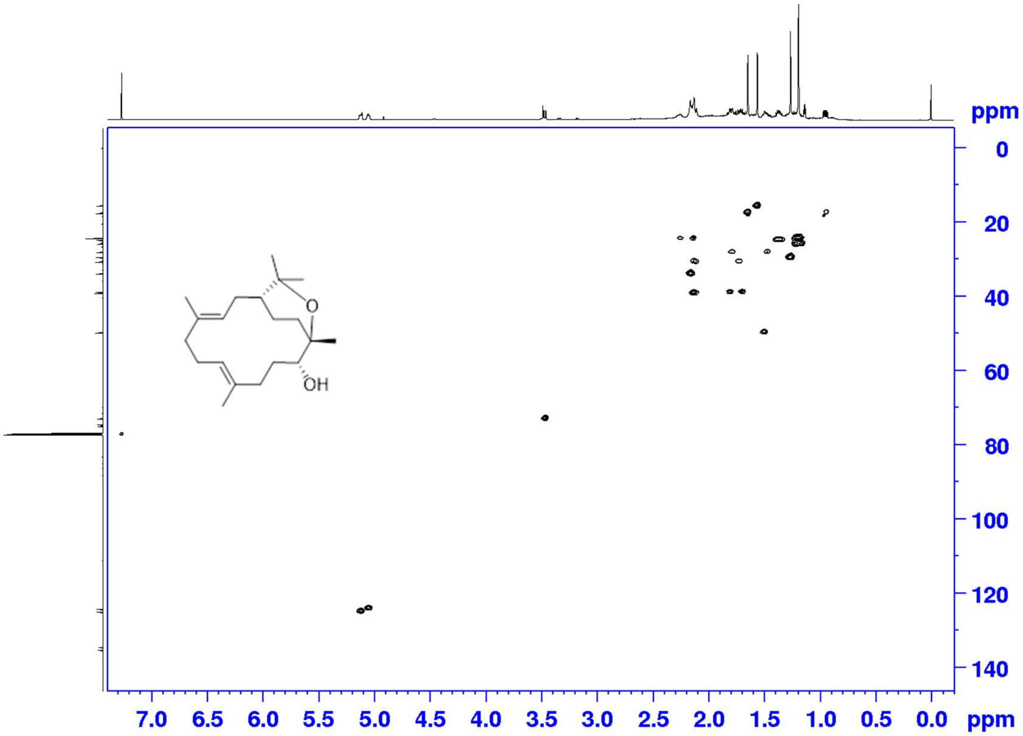

FigureS73. HSQC NMR spectrum of compound **8**

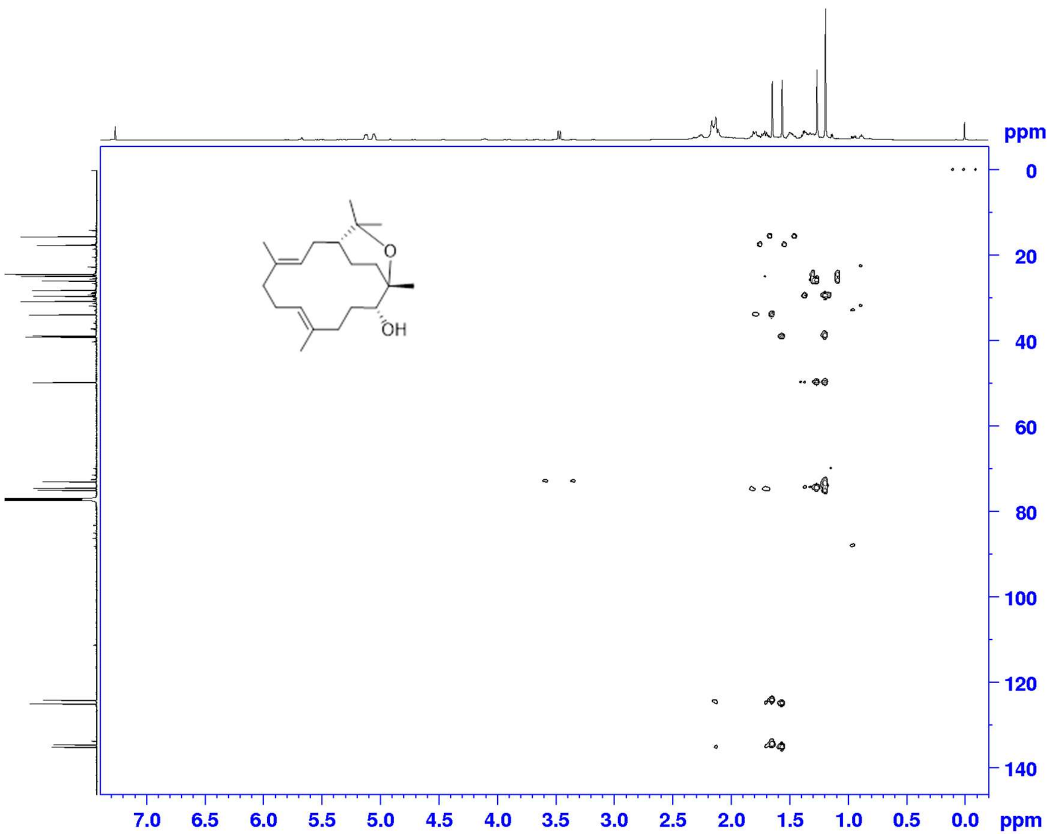

FigureS74. HMBC NMR spectrum of compound **8**

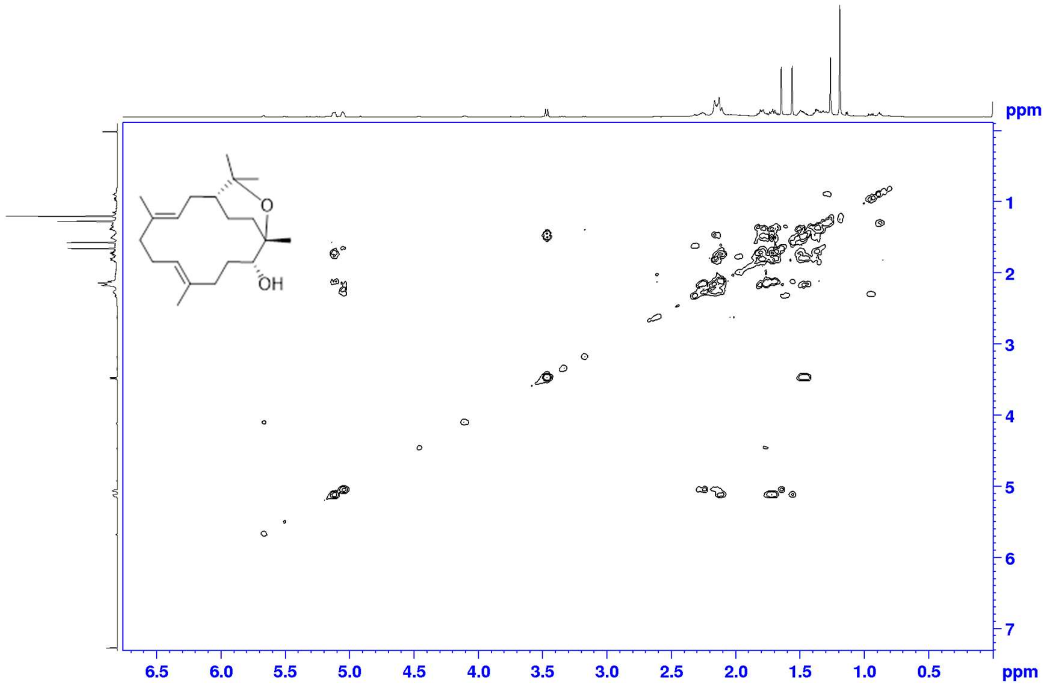

FigureS75. ^{1}H-^{1}H COSY NMR spectrum of compound **8**

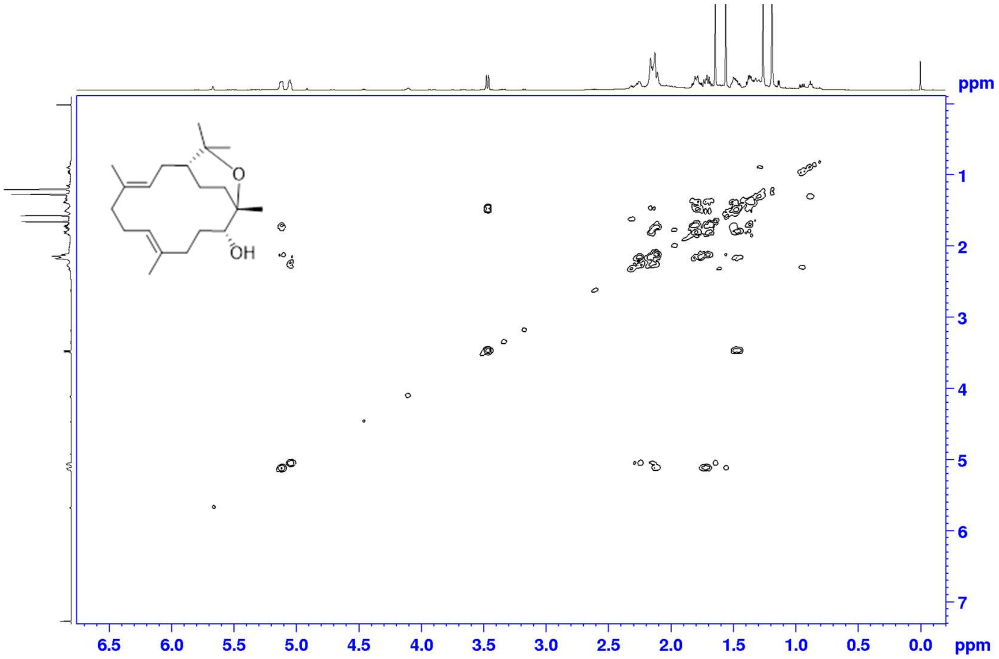

FigureS76. NOESY NMR spectrum of compound **8**

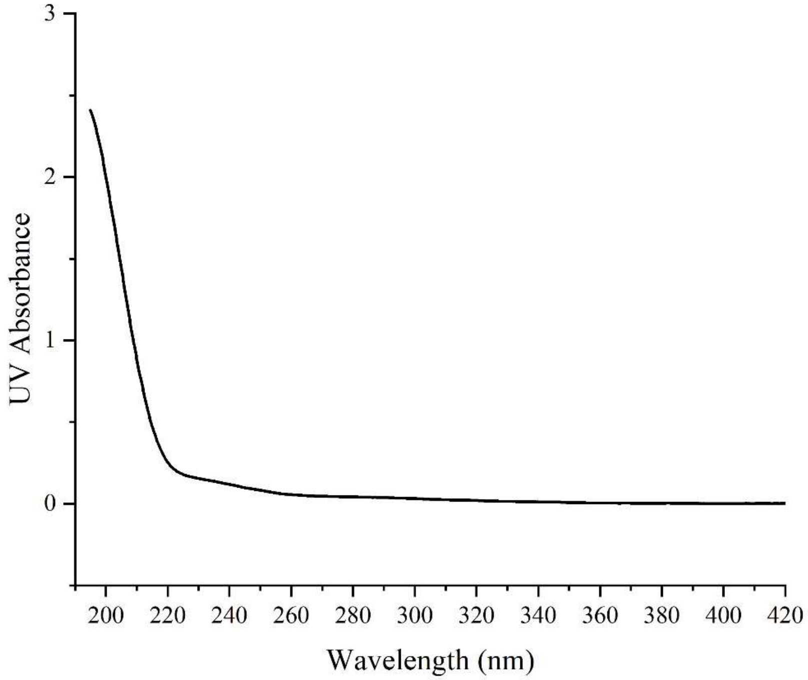

FigureS77. UV spectrum of compound **8**

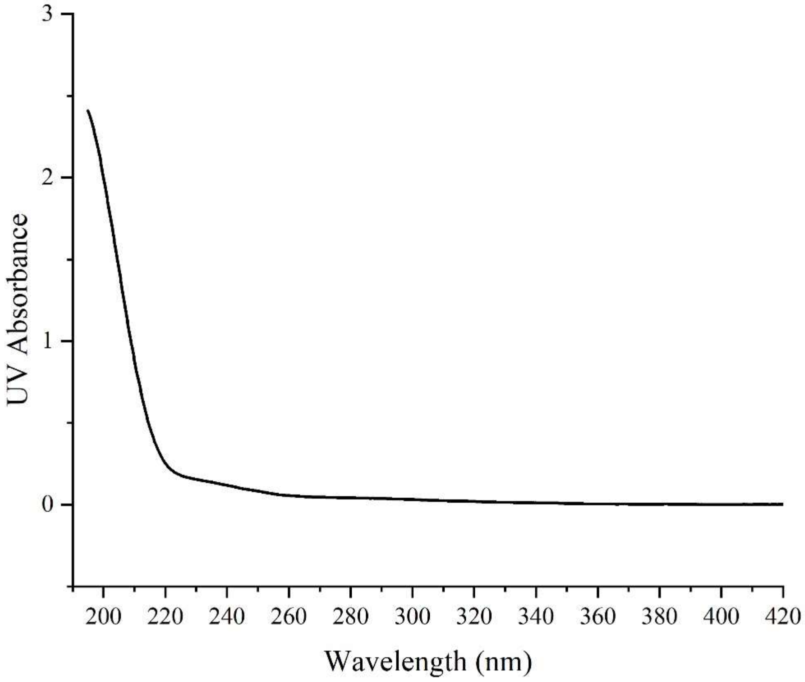

FigureS78. ECD spectrum of compound **8**

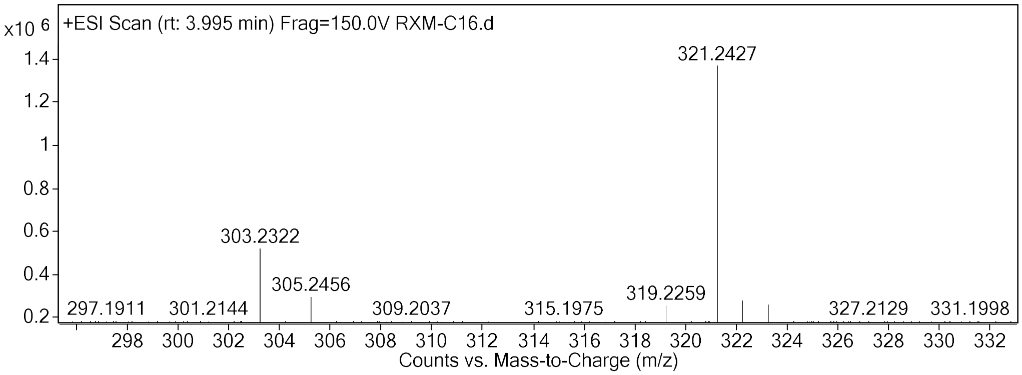

Formular	Score	Mass	Mass (MGF)	*m/z* (Calc)	Diff (ppm)	Ion Formular	*m/z*
$C_{20}H_{32}O_3$	99.91	320.2354	320.2351	321.2424	-0.87	$C_{20}H_{33}O_3$	321.2427

FigureS79. HRESIMS spectrum of compound **9**.

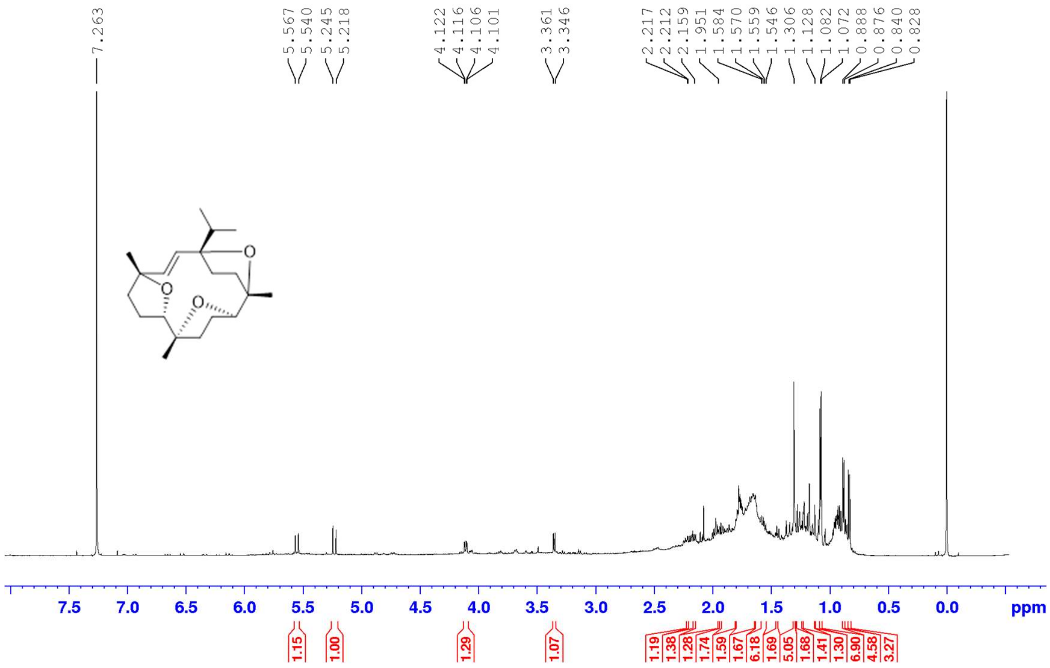

FigureS80. ^{1}H NMR spectrum of compound **9**

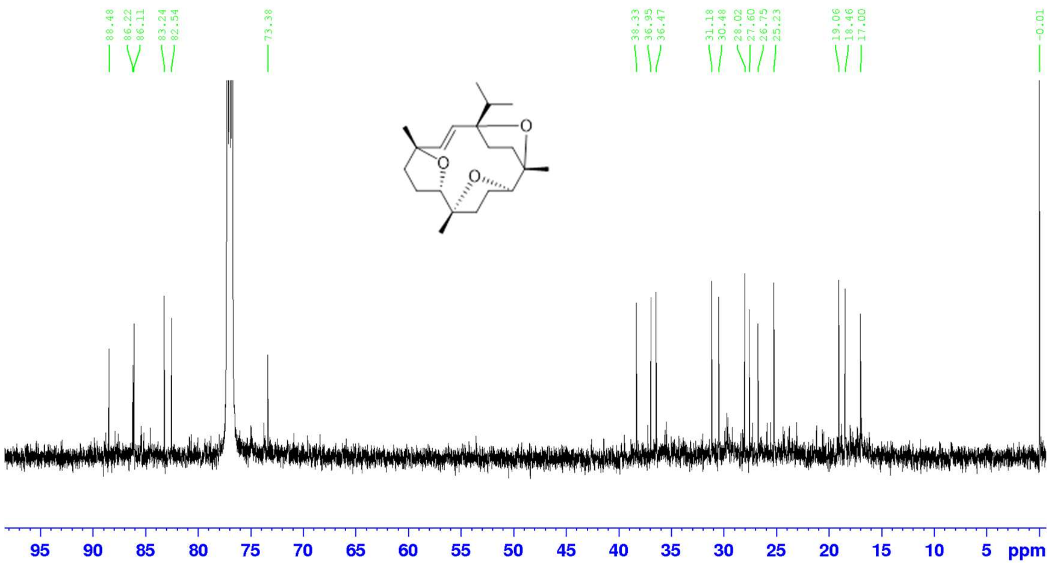

FigureS81. ^{13}C NMR spectrum of compound **9**

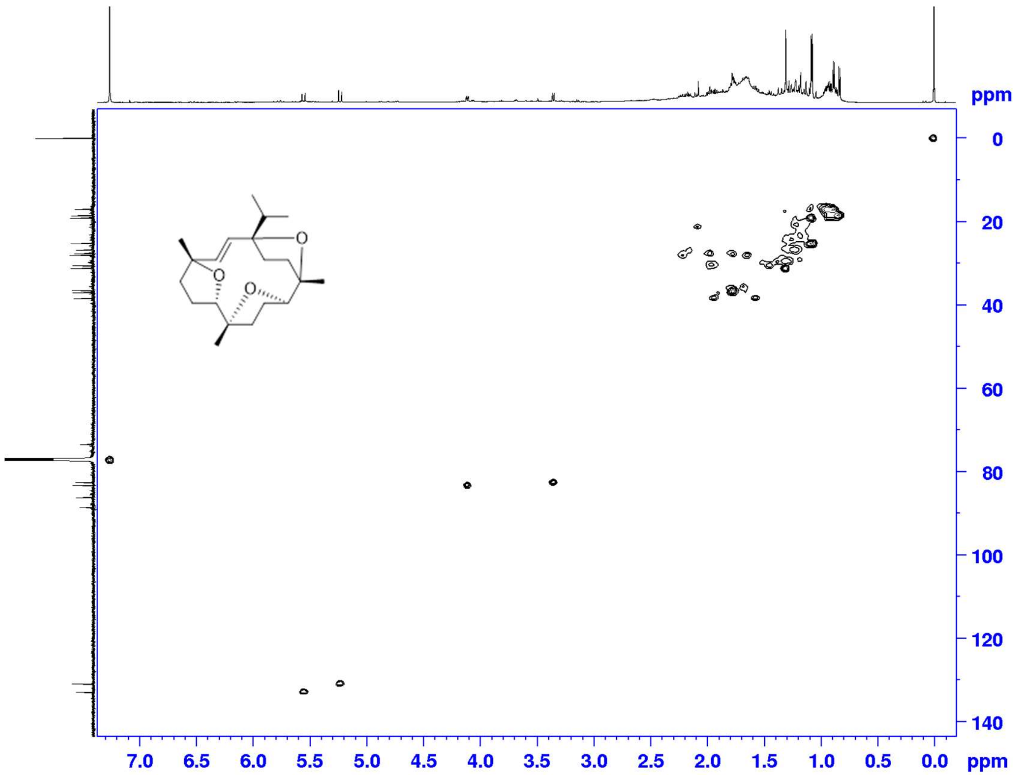

FigureS82. HSQC NMR spectrum of compound **9**

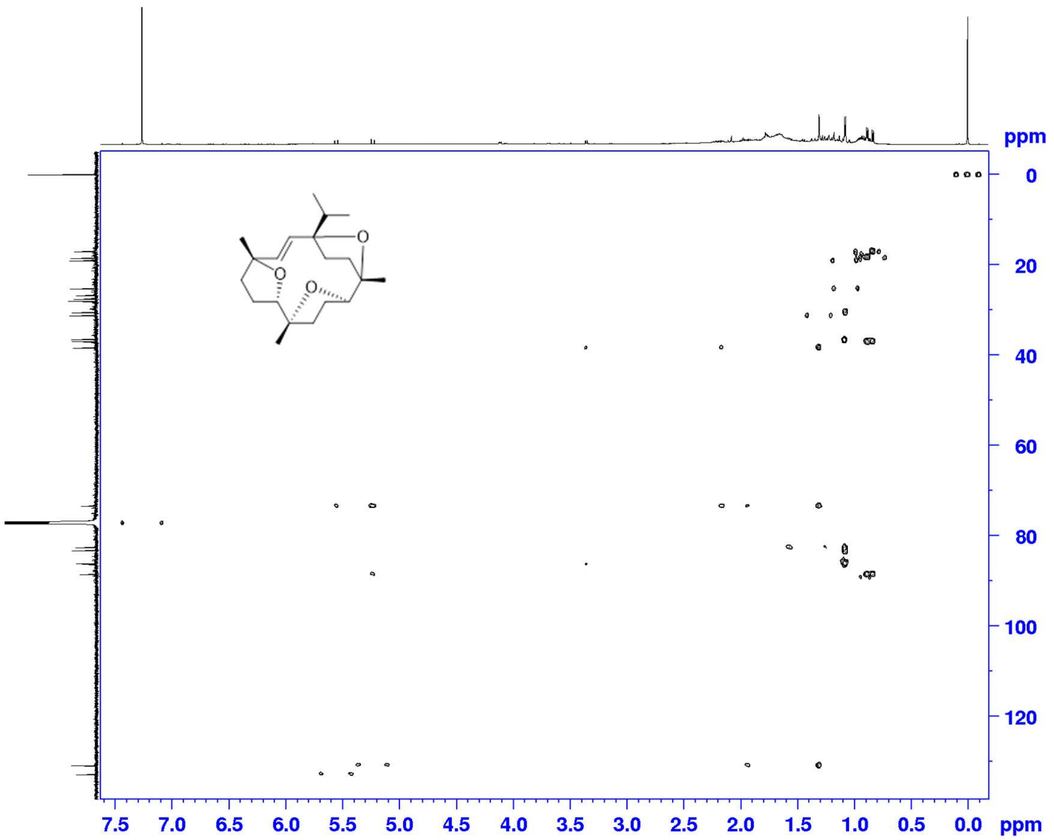

FigureS83. HMBC NMR spectrum of compound **9**

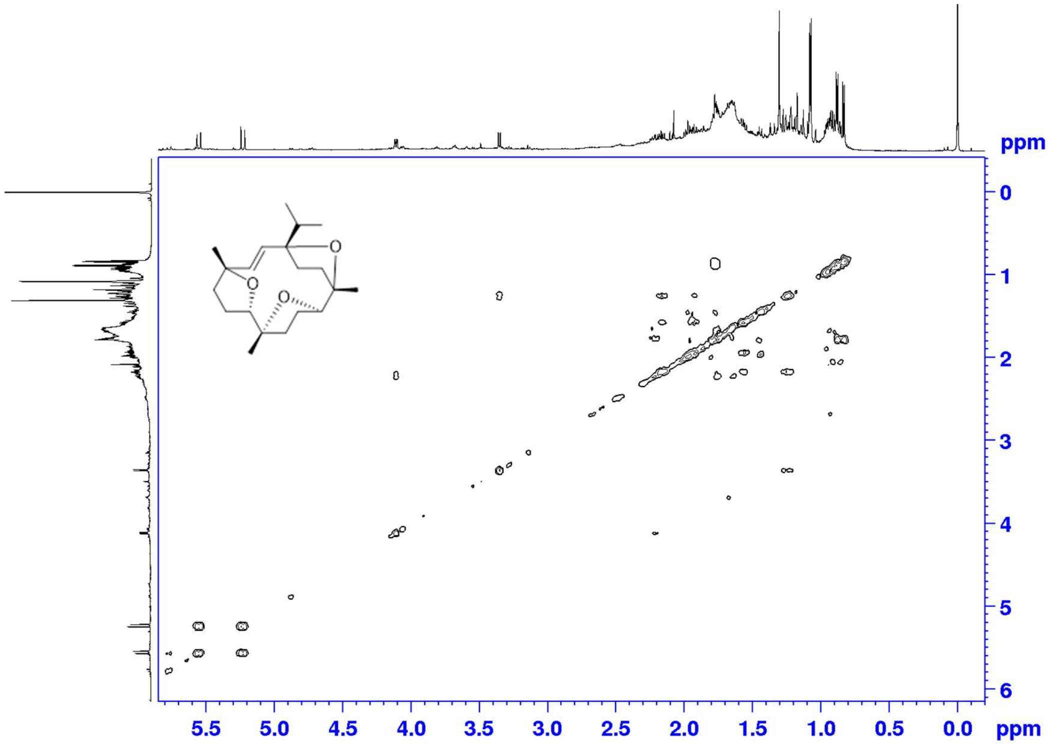

FigureS84. ^{1}H-^{1}H COSY NMR spectrum of compound **9**

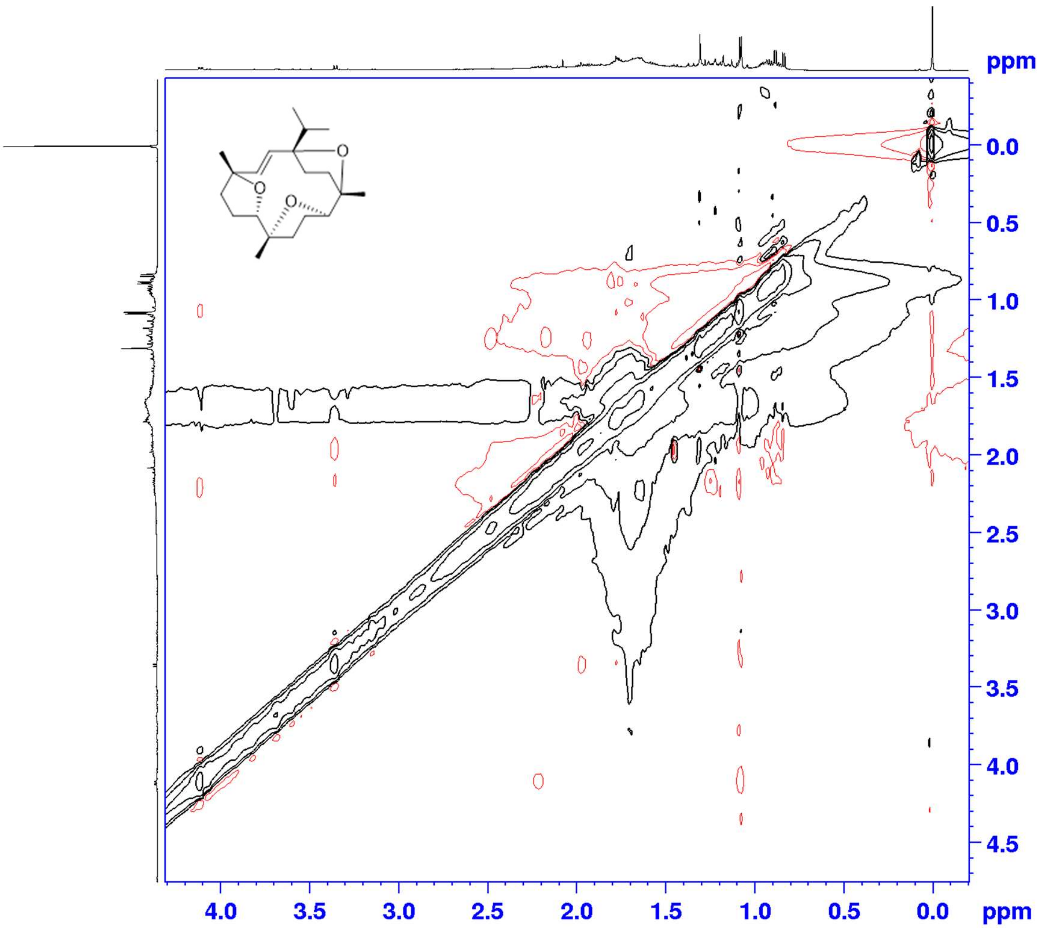

FigureS85. NOESY NMR spectrum of compound **9**

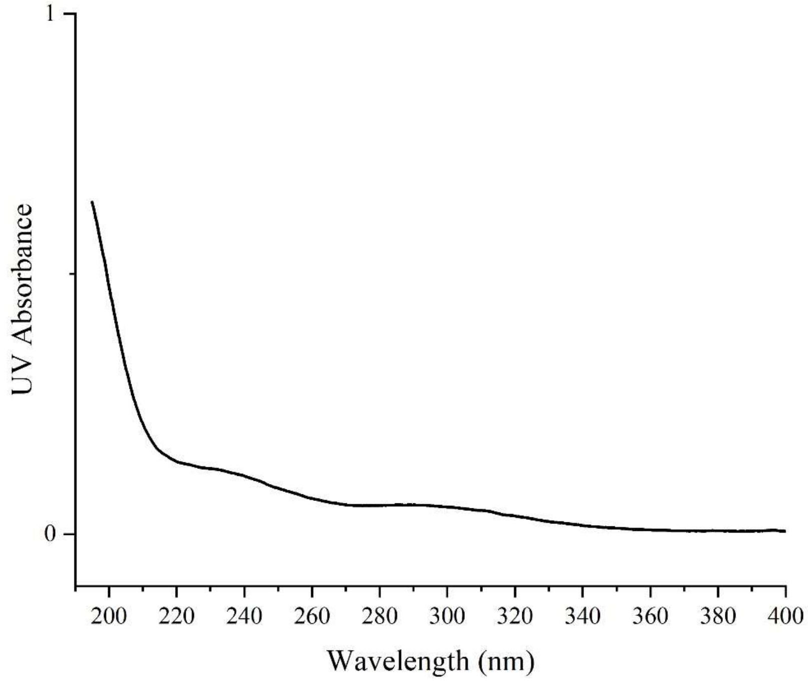

FigureS86. UV spectrum of compound **9**

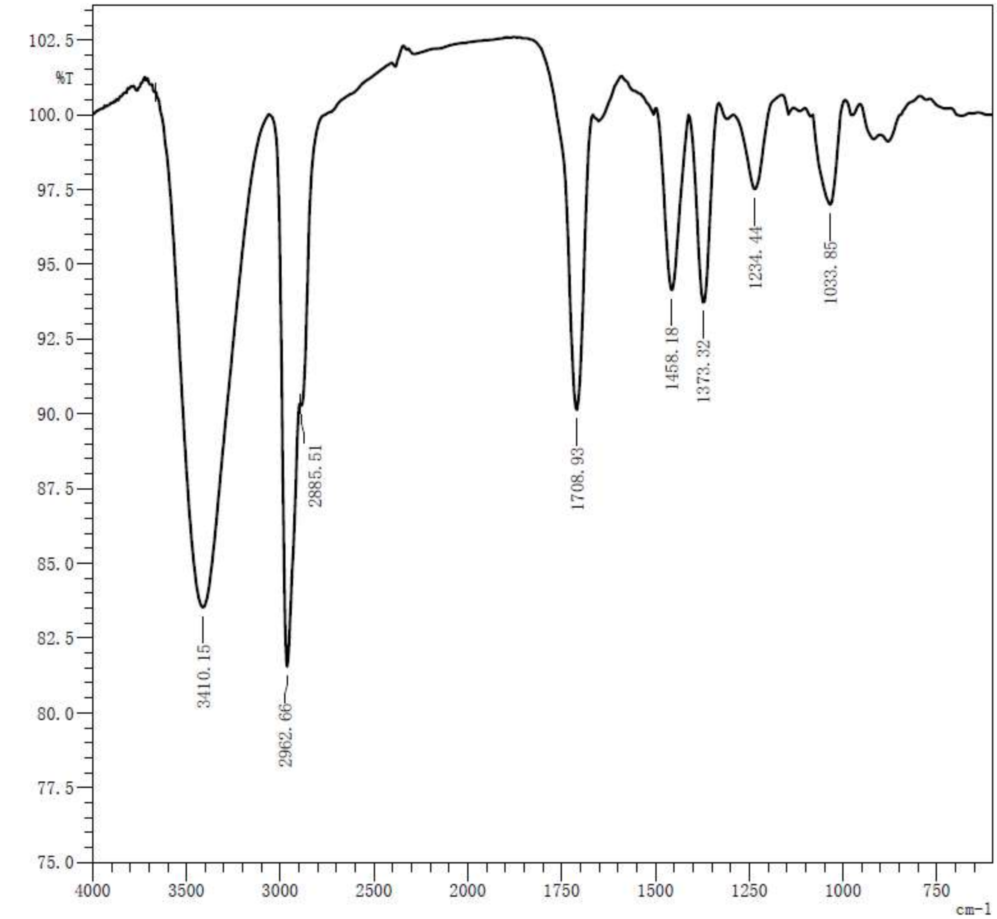

FigureS87. IR spectrum of compound **9**

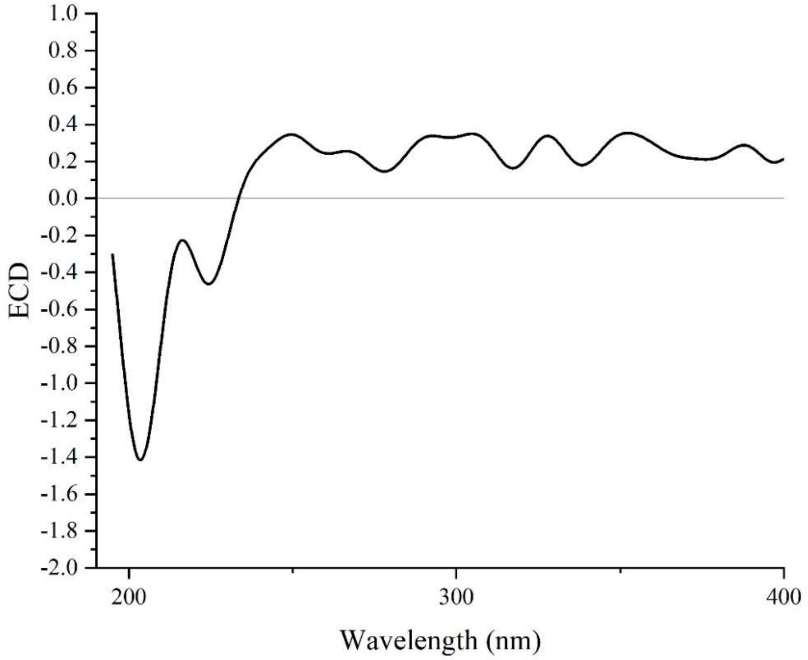

FigureS88. ECD spectrum of compound **9**

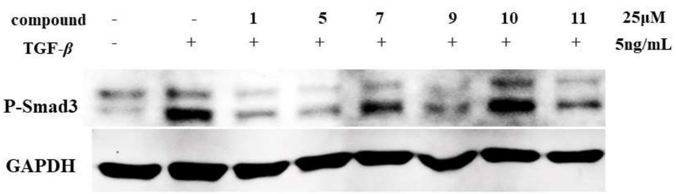

FigureS89. Western blot of TGF-β/Smad signal molecules for screening isolated compounds **1, 5, 7, 9, 10**, and **11** at 25μM. GADPH was used as an invariant control for equal loading.

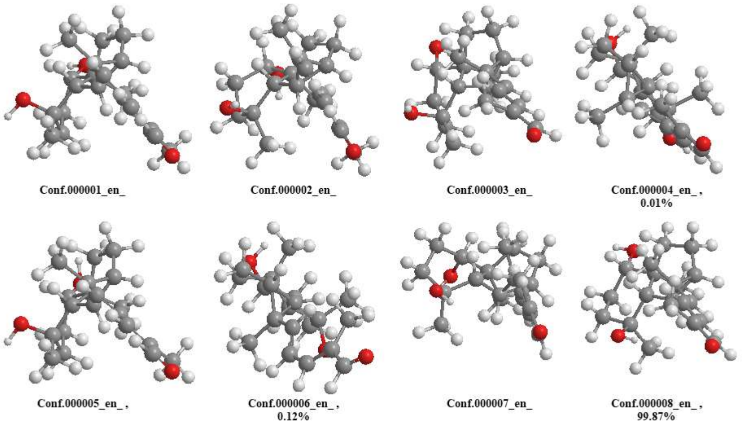

FigureS90. DFT computational optimized conformations of (1*R*,4*R*,5*R*,6*S*,7*S*,10*R*,13*R*)-**1**.

TableS1. Gibbs free energies[a] and equilibrium populations[b] of low-energy conformers of compound **1**.

Conformers	ΔG (a.u.)	P (%)/100	G (a.u.)
Conf.000001_en_	0.01365	0.0	-1005.373905
Conf.000002_en_	0.01278	0.0	-1005.374774
Conf.000003_en_	0.01159	0.0	-1005.375966
Conf.000004_en_	0.00892	0.01	-1005.378635
Conf.000005_en_	0.01303	0.0	-1005.374527
Conf.000006_en_	0.00632	0.12	-1005.381236
Conf.000007_en_	0.01335	0.0	-1005.374209
Conf.000008_en_	0.00000	99.87	-1005.387556

[a]wB97M-V/def2-TZVP, in a.u.

[b]From Δ*G* values at 298.15 K.

TableS2. Cartesian coordinates for the low-energy reoptimized random research conformers of compound **1** at B3LYP-D3(BJ)/6-31G* level of theory in methanol.

Conf.000004_en_		Standard Orientation (A.U.)			
Center number	Atomic number	Atomic Type	X	Y	Z
0	6	0	-6.411856	0.643691	-7.962031
1	6	0	-5.525125	-1.754469	-6.61434
2	6	0	-4.710425	-1.280432	-3.861802
3	6	0	-6.888961	0.100888	-2.486629
4	6	0	-7.898505	2.546685	-3.759552
5	6	0	-8.577284	1.933163	-6.543176
6	6	0	-6.355031	0.490205	0.307909
7	6	0	-7.92659	2.352141	1.807486
8	6	0	-9.959718	3.892998	0.470934
9	6	0	-10.349893	3.250761	-2.342567
10	6	0	-8.112397	-0.470267	2.34249
11	6	0	-2.121746	0.017988	-3.766268
12	8	0	-4.530285	-3.642302	-2.548362
13	8	0	-9.501172	4.066444	-7.870721
14	6	0	-6.050938	4.793809	-3.630494
15	1	0	-8.450024	-1.265909	-2.649891
16	1	0	-6.889531	3.417199	3.247378
17	1	0	-4.359361	0.431608	0.815324
18	6	0	-10.512234	-1.919817	1.714253
19	6	0	-4.736581	-2.719183	4.790306
20	6	0	-6.918348	-1.394937	4.725362
21	6	0	-3.447658	-3.74502	6.985429
22	6	0	-3.614927	-3.074276	9.461894
23	6	0	-5.229333	-1.01637	10.550219
24	1	0	-10.18096	0.60014	-6.49861
25	6	0	-1.993241	-4.492541	11.239295
26	8	0	-1.87027	-4.110906	13.495146
27	1	0	-4.824511	1.982278	-8.175783
28	1	0	-7.060699	0.201689	-9.889024
29	1	0	-3.960591	-2.647516	-7.666722
30	1	0	-7.080753	-3.139139	-6.559646
31	1	0	-11.78264	3.74467	1.473392
32	1	0	-9.404272	5.893708	0.622756
33	1	0	-11.676206	1.656872	-2.540834
34	1	0	-11.247056	4.855031	-3.314666
35	1	0	-2.032283	1.719866	-4.945271
36	1	0	-1.593196	0.542731	-1.828947
37	1	0	-0.674389	-1.300098	-4.48212
38	1	0	-3.188275	-4.613721	-3.317045
39	1	0	-8.105458	5.212925	-8.137418
40	1	0	-4.492665	4.638878	-4.993695
41	1	0	-7.054635	6.573094	-4.02487
42	1	0	-5.196473	4.968802	-1.746843
43	1	0	-10.050118	-3.885189	1.207493
44	1	0	-11.778774	-1.966563	3.367055
45	1	0	-11.575701	-1.082003	0.145692
46	1	0	-3.860911	-3.1486	2.962524
47	1	0	-7.993461	-1.058485	6.459102
48	1	0	-2.083687	-5.246966	6.554576
49	1	0	-5.270897	0.654982	9.310925

50	1	0	-7.200834	-1.647773	10.803662
51	1	0	-4.505524	-0.488663	12.423782
52	1	0	-0.825166	-6.009507	10.327543

Conf.000006_en_		Standard Orientation (A.U.)			
Center number	Atomic number	Atomic Type	X	Y	Z
0	6	0	-6.307867	1.007363	-7.979236
1	6	0	-5.054885	-1.262709	-6.708538
2	6	0	-4.36177	-0.777332	-3.93397
3	6	0	-6.751436	0.215602	-2.549649
4	6	0	-8.161057	2.49197	-3.757134
5	6	0	-8.68492	1.858509	-6.570278
6	6	0	-6.309606	0.626353	0.263946
7	6	0	-8.209828	2.073268	1.789611
8	6	0	-10.479337	3.268742	0.478785
9	6	0	-10.717493	2.697725	-2.363956
10	6	0	-7.900335	-0.763628	2.26809
11	6	0	-2.000692	0.892483	-3.74315
12	8	0	-3.781627	-3.210435	-2.912359
13	8	0	-9.951081	3.842799	-7.845561
14	6	0	-6.739013	5.01825	-3.52898
15	1	0	-8.056365	-1.38938	-2.76314
16	1	0	-7.433216	3.250445	3.30224
17	1	0	-4.338248	0.92358	0.806975
18	6	0	-9.935483	-2.607597	1.479381
19	6	0	-6.695838	-3.556795	5.949939
20	6	0	-6.453109	-1.415551	4.565578
21	6	0	-5.289784	-4.283958	8.186233
22	6	0	-3.292583	-3.166178	9.36529
23	6	0	-1.973109	-0.781128	8.589694
24	1	0	-10.031237	0.265224	-6.591806
25	6	0	-2.322738	-4.464619	11.637973
26	8	0	-0.555911	-3.749355	12.910681
27	1	0	-4.966657	2.601933	-8.11097
28	1	0	-6.840462	0.538094	-9.935243
29	1	0	-3.352026	-1.856092	-7.747344
30	1	0	-6.358492	-2.887062	-6.735073
31	1	0	-12.248412	2.749644	1.451316
32	1	0	-10.290236	5.326672	0.727562
33	1	0	-11.746929	0.911116	-2.659391
34	1	0	-11.86623	4.166488	-3.283487
35	1	0	-0.394381	-0.155775	-4.543328
36	1	0	-2.191484	2.6799	-4.776912
37	1	0	-1.529662	1.360048	-1.771783
38	1	0	-3.417819	-3.00793	-1.133579
39	1	0	-8.746269	5.181539	-8.144554
40	1	0	-5.124793	5.154628	-4.827164
41	1	0	-8.020622	6.601723	-3.950485
42	1	0	-6.00068	5.313714	-1.610918
43	1	0	-9.104175	-4.434211	0.924526
44	1	0	-11.255007	-2.946218	3.051944
45	1	0	-11.053515	-1.917937	-0.119174
46	1	0	-8.121228	-4.929274	5.355816
47	1	0	-5.064921	-0.001276	5.134575

48	1	0	-5.948069	-6.046737	9.05679
49	1	0	-3.228924	0.876391	8.742401
50	1	0	-0.33734	-0.471505	9.829445
51	1	0	-1.315389	-0.877617	6.614597
52	1	0	-3.371333	-6.238862	12.134952

Conf.000008_en_		Standard Orientation (A.U.)			
Center number	Atomic number	Atomic Type	X	Y	Z
0	6	0	-10.679822	-0.121417	-7.163672
1	6	0	-11.355287	-1.751796	-4.878877
2	6	0	-9.052642	-2.409205	-3.241351
3	6	0	-7.655709	0.084631	-2.573223
4	6	0	-6.963446	1.872678	-4.802052
5	6	0	-9.380174	2.33457	-6.367644
6	6	0	-5.40315	-0.33539	-0.828248
7	6	0	-3.517448	1.745702	-0.448641
8	6	0	-3.780243	4.205015	-1.921266
9	6	0	-6.151051	4.403685	-3.593212
10	6	0	-5.220185	0.97833	1.762901
11	6	0	-7.447193	-4.463447	-4.505852
12	8	0	-10.080674	-3.408003	-0.950762
13	8	0	-8.727253	3.854347	-8.473299
14	6	0	-4.816853	0.883703	-6.496427
15	1	0	-9.111014	1.108825	-1.49846
16	1	0	-1.552335	1.142943	-0.222925
17	1	0	-4.607035	-2.241766	-0.866353
18	6	0	-7.304229	2.712179	2.668864
19	6	0	-4.502254	-0.597275	6.228495
20	6	0	-3.961207	-0.547086	3.729201
21	6	0	-3.141013	-2.156034	8.003549
22	6	0	-3.559186	-2.280134	10.53686
23	6	0	-5.544735	-0.797345	11.927626
24	1	0	-10.713656	3.370438	-5.120191
25	6	0	-1.933785	-3.991191	12.014819
26	8	0	-2.065362	-4.306033	14.28223
27	1	0	-9.445164	-1.151222	-8.48592
28	1	0	-12.413009	0.343156	-8.228297
29	1	0	-12.305846	-3.507941	-5.46409
30	1	0	-12.697573	-0.731946	-3.654932
31	1	0	-3.682604	5.844416	-0.637141
32	1	0	-2.098928	4.34828	-3.139596
33	1	0	-7.760835	5.126756	-2.48576
34	1	0	-5.824398	5.795808	-5.101412
35	1	0	-5.73682	-4.904363	-3.407005
36	1	0	-8.584408	-6.199469	-4.621457
37	1	0	-6.838547	-3.951538	-6.42032
38	1	0	-8.6947	-3.763238	0.184926
39	1	0	-10.238302	4.163321	-9.447036
40	1	0	-3.233013	0.155591	-5.367729
41	1	0	-5.435578	-0.638055	-7.761082
42	1	0	-4.11139	2.416875	-7.70504
43	1	0	-8.036534	3.922738	1.159759
44	1	0	-8.901192	1.612736	3.42836
45	1	0	-6.603714	3.964059	4.176045

46	1	0	-6.019962	0.587592	6.972845
47	1	0	-2.42314	-1.765502	3.055406
48	1	0	-1.624621	-3.337191	7.223852
49	1	0	-5.259853	1.255239	11.713603
50	1	0	-7.458531	-1.234199	11.230466
51	1	0	-5.460392	-1.263529	13.948515
52	1	0	-0.495755	-5.0355	10.856843
